# Design, synthesis, antiviral bioactivities and interaction mechanisms of penta-1,4-diene-3-one oxime ether derivatives containing a quinazolin-4(3*H*)-one scaffold

**DOI:** 10.1186/s13065-019-0547-1

**Published:** 2019-03-25

**Authors:** Lijuan Chen, Xiaobin Wang, Xu Tang, Rongjiao Xia, Tao Guo, Cheng Zhang, Xiangyang Li, Wei Xue

**Affiliations:** 1 State Key Laboratory Breeding Base of Green Pesticide and Agricultural Bioengineering, Key Laboratory of Green Pesticide and Agricultural Bioengineering, Ministry of Education, Guizhou University, Guiyang, 550025 China; 2 College of Sciences, Nanjing Agricultural University, Nanjing, 210095 China

**Keywords:** Penta-1,4-diene-3-one, Oxime ether, Quinazolin-4(3*H*)-one, Antiviral activity, Tobacco mosaic virus, Interaction mechanisms, Molecular docking study

## Abstract

**Background:**

penta-1,4-diene-3-one oxime ether and quinazolin-4(3*H*)-one derivatives possess favorable agricultural activities. Aiming to discover novel molecules with highly-efficient agricultural activities, a series of penta-1,4-diene-3-one oxime ether derivatives containing a quinazolin-4(3*H*)-one scaffold were synthesized and evaluated for their antiviral activities.

**Result:**

Antiviral bioassays indicated that some title compounds exhibited significant antiviral activity against tobacco mosaic virus (TMV). In particular, compounds **8c**, **8j** and **8k** possessed appreciable curative activities against TMV in vivo, with half-maximal effective concentration (EC_50_) values of 138.5, 132.9 and 125.6 μg/mL, respectively, which are better than that of ningnanmycin (207.3 μg/mL). Furthermore, the microscale thermophoresis experiments (MST) on the interaction of compound **8k** with TMV coat protein (TMV CP) showed **8k** bound to TMV CP with a dissociation constant of 0.97 mmol/L. Docking studies provided further insights into the interaction of **8k** with the Arg90 of TMV CP.

**Conclusions:**

Sixteen penta-1,4-diene-3-one oxime ether derivatives containing a quinazolin-4(3*H*)-one scaffold were designed, synthesized, and their antiviral activities against TMV were evaluated. Antiviral bioassays indicated that some target compounds exhibited remarkable antiviral activities against TMV. Furthermore, through the MST and docking studies, we can speculate that **8k** inhibited the virulence of TMV by binding Arg90 in TMV CP. These results indicated that this kind of penta-1,4-diene-3-one oxime ether derivatives containing a quinazolin-4(3*H*)-one scaffold could be further studied as potential alternative templates in the search for novel antiviral agents.
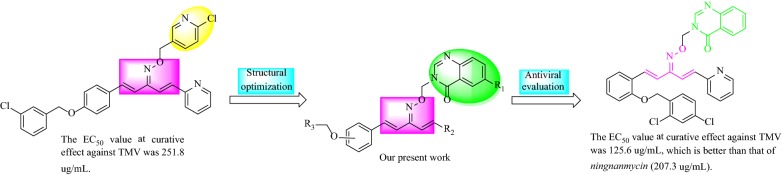

**Electronic supplementary material:**

The online version of this article (10.1186/s13065-019-0547-1) contains supplementary material, which is available to authorized users.

## Background

Tobacco mosaic virus (TMV) is an important plant virus that caused serious crop loss [1], and unfortunately, there are few effective antiviral agents to control the infection of TMV [2]. Ningnanmycin, exhibited a dual targeting ability against the TMV coat protein [3], is the best anti-TMV agent that was isolated from *Streptomyces noursei* var. [4]. However, its application in field trail is largely limited by its photosensitivity, water stickiness and high control costs [5, 6]. Therefore, developing novel, highly-efficient, and environmentally benign antiviral agents remains a daunting task in pesticide sciences.

**Fig. 1:**
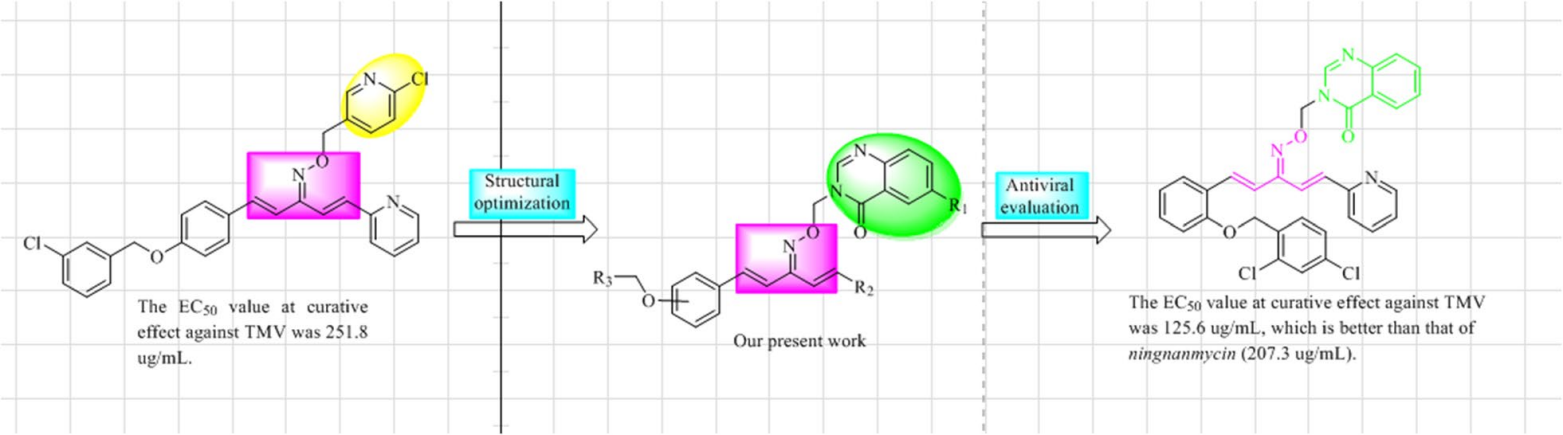
Design strategy for target molecules

Natural product-based compounds consistently display more advantages than traditional synthetic chemicals, such as low toxicity, easy decomposition, environmental friendliness and unique modes of action [7–10]. As important analogs of curcumin isolated from turmeric, penta-1,4-diene-3-ones are viewed as lead compounds due to their significant applications in the development of pharmaceuticals and agrochemicals [11–15]. In the last decade, series of penta-1,4-diene-3-one derivatives bearing pyrazole [16], quinazoline [17], quinazolin-4(3*H*)-one [18], 1,3,4-thiadiazole [19], 1,3,4-oxadiazole [20], emodin [21], glucopyranoside [22], oxime ether [23], oxime ester [15], pyridine [24], rutin [25], 4-thioquinazoline [26] and purine [27] moieties were reported for their fine antiviral activities against plant viruses (Fig. 1). However, the antiviral activities of these penta-1,4-diene-3-one derivatives were adequate but still not satisfactory.

Quinazolin-4(3*H*)-ones, important nitrogenous heterocycles that present extensive bioactivities including antibacterial [28], anti-inflammatory [29], antifungal [30], antimalarial [31] and anticancer [32] properties, were reported for their potent antiviral activities against plant viruses. Recently, Ma et al. evaluated antiviral activities of quinazolin-4(3*H*)-one derivatives containing penta-1,4-diene-3-one moiety, and found these compounds can effectively control some plant viruses [18]. Chen et al. reported that cucumber mosaic virus could be inhibited effectively by some quinazolin-4(3*H*)-one derivatives containing malonate moiety [33].

To develop novel, highly-efficient and environmentally benign virucides, we introduced a quinazolin-4(3*H*)-one scaffold into penta-1,4-diene-3-one oxime ether derivatives, which might generate some novel curcumin derivatives with potent biological activities. Thus, 16 penta-1,4-diene-3-one oxime ether derivatives containing a quinazolin-4(3*H*)-one scaffold were designed, synthesized and evaluated for their antiviral activity against TMV in vivo.

## Results and discussion

### Chemistry

The infrared spectrum (IR), ^1^H nuclear magnetic resonance (^1^H NMR), ^13^C nuclear magnetic resonance (^13^C NMR) and high resolution mass spectrum (HRMS) spectra of title compounds are shown in Additional file 1. IR spectra exhibit characteristic absorptions at 1650–1500 cm^−1^, which indicate the presence of –C=N– fragment. The signals at 1750–1650 and 1260–1210 cm^−1^ are attributed to the stretching frequencies of –C=O and –C–O–N=groups, respectively. In ^1^H NMR spectra, multiplet signals at δ 8.20–6.50 ppm show the presence of protons in olefinic bonds and aromatic nucleuses, and a singlet at δ 5.50–5.00 ppm reveals the presence of –CH_2_– groups. Absorption signals at δ 155–165 and 65–75 ppm in ^13^C NMR spectra confirm the presences of –C=N– and –CH_2_– groups, respectively. Noteworthily, the ^1^H NMR and ^13^C NMR spectra of title compounds also reveal that title compounds don’t have chemical isomers, which are consist with a result described in our previous work [22]. In the HRMS spectra of target compounds show characteristic absorption signals of [M+H]^+^ ions that is consistent with their molecular weight.

### Antiviral activity screening of title compounds against TMV in vivo

Using *N. tabacun* L. leaves under the same age as the test subjects, the curative and protective activities against TMV in vivo at 500 μg/mL were evaluated by the half-leaf blight spot method [14, 18, 34, 35] and the obtained results were listed in Table 1. Preliminary bioassays indicated that the curative and protective effects of title compounds against TMV at 500 μg/mL ranged 31.7–59.1% and 39.4–69.8%, respectively. Among them, the curative effects of compounds **8c**, **8d**, **8e**, **8j** and **8k** against TMV were 55.9, 53.4, 54.6, 55.4 and 59.1%, respectively, which are better than that of ningnanmycin (51.8%). In addition, compounds **8c**, **8j** and **8k** exhibited significant protection effects against TMV at 500 μg/mL, with corresponding inhibitory rates of 69.8, 67.7 and 72.0%, respectively, which are superior to that of ningnanmycin (65.7%).

**Table 1:** Antiviral effects of title compounds against TMV in vivo at 500 μg/mL

Compound	R_1_	R_2_	R_3_	–O–	Curative activity(%)^a^	Protective activity(%)^a^
**8a**	H	Pyridin-2-yl	3-ClPh	4-O	44.6 ± 1.7	59.4 ± 1.6
**8b**	H	Pyridin-2-yl	3-MePh	4-O	40.1 ± 2.4	56.4 ± 1.3
**8c**	H	Pyridin-2-yl	2-ClPh	4-O	55.9 ± 2.9	69.8 ± 5.8
**8d**	H	Pyridin-2-yl	4-ClPh	4-O	53.4 ± 3.9	44.7 ± 3.2
**8e**	H	Pyridin-2-yl	2,4-di-ClPh	4-O	54.6 ± 4.5	60.1 ± 3.4
**8f**	Cl	Pyridin-2-yl	3-MePh	4-O	31.7 ± 3.8	39.4 ± 4.0
**8g**	Cl	Pyridin-2-yl	3-ClPh	4-O	33.4 ± 5.6	55.4 ± 4.9
**8h**	Cl	Pyridin-2-yl	2-ClPh	4-O	44.9 ± 4.7	52.1 ± 4.1
**8i**	Cl	Pyridin-2-yl	4-ClPh	4-O	45.3 ± 4.8	47.7 ± 6.3
**8j**	H	Pyridin-2-yl	2-ClPh	2-O	55.4 ± 4.3	67.7 ± 1.7
**8k**	H	Pyridin-2-yl	2,4-di-ClPh	2-O	59.1 ± 4.2	72.0 ± 3.9
**8l**	Cl	Pyridin-2-yl	2-ClPh	2-O	45.8 ± 6.1	48.9 ± 4.3
**8m**	Cl	Pyridin-2-yl	2,4-di-ClPh	2-O	50.5 ± 4.9	54.4 ± 3.9
**8n**	H	Thiophene-2-yl	2,4-di-ClPh	4-O	37.3 ± 6.1	50.3 ± 5.1
**8o**	H	Pyridin-3-yl	2-ClPh	4-O	53.5 ± 2.5	65.1 ± 6.2
**8p**	H	Pyridin-3-yl	3-MePh	4-O	37.4 ± 3.6	53.2 ± 3.5
Ningnanmycin^b^	–	–	–	–	51.8 ± 4.3	65.7 ± 2.9

^a^Average of three replicates

^b^Ningnanmycin was used as a comparision

Based on the preliminary bioassays in Table 1, the curative activities of compounds **8c**, **8j** and **8k** against TMV at 500, 250, 125, 62.5 and 31.25 μg/mL were respectively tested to obtain the corresponding EC_50_ values. As showed in Table 2, compounds **8c**, **8j** and **8k** showed excellent curative effects against TMV, with corresponding EC_50_ values of 138.5, 132.9 and 125.6 μg/mL, respectively, which are better than that of ningnanmycin (207.3 μg/mL). These results indicated that this kind of penta-1,4-diene-3-one oxime ether derivatives containing a quinazolin-4(3*H*)-one scaffold could be further studied as potential alternative templates in the search for novel antiviral agents.

**Table 2:** Curative effects of compounds **8c**, **8j** and **8k** against TMV

Compound	Concentration (μg/mL)^a^	Curative activity (%)^a^	Toxic regression equation	r	EC_50_ (μg/mL)
**8c**	500	55.9 ± 2.9	y = 0.3444x + 4.2222	0.9860	138.5
	250	51.2 ± 5.5			
	125	48.8 ± 4.9			
	62.5	44.2 ± 5.6			
	31.25	38.9 ± 5.0			
**8j**	500	55.4 ± 4.3	y = 0.3111x + 4.3085	0.9746	132.9
	250	52.2 ± 5.1			
	125	48.7 ± 5.3			
	62.5	46.1 ± 5.6			
	31.25	39.9 ± 4.8			
**8k**	500	59.1 ± 4.2	y = 0.4154x + 4.1258	0.9836	125.6
	250	56.1 ± 5.6			
	125	50.0 ± 3.8			
	62.5	43.6 ± 3.5			
	31.25	40.6 ± 5.1			
Ningnamycin^b^	500	51.6 ± 4.3	y = 0.5475x + 3.4987	0.9761	207.3
	250	41.2 ± 3.8			
	125	33.3 ± 2.6			
	62.5	30.3 ± 4.6			
	31.25	26.2 ± 3.4			

^a^Average of three replicates

^b^Ningnanmycin was used as a comparision

### Structure activity relationship (SAR) of title compounds against TMV

As indicated in Tables 1 and 2, most of penta-1,4-diene-3-one oxime ether derivatives containing a quinazolin-4(3*H*)-one scaffold showed significant antiviral activities against TMV, and some structure–activity relationships can be analyzed and summarized. First, most of the target compounds bearing the same substituted fragment exhibited higher protective activity than curative activity against TMV. For example, the protective effects of compounds **8a**, **8b**, **8c**, **8e**, **8f**, **8g**, **8h**, **8i**, **8j**, **8k**, **8l**, **8m**, **8n**, **8o** and **8p** are 59.4, 56.4, 69.8, 60.1, 39.4, 55.4, 52.1, 47.7, 67.7, 72.0, 48.9, 54.4, 50.3, 65.1 and 53.2%, respectively, which were better than their curative effects (44.6, 40.1, 55.9, 54.6, 31.7, 33.4, 44.9, 45.3, 55.4, 59.1, 45.8, 50.5, 37.3, 53.5 and 37.4%, respectively). Second, the presence of a quinazolin-4(3*H*)-one fragment can effectively enhance the anti-TMV activities of target compounds. For example, the curative activities of compounds **8a**, **8b**, **8c**, **8d**, **8j** and **8k** (R_1_=H) were 44.6, 40.1, 55.9, 53.4, 55.4 and 59.1%, respectively, which are better than that of compounds **8g**, **8f**, **8h**, **8i**, **8l** and **8m** (R_2_=Cl; 33.4, 31.7, 44.9, 45.3, 45.8 and 50.5%, respectively). Third, antiviral bioassays reveals that the pyridine fragment, not thiophene fragment, was favorable for the anti-TMV activities of penta-1,4-diene-3-one oxime ether derivatives. For instances, the curative inhibition of compound **8n** was 37.3%, which was lower than that of compounds **8k** (54.6%). Furthermore, when the R_3_ was substituted with 2-ClPh, 4-ClPh and 2,4-di-ClPh groups, the corresponding compounds **8c**, **8j** and **8k** showed better curative effects against TMV, with the EC_50_ values of 138.5, 132.9 and 125.6 μg/mL, respectively, which are better than that of ningnanmycin (207.3 μg/mL).

### The expression and purification of TMV CP

pET28a-TMV CP vector were constructed and preserved in our lab [36, 37]. TMV CP genes were overexpressed in pET28a-TMV CP vector when the final concentration was increased to 0.8 mM isopropoyl-β-d-galactopyranoside (IPTG) and the solution was left overnight at 16 °C. More than 90% of the TMV CP protein was eluted. Then, the dealt proteins were loaded in desalting column, in a buffer containing 10 mM PB and 100 mM sodium chloride pH 7.2, the retention time of the TMV CP protein was determined with 19.5 kDa with His-tags.

### The binding studies of TMV CP and 8k

**Fig. 2:**
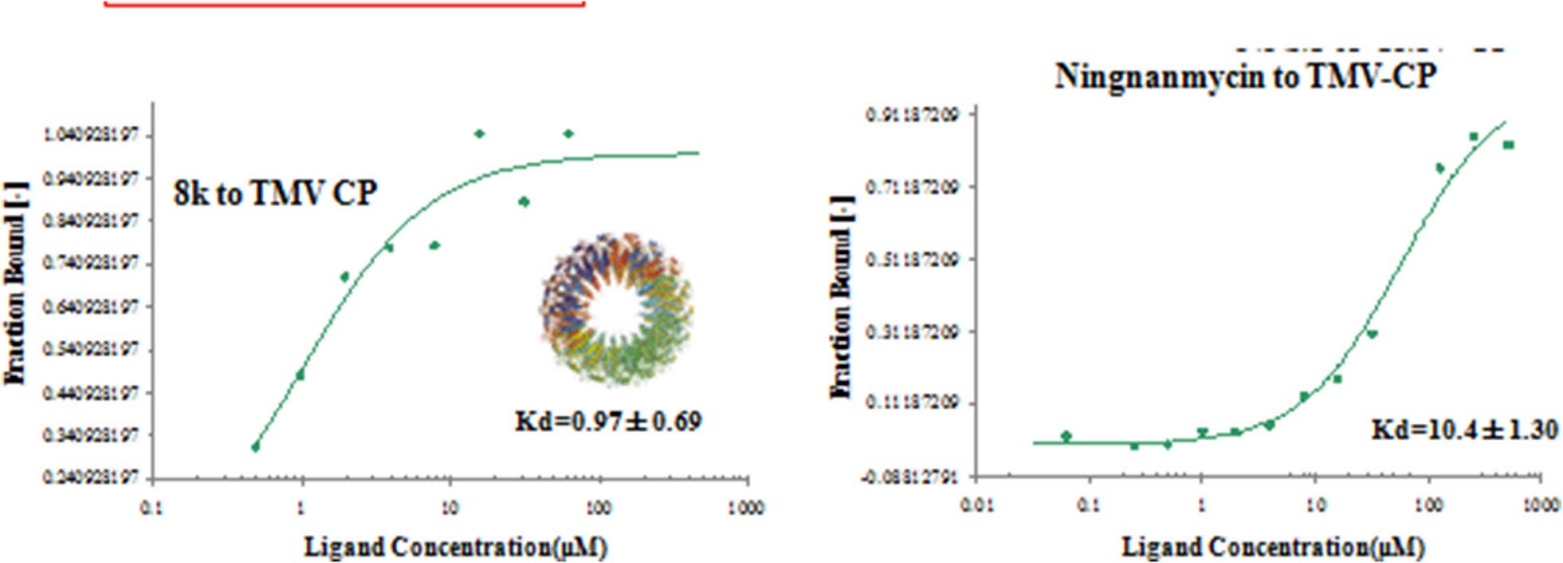
MST data analysis. Plot of the normalized fluorescence fraction bound vs. the concentration of TMV CP from MST experiments. Lines represent fits of the data points using the Kd equation. **8k** binds TMV CP with a Kd of 0.97 ± 0.69 μM in vitro. Ningnanmycin as a control

We studied the interactions between **8k** and TMV CP using Monolith NT. 115 (Nano Temper Technologies, Germany) [38, 39]. The results showed that the **8k** bound to TMV CP with a dissociation constant (Kd) of 0.97 ± 0.69 µM. An affinity between **8k** and TMV CP was far greater than the affinity between ningnanmycin (Control) and TMV CP. Base on anti-TMV activities and MST results. We speculate TMV CP protein was a potential target of **8k**. The affinity curves are shown in Fig. 2.

### Molecular docking of 8k and TMV CP

**Fig. 3:**
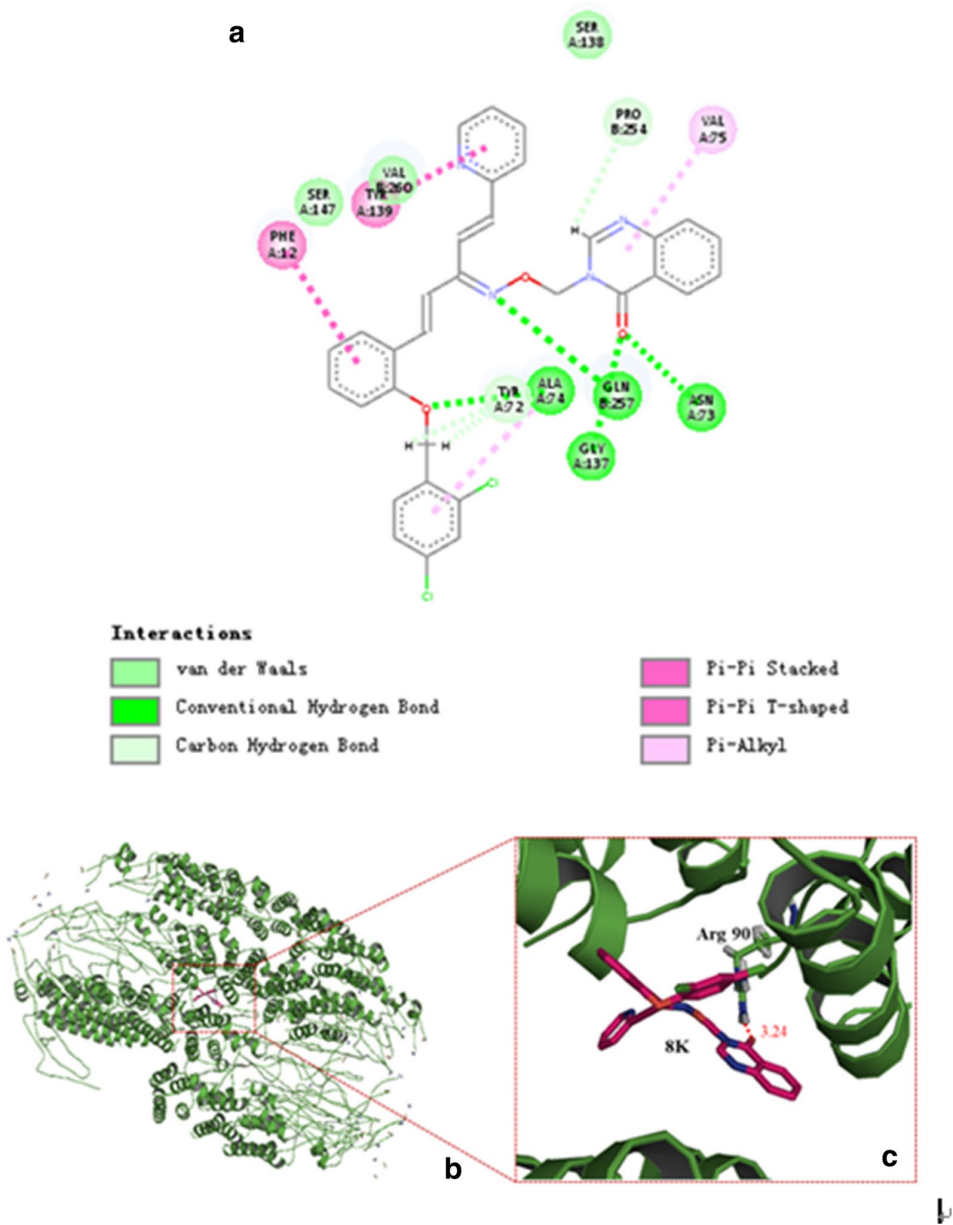
The 2D diagram **a** was drawn by Discovery Studio 4.5. Docking analysis of the interactions between **8k** and TMV CP. **b** The binding-sites between **8k** and TMV CP; **c** was a partial enlarged detail of (**b**), one binding-site, C=O–H–N = 3.24 Å, were found in the representative conformation. The stick model stands for **8k** and Arg90

To identify the **8k** recognition sites in TMV CP, we performed molecular docking using the Gold method with 200 cycles. After optimization, we found that Arg90 in the TMV CP shared one hydrogen bonds with **8k**, C=O–H–N = 3.24 Å (Fig. 3c). In our previously reported, Arg90 was an important residue for TMV RNA binding [38, 39]. These analyses indicated that Arg90 was the key residue to infect the activity of **8k**.

## Methods and materials

### Chemistry

**Scheme 1:**
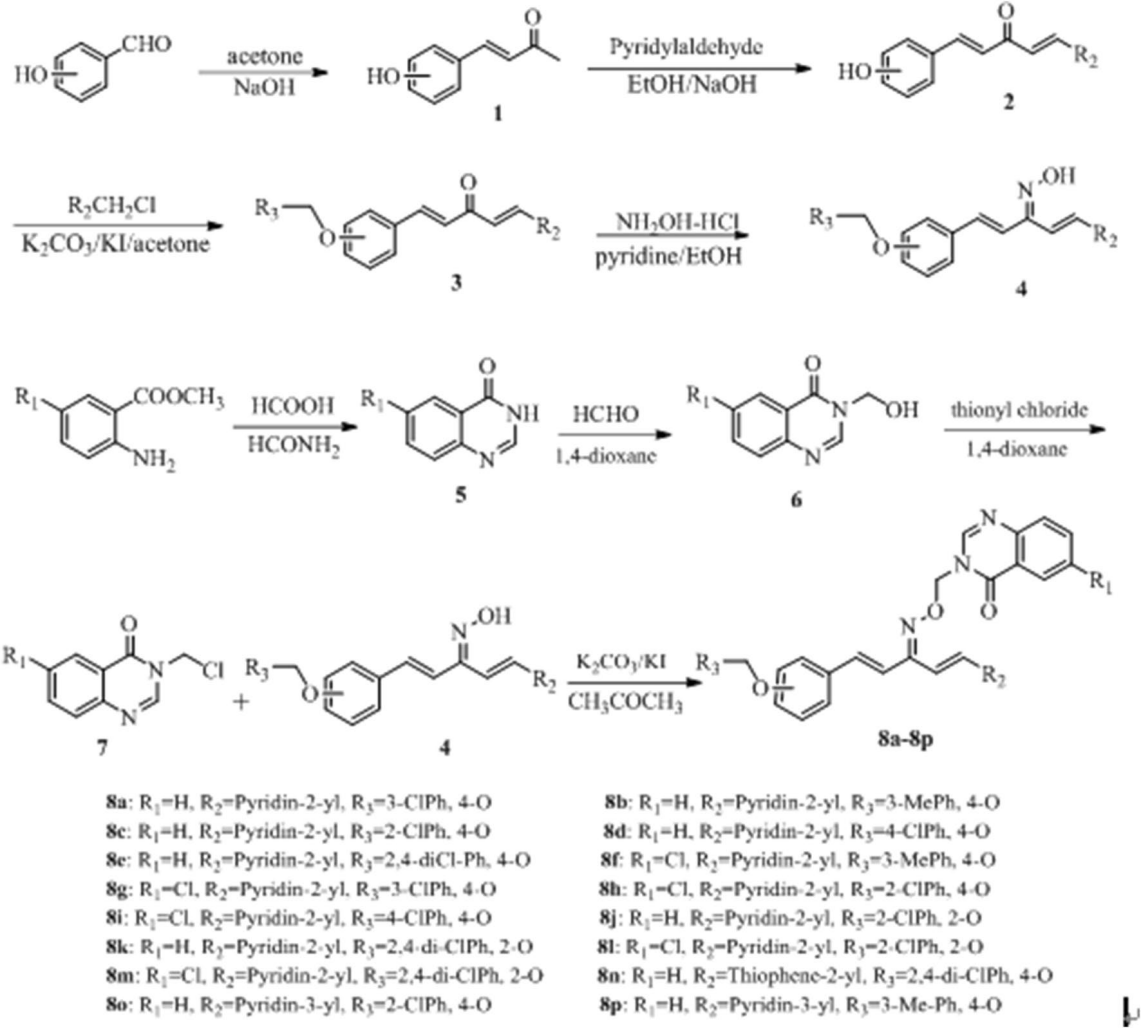
Synthetic route to title compounds **8a**–**8p**

Melting points of synthesized compounds were determined under an XT-4 binocular microscope (Beijing Tech Instrument Co., China) and were not corrected. The IR were recorded from KBr disks using a Bruker VECTOR 22 spectrometer (Bruker, USA). NMR spectra were obtained on a JEOL-ECX500 NMR spectrometer (JEOL, Japan) at room temperature using tetramethylsilane as an internal standard. Reaction was monitored by thin-layer chromatography (TLC) on silica gel GF_245_ (400 mesh). Mass spectral studies were performed on a quadrupole/electrostatic field orbitrap mass spectrometer (Thermo Scientific, USA). The micro thermophoresis of the compound and TMV CP was determined by a micro thermophoresis instrument (NanoTemper Tchnologies GmbH, Germany); the fluorescence spectroscopy of the compound interacting with TMV CP was determined by FluoroMax-4 fluorescence spectrometer (HORIBA Scientific, France). All reagents and reactants were purchased from commercial suppliers and were analytical grade or chemically pure. The synthetic route to penta-1,4-diene-3-one oxime ether derivatives containing a quinazolin-4(3*H*)-one scaffold was shown in Scheme 1. Intermediates **2** and **7** were prepared according to the reported methods [16, 31].

### Expression and purification of TMV CP

According to previous procedure described in the literature [36], The pET28a-TMV CP as a expression vector, which was stored at − 80 °C in our lab containing the full-length TMV CP gene. After overnight culture, the *Escherichia coli* strain *BL21*(*DE3*) containing the plasmid pET28a-TMV CP freshly transformed, and was transferred to 1 L Luria broth. At 37 °C in Luria–Bertani medium, the cells were grown which were supplemented with 50 μg/mL kanamycin and shaken at 200 rpm until the OD_600_ was 0.8. At 16 °C, using 0.8 mM IPTG inducing the protein expression overnight, then centrifugation to obtained the cells, and stored at − 80 °C. When analyzed, the cells were resuspended in lysis buffer (500 mM NaCl, 20 mM PB, 5 mM *β*-mercaptoethanol, 30 mM imidazole and 5% glycerol, pH 7.2) and then use sonication to lysed at 4 °C. By centrifugation at 12,000*g* for 30 min at 4 °C, the lysate was clarified, the soluble supernatants were loaded onto a 5 mL Ni–NTA column (GE Healthcare, USA), and using a linear gradient of 30–350 mM imidazole (pH 7.2) to eluting the protein. using a desalting column (GE Healthcare, USA) attached to an AKTA purifier protein liquid chromatography system (GE Healthcare, USA), the crude protein was performed at 4 °C, and the fractions containing target protein with His-tags were pooled, we use ultrafiltration (10 kDa cut-off) concentrated to a suitable concentration. Using a Genequant100 (GE Healthcare, USA) to determined the dealt protein concentration, stored at − 80 °C until further analysis.

### Interaction studies between **8k** and TMV CP

According to previously classic method, the binding was calculated for MST Monolith NT. 115 (Nano Temper Technologies, Germany) [38, 39]. Using a NT-647 dye (Nano Temper Technologies, Germany) incubating 0.5 µM of purified recombinant proteins with A range of ligands from 0 to 5 µM were for 5 min, and using the final concentration of 20 nM to the thermophoresis experiment. The selected compounds in DMSO were made a 16 point dilution series. Each compound dilution series was subsequently transferred to protein solutions (10 mM Tris–HCl and 100 mM sodium chloride pH 7.5, 0.05% Tween-20). The labeled TMV CP with each dilution point (1:1 mix) were incubation 15 min at room temperature, then the standard capillaries (NanoTemper Technologies, Germany) were filling into the samples. Under a setting of 20% LED and 40% IR laser, using the Monolith NT.115 microscale thermophoresis system (NanoTemper Technologies, Germany) to measure. Laser on time was set at 30 s, and laser-off time was set at 5 s. Using the mass action equation in the Nano Temper software, the Kd values were calculated from the duplicate reads, each experiments average of three replicates.

### General synthesis procedure for intermediate **4**

According to our previous synthesis procedure described in the literature [24], intermediates 4 were synthesized with minor modification. A solution of intermediate **3** (5.00 mmol), pyridine (13 mL) and hydroxylamine hydrochloride (1.73 mmol) in ethanol (25 mL) was stirred at room temperature for 13 h. Then, a white solid appeared in the reaction mixture was filtered and washed with ethanol (50 mL) to obtained intermediates **4**. Physical properties and related NMR data of intermediates **4** are listed in Additional file 1.

### General synthesis procedures for title compounds **8**

A solution of intermediates **4** (1.50 mmol), intermediates **7** (1.80 mmol) and K_2_CO_3_ (3.00 mmol) in acetonitrile (30 mL) was stirred under reflux for 4–5 h. Then, the mixture was filtered, the solvent was removed under vacuum, and the crude product was then separated by column chromatography with petroleum ether/ethyl acetate (*V:V *= 1:1) to obtain the title compounds **8a**–**8p**.

#### 3-(((((1E,3Z,4E)-1-(4-((3-chlorobenzyl)oxy)phenyl)-5-(pyridin-2-yl)penta-1,4-dien-3-ylidene)amino)oxy)methyl)quinazolin-4(3H)-one (**8a**)

White solid, m.p. 148–150 °C; yield: 36%; IR (KBr, cm^−1^): 3063, 2924, 1749, 1728, 1627, 1600, 1506, 1224, 1172, 1022, 958; ^1^H NMR (500 MHz, DMSO-*d*_*6*_) δ 8.56 (d, J = 4.0 Hz, 1H, Py-6-H), 8.54–8.51 (m, 1H, Qu-2-H), 8.16 (dd, J = 8.0, 1.3 Hz, 1H, Qu-5-H), 7.85–7.80 (m, 1H, Qu-7-H), 7.78 (td, J = 7.7, 1.8 Hz, 1H, Ar(4-O)-2-H), 7.66 (d, J = 8.0 Hz, 1H, Ar(4-O)-6-H), 7.61 (d, J = 7.8 Hz, 1H, Qu-8-H), 7.59 (s, 1H, Qu-6-H), 7.57 (s, 1H, Py-2-H), 7.54 (t, J = 7.4 Hz, 1H, Ar(3-Cl)-2-H), 7.49 (s, 1H, Py-4-H), 7.39 (d, J = 3.2 Hz, 1H, Py-5-H), 7.39 (s, 1H, Ar(3-Cl)-5-H), 7.37 (d, J = 2.2 Hz, 1H, Ar(3-Cl)-6-H), 7.35 (d, J = 4.1 Hz, 1H, Ar(4-O)-3-H), 7.29 (dd, J = 7.4, 5.3 Hz, 1H, Ar(4-O)-5-H), 7.20 (dd, J = 18.6, 13.6 Hz, 2H, Ar–CH=), 7.10–7.05 (m, 1H, Py–C=CH), 7.03 (s, 1H, Py–CH=), 7.01 (s, 1H, Ar–C =CH), 5.99 (s, 2H, CH_2_), 5.14 (s, 2H, CH_2_); ^13^C NMR (125 MHz, DMSO-*d*_*6*_) δ 160.5, 159.7, 155.8, 154.3, 150.2, 148.6, 148.3, 139.9, 138.7, 137.5, 135.5, 135.1, 133.7, 130.9, 129.8, 129.0, 128.4, 127.9, 127.9, 126.9, 126.8, 125.3, 124.0, 123.8, 122.1, 115.7, 114.4, 77.8, 68.9; HRMS calcd for C_32_H_25_ClN_4_O_3_ [M+H]^+^: 549.1688, found 549.1683.

#### 3-(((((1E,3Z,4E)-1-(4-((3-methylbenzyl)oxy)phenyl)-5-(pyridin-2-yl)penta-1,4-dien-3-ylidene)amino)oxy)methyl)quinazolin-4(3H)-one (**8b**)

White solid, m.p. 155–157 °C; yield: 41%; IR (KBr, cm^−1^): 2931, 1747, 1732, 1681, 1593, 1506, 1247, 1172, 1091, 1012, 748; ^1^H NMR (500 MHz, CDCl_3_) δ 8.61 (d, J = 4.5 Hz, 1H, Py-6-H), 8.39 (s, 1H, Qu-2-H), 8.34 (d, J = 7.9 Hz, 1H, Qu-5-H), 7.78–7.73 (m, 1H, Qu-7-H), 7.72–7.65 (m, 2H, Ar(4-O)-2,6-2H), 7.52 (dd, J = 13.4, 7.4 Hz, 2H, Ar(2-Cl)-3-H, Qu-8-H), 7.47 (d, J = 8.7 Hz, 3H, Qu-6-H, Py-3,4-2H), 7.41 (d, J = 6.1 Hz, 1H, Py-5-H), 7.38 (s, 1H, Ar(2-Cl)-6-H), 7.36 (d, J = 7.9 Hz, 1H, Ar(2-Cl)-4-H), 7.28 (d, J = 6.2 Hz, 1H, Ar(2-Cl)-5-H), 7.19 (t, J = 6.0 Hz, 2H, Ar(4-O)-3,5-2H), 7.16 (s, 1H, Py–CH=), 7.09 (d, J = 16.6 Hz, 1H, Py–C=CH), 6.96 (d, J = 8.5 Hz, 2H, Ar–CH=, Ar–C=CH), 6.02 (s, 2H, CH_2_), 5.18 (s, 2H, CH_2_); ^13^C NMR (125 MHz, CDCl_3_) δ 161.0, 159.6, 156.2, 154.4, 149.9, 148.2, 146.9, 138.3, 136.8, 134.7, 134.4, 132.7, 129.5, 129.3, 129.2, 129.1, 128.9, 127.7, 127.4, 127.2, 127.1, 125.2, 123.3, 123.1, 122.3, 115.2, 114.8, 77.5, 67.2; HRMS calcd for C_32_H_25_ClN_4_O_3_ [M+H]^+^: 529.2234, found 529.2233.

#### 3-(((((1E,3Z,4E)-1-(4-((2-chlorobenzyl)oxy)phenyl)-5-(pyridin-2-yl)penta-1,4-dien-3-ylidene)amino)oxy)methyl)quinazolin-4(3H)-one (**8c**)

White solid, m.p. 135–137 °C; yield: 33%; IR (KBr, cm^−1^): 3061, 2927, 1732, 1716, 1600, 1506, 1454, 1284, 1234, 1074, 1028, 752; ^1^H NMR (500 MHz, CDCl_3_) δ 8.62 (s, 1H, Py-6-H), 8.39 (d, J = 3.3 Hz, 1H, Qu-2-H), 8.35 (dd, J = 7.9, 1.9 Hz, 1H, Qu-5-H), 7.75 (dd, J = 10.9, 4.0 Hz, 1H, Qu-7-H), 7.73–7.69 (m, 1H, Ar(4-O)-2-H), 7.67 (dd, J = 6.7, 2.5 Hz, 1H, Ar(4-O)-6-H), 7.53–7.48 (m, 1H, Qu-8-H), 7.47 (d, J = 3.0 Hz, 1H, Qu-6-H), 7.45 (d, J = 3.1 Hz, 1H, Py-3-H), 7.40 (dd, J = 15.9, 3.4 Hz, 1H, Ar(3-CH_3_)-5-H), 7.36 (d, J = 7.7 Hz, 1H, Py-5-H), 7.28 (dd, J = 7.4, 3.3 Hz, 1H, Py-4-H), 7.25 (d, J = 3.6 Hz, 2H, Ar(3-CH_3_)-4,6-2H), 7.22 (d, J = 8.7 Hz, 1H, Ar(3-CH_3_)-2-H), 7.20 (d, J = 1.8 Hz, 1H, Ar(4-O)-3-H), 7.16 (d, J = 3.3 Hz, 1H, Ar(4-O)-5-H), 7.10 (dd, J = 17.2, 14.2 Hz, 2H, Ar–CH=, Py–C=CH), 6.96 (d, J = 3.1 Hz, 1H, Ar–C=CH), 6.94 (d, J = 3.1 Hz, 1H, Py–CH=), 6.02 (d, J = 3.1 Hz, 2H, CH_2_), 5.04 (d, J = 2.6 Hz, 2H, CH_2_), 2.37 (d, J = 2.7 Hz, 3H, CH_3_); ^13^C NMR (125 MHz, CDCl_3_) δ 161.0, 160.0, 156.2, 154.4, 149.9, 148.2, 146.9, 138.5, 138.3, 136.8, 136.6, 134.7, 134.6, 129.2, 129.0, 128.8, 128.6, 128.4, 127.7, 127.4, 127.2, 125.1, 124.7, 123.3, 123.2, 122.3, 115.2, 114.6, 77.5, 70.2, 21.5; HRMS calcd for C_33_H_28_N_4_O_3_ [M+H]^+^: 549.1687, found 549.1696.

#### 3-(((((1E,3Z,4E)-1-(4-((4-chlorobenzyl)oxy)phenyl)-5-(pyridin-2-yl)penta-1,4-dien-3-ylidene)amino)oxy)methyl)quinazolin-4(3H)-one (**8d**)

White solid, m.p. 147–149 °C; yield: 27%; IR (KBr, cm^−1^): 3061, 2941, 1747, 1732, 1614, 1487, 1454, 1282, 1232, 1107, 1028, 916, 748; ^1^H NMR (500 MHz, CDCl_3_) δ 8.61 (d, J = 4.2 Hz, 1H, Py-6-H), 8.39 (s, 1H, Qu-2-H), 8.34 (d, J = 7.1 Hz, 1H, Qu-5-H), 7.75 (dd, J = 11.0, 4.3 Hz, 1H, Qu-7-H), 7.71 (s, 1H, Ar(4-O)-2-H), 7.70–7.68 (m, 1H, Ar(4-O)-6-H), 7.68–7.65 (m, 1H, Qu-8-H), 7.50 (t, J = 7.5 Hz, 1H, Qu-6-H), 7.46 (d, J = 8.7 Hz, 2H, Py-3,4-2H), 7.40 (d, J = 15.8 Hz, 1H, Py-5-H), 7.37 (s, 1H, Ar(4-Cl)-3-H), 7.25 (s, 1H, Ar(4-Cl)-5-H), 7.21 (d, J = 4.8 Hz, 1H, Ar(4-Cl)-2-H), 7.19 (s, 1H, Ar(4-Cl)-6-H), 7.19–7.18 (m, 1H, Py–C=CH), 7.16 (s, 1H, Ar(4-O)-3-H), 7.16 (s, 1H, Ar(4-O)-5-H), 7.08 (d, J = 16.6 Hz, 1H, Ar–CH=), 6.93 (d, J = 8.7 Hz, 2H, Py–CH=, Ar–C=CH), 6.02 (s, 2H, CH_2_), 5.02 (d, J = 16.2 Hz, 2H, CH_2_); ^13^C NMR (125 MHz, CDCl_3_) δ 161.1, 159.6, 156.1, 154.4, 149.9, 148.2, 146.9, 138.2, 136.8, 135.2, 134.7, 134.0, 129.3, 129.1, 128.9, 128.9, 127.7, 127.4, 127.2, 125.1, 123.3, 123.2, 122.3, 115.2, 114.8, 77.5, 69.3; HRMS calcd for C_32_H_25_ClN_4_O_3_ [M+H]^+^: 549.1687, found 549.1691.

#### 3-(((((1E,3Z,4E)-1-(4-((2,4-dichlorobenzyl)oxy)phenyl)-5-(pyridin-2-yl)penta-1,4-dien-3-ylidene)amino)oxy)methyl)quinazolin-4(3H)-one (**8e**)

White solid, m.p. 174–176 °C; yield: 24%; IR (KBr, cm^−1^): 3065, 2871, 1734, 1716, 1602, 1521, 1489, 1456, 1344, 1249, 1172, 1062, 1012, 932, 752; ^1^H NMR (500 MHz, CDCl_3_) δ 8.61 (d, *J* = 4.1 Hz, 1H, Py-6-H), 8.39 (s, 1H, Qu-2-H), 8.34 (dd, *J* = 8.0, 1.1 Hz, 1H, Qu-5-H), 7.78–7.73 (m, 1H, Qu-7-H), 7.72–7.65 (m, 2H, Ar(4-O)-2,6-2H), 7.52–7.49 (m, 1H, Ar(2,4-2Cl)-3-H), 7.49–7.45 (m, 3H, Qu-6,8-2H, Py-3-H), 7.41 (t, *J* = 9.1 Hz, 2H, Py-4,5-2H), 7.36 (d, *J* = 7.9 Hz, 1H, Ar(2,4-2Cl)-5-H), 7.27 (dd, *J* = 8.3, 2.0 Hz, 1H, Ar(2,4-2Cl)-6-H), 7.22–7.15 (m, 3H, Py–CH=, Ar(4-O)-3,5-2H), 7.08 (d, *J* = 16.7 Hz, 1H, Ar–C=CH), 6.95 (t, *J* = 5.7 Hz, 2H, Ar–CH=, Py–C=CH), 6.02 (s, 2H, CH_2_), 5.13 (s, 2H, CH_2_); ^13^C NMR (125 MHz, CDCl_3_) δ 161.0, 159.3, 156.1, 154.4, 149.9, 148.2, 146.9, 138.1, 136.8, 134.7, 134.3, 133.3, 133.1, 129.7, 129.4, 129.3, 127.7, 127.4, 127.2, 125.1, 123.3, 123.2, 122.3, 115.2, 115.0, 77.5, 66.7. HRMS calcd for C_32_H_25_ClN_4_O_3_ [M+H]^+^: 583.1298, found 583.1297.

#### 6-chloro-3-(((((1E,3Z,4E)-1-(4-((3-methylbenzyl)oxy)phenyl)-5-(pyridin-2-yl)penta-1,4-dien-3-ylidene)amino)oxy)methyl)quinazolin-4(3H)-one (**8f**)

White solid, m.p. 168–170 °C; yield: 34%; IR (KBr, cm^−1^): 1741, 1600, 1506, 1247, 1172, 1089, 1066, 1012, 960, 821, 748; ^1^H NMR (500 MHz, CD_3_COCD_3_) δ 8.61 (d, J = 4.6 Hz, 1H, Py-6-H), 8.37 (s, 1H, Qu-2-H), 8.30 (d, J = 2.3 Hz, 1H, Qu-5-H), 7.70–7.63 (m, 3H, Ar(4-O)-2,6-2H, Qu-7-H), 7.46 (t, J = 5.7 Hz, 2H, Ar(3-CH_3_)-5-H, Py-3-H), 7.39 (d, J = 15.7 Hz, 1H, Py-4-H), 7.35 (d, J = 7.9 Hz, 1H, Py-5-H), 7.28 (d, J = 7.5 Hz, 1H, Ar(3-CH_3_)-6-H), 7.24 (s, 1H, Qu-8-H), 7.21 (dd, J = 7.8, 4.1 Hz, 2H, Py–C=CH, Ar(3-CH_3_)-4-H), 7.19–7.12 (m, 3H, Ar(3-CH_3_)-2-H, Ar(4-O)-3,5-2H), 7.08 (d, J = 16.6 Hz, 1H, Ar–CH=), 6.95 (d, J = 8.8 Hz, 2H, Py–CH=, Ar–C=CH), 6.00 (s, 2H, CH_2_), 5.04 (s, 2H, CH_2_), 2.37 (s, 3H, CH_3_); ^13^C NMR (125 MHz, CD_3_COCD_3_) δ 160.1, 160.0, 156.4, 154.4, 149.9, 147.0, 146.7, 138.5, 138.5, 136.8, 136.6, 135.1, 134.8, 133.3, 129.4, 129.2, 129.0, 128.7, 128.6, 128.3, 126.6, 125.1, 124.7, 123.4, 123.3, 123.2, 115.2, 114.5, 77.6, 70.2, 21.5; HRMS calcd for C_33_H_27_ClN_4_O_3_ [M+H]^+^: 563.1844, found 563.1844.

#### 6-Chloro-3-(((((1E,3Z,4E)-1-(4-((3-chlorobenzyl)oxy)phenyl)-5-(pyridin-2-yl)penta-1,4-dien-3-ylidene)amino)oxy)methyl)quinazolin-4(3H)-one (**8g**)

White solid, m.p. 126–128 °C; yield: 26%; IR (KBr, cm^−1^): 3050, 2954, 1734, 1610, 1508, 1487, 1456, 1284, 1234, 1174, 1109, 1029, 916, 823, 752; ^1^H NMR (500 MHz, CDCl_3_) δ 8.61 (d, J = 3.9 Hz, 1H, Py-6-H), 8.37 (s, 1H, Qu-2-H), 8.29 (d, J = 2.3 Hz, 1H, Qu-5-H), 7.70–7.67 (m, 2H, Ar(4-O)-2,6-2H), 7.65 (dd, J = 9.8, 5.3 Hz, 1H, Qu-7-H), 7.46 (d, J = 8.8 Hz, 2H, Py-3-H, Ar(3-Cl)-2-H), 7.43 (s, 1H, Py-4-H), 7.39 (d, J = 15.9 Hz, 1H, Py-5-H), 7.35 (d, J = 7.8 Hz, 1H, Ar(3-Cl)-5-H), 7.30 (t, J = 2.5 Hz, 3H, Ar(3-Cl)-4,6-2H, Qu-8-H), 7.22–7.19 (m, 1H, Py–C=CH), 7.16 (dd, J = 16.2, 4.4 Hz, 2H, Ar(4-O)-3,5-2H), 7.08 (d, J = 16.7 Hz, 1H, Ar–CH=), 6.96–6.91 (m, 2H, Ar–C=CH, Py–CH=), 6.00 (s, 2H, CH_2_), 5.05 (s, 2H, CH_2_); ^13^C NMR (125 MHz, CDCl_3_) δ 160.0, 159.6, 156.4, 154.4, 149.9, 147.0, 146.7, 138.8, 138.4, 136.8, 135.1, 134.9, 134.7, 133.3, 130.0, 129.4, 129.3, 129.0, 128.3, 127.5, 126.6, 125.5, 125.1, 123.4, 123.3, 123.2, 115.2, 114.7, 77.6, 69.3; HRMS calcd for C_32_H_25_Cl_2_N_4_O_3_ [M+H]^+^: 583.1298, found 583.1298.

#### 6-Chloro-3-(((((1E,3Z,4E)-1-(4-((2-chlorobenzyl)oxy)phenyl)-5-(pyridin-2-yl)penta-1,4-dien-3-ylidene)amino)oxy)methyl)quinazolin-4(3H)-one (**8h**)

White solid, m.p. 214–216 °C; yield: 21%; IR (KBr, cm^−1^): 2922, 2852, 1747, 1597, 1521, 1452, 1346, 1244, 1058, 923, 837, 736, 700; ^1^H NMR (500 MHz, CDCl_3_) δ 8.62 (d, *J* = 3.7 Hz, 1H, Py-6-H), 8.37 (s, 1H, Qu-2-H), 8.30 (d, *J* = 2.3 Hz, 1H, Qu-5-H), 7.70–7.63 (m, 3H, Ar(4-O)-2,6-2H, Ar(2-Cl)-3-H), 7.53 (dd, *J* = 7.1, 2.1 Hz, 1H, Qu-7-H), 7.47 (d, *J* = 8.7 Hz, 2H, Py-3,4-2H), 7.42–7.37 (m, 2H, Py-5-H, Ar(2-Cl)-6-H), 7.35 (d, *J* = 7.8 Hz, 1H, Qu-8-H), 7.31–7.26 (m, 2H, Ar(2-Cl)-4,5-2H), 7.21 (dd, *J* = 6.2, 1.9 Hz, 1H, Py–C=CH), 7.17 (dd, *J* = 16.3, 3.6 Hz, 2H, Ar(4-O)-3,5-2H), 7.09 (d, *J* = 16.6 Hz, 1H, Ar–CH=), 6.97 (d, *J* = 8.7 Hz, 2H, Ar–C=CH, Py–CH=), 6.00 (s, 2H, CH_2_), 5.19 (s, 2H, CH_2_); ^13^C NMR (125 MHz, CDCl_3_) δ 160.0, 159.7, 156.4, 154.4, 149.9, 147.0, 146.7, 138.4, 136.8, 135.1, 134.9, 134.4, 133.3, 132.7, 129.5, 129.4, 129.3, 129.2, 129.0, 128.9, 127.1, 126.6, 125.1, 123.4, 123.3, 123.2, 115.2, 114.7, 77.6, 67.3; HRMS calcd for C_32_H_24_Cl_2_N_4_O_3_ [M+H]^+^: 583.1298, found 583.1306.

#### 6-Chloro-3-(((((1E,3Z,4E)-1-(4-((4-chlorobenzyl)oxy)phenyl)-5-(pyridin-2-yl)penta-1,4-dien-3-ylidene)amino)oxy)methyl)quinazolin-4(3H)-one (**8i**)

White solid, m.p. 172-–174 °C; yield: 18%; IR (KBr, cm^−1^): 1718, 1633, 1598, 1568, 1448, 1238, 1170, 1064, 1012, 914, 823, 713; ^1^H NMR (500 MHz, CDCl_3_) δ 8.61 (d, J = 4.0 Hz, 1H, Py-6-H), 8.37 (s, 1H, Qu-2-H), 8.29 (d, J = 2.3 Hz, 1H, Qu-5-H), 7.69 (dd, J = 5.0, 2.8 Hz, 1H, Ar(4-O)-2-H), 7.67 (d, J = 2.4 Hz, 1H, Ar(4-O)-6-H), 7.65 (dd, J = 10.3, 5.2 Hz, 1H, Qu-7-H), 7.48–7.44 (m, 2H, Py-3,4-2H), 7.41–7.37 (m, 1H, Py-5-H), 7.37–7.34 (m, 5H, Ar(4-Cl)-2,3,4,5-4H, Qu-8-H), 7.22–7.16 (m, 2H, Ar(4-O)-3,5-2H), 7.15 (d, J = 5.5 Hz, 1H, Ar–CH=), 7.08 (d, J = 16.7 Hz, 1H, Py–C=CH), 6.95–6.91 (m, 2H, Ar–C=CH, Py–CH=), 6.00 (s, 2H, CH_2_), 5.04 (s, 2H, CH_2_); ^13^C NMR (125 MHz, CDCl_3_) δ 160.0, 159.7, 156.4, 154.4, 149.9, 147.0, 146.7, 138.4, 136.8, 135.2, 135.1, 134.8, 134.0, 133.3, 129.4, 129.3, 129.0, 128.9, 128.8, 126.6, 125.1, 123.4, 123.3, 123.2, 115.2, 114.7, 77.6, 69.3; HRMS calcd for C_32_H_25_Cl_2_N_4_O_3_ [M+H]^+^: 583.1225, found 583.1359.

#### 3-(((((1E,3Z,4E)-1-(2-(2-chlorophenoxy)phenyl)-5-(pyridin-2-yl)penta-1,4-dien-3-ylidene)amino)oxy)methyl)quinazolin-4(3H)-one (**8j**)

White solid, m.p. 95–97 °C; yield: 36%; IR (KBr, cm^−1^): 2924, 1734, 1716, 1583, 1558, 1489, 1247, 1172, 1076, 1012, 748; ^1^H NMR (500 MHz, CDCl_3_) δ 8.60 (d, J = 4.4 Hz, 1H, Py-6-H), 8.38 (s, 1H, Qu-2-H), 8.34 (d, J = 8.0 Hz, 1H, Qu-5-H), 7.75 (dd, J = 11.0, 4.1 Hz, 1H, Qu-7-H), 7.70 (d, J = 8.0 Hz, 1H, Ar(2-Cl)-3-H), 7.67–7.60 (m, 3H, Ar(2-O)-6-H, Qu-6,8-2H), 7.52–7.46 (m, 2H, Py-3,4-2H), 7.43 (d, J = 15.9 Hz, 1H, Py-5-H), 7.32 (d, J = 8.8 Hz, 1H, Ar(2-Cl)-6-H), 7.31–7.24 (m, 3H, Ar(2-Cl)-4,5-2H, Ar(2-O)-4-H), 7.22–7.14 (m, 3H, Py–C=CH, Ar–CH=, Ar(2-O)-5-H), 7.08 (t, J = 7.5 Hz, 1H, Ar–C=CH), 7.00 (t, J = 7.5 Hz, 1H, Ar(2-O)-3-H), 6.93 (d, J = 8.2 Hz, 1H, Py–CH=), 6.01 (s, 2H, CH_2_), 5.19 (s, 2H, CH_2_); ^13^C NMR (125 MHz, CDCl_3_) δ 161.0, 156.8, 156.5, 154.6, 149.9, 148.2, 146.9, 136.6, 135.1, 134.7, 134.5, 133.7, 132.5, 130.7, 129.4, 128.9, 128.6, 127.7, 127.7, 127.4, 127.2, 127.1, 125.6, 125.4, 123.1, 123.0, 122.3, 121.5, 117.1, 112.7, 77.5, 67.6; HRMS calcd for C_32_H_25_ClN_4_O_3_ [M+H]^+^: 549.1687, found 549.1684.

#### 3-(((((1E,3Z,4E)-1-(2-(2,4-dichlorophenoxy)phenyl)-5-(pyridin-2-yl)penta-1,4-dien-3-ylidene)amino)oxy)methyl)quinazolin-4(3H)-one (**8k**)

White solid, m.p. 194–196 °C; yield: 44%; IR (KBr, cm^−1^): 3042, 2923, 1734, 1608, 1508, 1454, 1282, 1234, 1172, 1105, 1031, 918, 748; ^1^H NMR (500 MHz, CDCl_3_) δ 8.63–8.59 (m, 1H, Py-6-H), 8.38 (s, 1H, Qu-2-H), 8.34 (dd, J = 8.0, 1.2 Hz, 1H, Qu-5-H), 7.76 (m, 1H, Qu-7-H), 7.72–7.69 (m, 1H, Ar(2-O)-6-H), 7.69–7.63 (m, 2H, Ar(2,4-2Cl)-3-H, Qu-8-H), 7.61–7.56 (m, 1H, Qu-6-H), 7.50 (ddd, J = 8.1, 7.1, 1.2 Hz, 1H, Py-3-H), 7.45–7.39 (m, 2H, Py-4,5-2H), 7.35 (d, J = 2.1 Hz, 1H, Ar(2-O)-4-H), 7.29 (dd, J = 12.4, 4.4 Hz, 3H, Ar(2,4-2Cl)-5,6-2H, Ar–CH=), 7.22 (m, 1H, Ar(2-O)-5-H), 7.18 (d, J = 15.9 Hz, 1H, Py–C=CH), 7.05 (dd, J = 8.3, 2.1 Hz, 1H, Ar–C=CH), 7.01 (t, J = 7.5 Hz, 1H, Ar(2-O)-3-H), 6.91 (d, J = 8.2 Hz, 1H, Py–CH=), 6.02 (s, 2H, CH_2_), 5.14 (s, 2H, CH_2_); ^13^C NMR (125 MHz, CDCl_3_) δ 161.0, 156.7, 156.2, 154.5, 149.9, 148.2, 146.9, 136.7, 135.2, 134.7, 134.1, 133.5, 133.2, 133.1, 130.7, 129.6, 129.2, 127.7, 127.7, 127.5, 127.4, 127.2, 125.5, 125.4, 123.2, 123.1, 122.3, 121.7, 117.1, 112.7, 77.5, 67.1; HRMS calcd for C_32_H_25_Cl_2_N_4_O_3_ [M+H]^+^: 583.1298, found 583.1300.

#### 6-Chloro-3-(((((1E,3Z,4E)-1-(2-(2-chlorophenoxy)phenyl)-5-(pyridin-2-yl)penta-1,4-dien-3-ylidene)amino)oxy)methyl)quinazolin-4(3H)-one (**8l**)

White solid, m.p. 153–155 °C; yield: 23%; IR (KBr, cm^−1^): 2940, 2830, 1734, 1716, 1600, 1519, 1489, 1456, 1346, 1284, 1259, 1107, 1083, 1029, 802, 736; ^1^H NMR (500 MHz, CDCl_3_) δ 8.61 (d, J = 4.4 Hz, 1H, Py-6-H), 8.36 (s, 1H, Qu-2-H), 8.29 (d, J = 2.2 Hz, 1H, Qu-5-H), 7.67 (dt, J = 4.5, 2.2 Hz, 1H, Ar(2-Cl)-3-H), 7.65 (s, 2H, Ar(2-O)-6-H, Qu-7-H), 7.63 (s, 1H, Py-3-H), 7.60 (s, 1H, Py-4-H), 7.48 (d, J = 7.5 Hz, 1H, Py-5-H), 7.42 (d, J = 15.9 Hz, 1H, Ar(2-Cl)-6-H), 7.33 (d, J = 7.9 Hz, 1H, Qu-8-H), 7.30 (dd, J = 6.0, 3.5 Hz, 2H, Ar(2-Cl)-5-H, Ar(2-Cl)-4-H), 7.27 (d, J = 3.6 Hz, 1H, Ar(2-O)-4-H), 7.21–7.15 (m, 3H, Ar–CH=, Ar(2-O)-5-H, Ar(2-O)-3-H), 7.08 (t, J = 7.3 Hz, 1H, Py–C=CH), 7.00 (t, J = 7.5 Hz, 1H, Ar–C=CH), 6.94 (d, J = 8.3 Hz, 1H, Py–CH=), 5.99 (s, 2H, CH_2_), 5.19 (s, 2H, CH_2_); ^13^C NMR (125 MHz, CDCl_3_) δ 160.0, 157.0, 156.5, 154.5, 149.9, 147.0, 146.7, 136.7, 135.3, 135.1, 134.5, 133.9, 133.3, 132.5, 130.8, 129.4, 129.3, 129.0, 128.6, 127.7, 127.1, 126.6, 125.5, 125.3, 123.4, 123.1, 123.1, 121.5, 117.0, 112.8, 77.5, 67.7; HRMS calcd for C_32_H_25_Cl_2_N_4_O_3_ [M+H]^+^: 583.1298, found 583.1285.

#### 6-Chloro-3-(((((1E,3Z,4E)-1-(2-(2,4-dichlorophenoxy)phenyl)-5-(pyridin-2-yl)penta-1,4-dien-3-ylidene)amino)oxy)methyl)quinazolin-4(3H)-one (**8m**)

White solid, m.p. 113–115 °C; yield: 32%; IR (KBr, cm^−1^): 2927, 1747, 1519, 1456, 1346, 1261, 1091, 1076, 1029, 800; ^1^H NMR (500 MHz, CDCl_3_) δ 8.61 (d, *J* = 3.9 Hz, 1H, Py-6-H), 8.37 (d, *J* = 6.1 Hz, 1H, Qu-2-H), 8.29 (t, *J* = 2.3 Hz, 1H, Qu-5-H), 7.68 (m, 2H, Ar(2,4-2Cl)-3-H, Ar(2-O)-6-H), 7.65 (d, *J* = 8.6 Hz, 1H, Qu-7-H), 7.47 (dt, *J* = 4.8, 2.1 Hz, 3H, Py-3,4,5-3H), 7.40 (dd, *J* = 14.7, 9.0 Hz, 2H, Ar(2,4-2Cl)-5,6-2H), 7.36 (d, *J* = 7.8 Hz, 1H, Qu-8-H), 7.27 (dd, *J* = 8.3, 2.1 Hz, 1H, Ar(2-O)-4-H), 7.22–7.20 (m, 1H, Ar–CH=), 7.19 (t, *J* = 2.5 Hz, 1H, Ar(2-O)-5-H), 7.15 (d, *J* = 3.7 Hz, 1H, Ar(2-O)-3-H), 7.09 (d, *J* = 16.7 Hz, 1H, Py–C=CH), 6.97–6.93 (m, 2H, Py–CH=, Ar–C=CH), 6.00 (s, 2H, CH_2_), 5.13 (s, 2H, CH_2_); ^13^C NMR (125 MHz, CDCl_3_) δ 160.0, 159.4, 156.3, 154.4, 149.9, 147.0, 146.7, 138.3, 136.8, 135.1, 134.9, 134.4, 133.3, 133.3, 133.1, 129.7, 129.4, 129.3, 129.2, 127.5, 126.6, 125.1, 123.4, 123.3, 123.2, 115.2, 114.9, 77.6, 66.7; HRMS calcd for C_32_H_24_Cl_3_N_4_O_3_ [M+H]^+^: 617.0908, found 617.0909.

#### 3-(((((1E,3Z,4E)-1-(4-((2,4-dichlorobenzyl)oxy)phenyl)-5-(thiophen-2-yl)penta-1,4-dien-3-ylidene)amino)oxy)methyl)quinazolin-4(3H)-one (**8n**)

White solid, m.p. 84–86 °C; yield: 23%; IR (KBr, cm^−1^): 3062, 2944, 1716, 1600, 1558, 1503, 1456, 1284, 1228, 1174, 750; ^1^H NMR (500 MHz, CD_3_COCD_3_) δ 8.46 (s, 1H, Qu-2-H), 8.22 (dd, J = 8.0, 1.4 Hz, 1H, Qu-5-H), 7.82–7.77 (m, 1H, Qu-7-H), 7.64 (dd, J = 14.9, 8.2 Hz, 2H, Ar(2,4-2Cl)-3-H, Qu-8-H), 7.60–7.57 (m, 2H, Ar(4-O)-2,6-2H), 7.53 (dd, J = 13.4, 4.5 Hz, 2H, Qu-6-H, Thiophene-5-H), 7.45–7.36 (m, 3H, Ar(2,4-2Cl)-5,6-2H, Thiophene-3-H), 7.25 (d, J = 3.4 Hz, 1H, Thiophene-4-H), 7.19 (q, J = 16.8 Hz, 2H, Ar(4-O)-3,5-2H), 7.09–7.02 (m, 3H, Ar–CH=, Thiophene–CH=, Thiophene–C=CH), 6.72 (d, J = 16.0 Hz, 1H, Ar–C=CH), 6.04 (s, 2H, CH_2_), 5.20 (s, 2H, CH_2_); ^13^C NMR (125 MHz, CD_3_COCD_3_) δ 161.0, 159.3, 156.4, 148.2, 146.9, 141.6, 137.9, 134.8, 134.4, 133.3, 133.1, 129.7, 129.4, 129.2, 129.0, 128.3, 127.9, 127.7, 127.5, 127.2, 126.2, 122.3, 120.5, 115.2, 115.2, 77.2, 66.7; HRMS calcd for C_31_H_23_Cl_2_N_3_O_3_S [M+H]^+^: 588.0909, found 588.0907.

#### 3-(((((1E,3Z,4E)-1-(4-((2-chlorobenzyl)oxy)phenyl)-5-(pyridin-3-yl)penta-1,4-dien-3-ylidene)amino)oxy)methyl)quinazolin-4(3H)-one (**8o**)

White solid, m.p. 94–96 °C; yield: 25%; IR (KBr, cm^−1^): 2924, 1741, 1683, 1591, 1282, 1251, 1091, 1078, 1014, 918, 852, 761; ^1^H NMR (500 MHz, CDCl_3_) δ 8.83 (s, 1H, Py-2-H), 8.63 (d, J = 4.6 Hz, 1H, Py-6-H), 8.57 (s, 1H, Qu-2-H), 8.39 (d, J = 7.9 Hz, 1H, Qu-5-H), 8.07 (d, J = 7.9 Hz, 1H, Py-4-H), 8.00 (s, 1H, Qu-7-H), 7.92 (t, J = 7.5 Hz, 1H, Ar(4-O)-2-H), 7.81 (d, J = 8.1 Hz, 1H, Ar(4-O)-6-H), 7.70–7.61 (m, 5H, Ar(2-Cl)-3,6-2H, Qu-6,8-2H, Py-5-H), 7.57–7.52 (m, 1H, Ar(2-Cl)-4-H), 7.49–7.42 (m, 3H, Ar(2-Cl)-5-H, Ar(4-O)-3,5-2H), 7.29 (s, 1H, Py–C=CH), 7.17–7.09 (m, 3H, Ar–CH=, Py–CH=, Ar–C=CH), 6.15 (s, 2H, CH_2_), 5.31 (s, 2H, CH_2_); ^13^C NMR (125 MHz, CD_3_COCD_3_) δ 166.0, 160.8, 160.6, 154.5, 154.1, 151.6, 150.0, 140.5, 138.1, 137.1, 137.0, 136.8, 134.0, 129.7, 128.6, 128.6, 128.5, 128.3, 127.9, 127.7, 124.8, 124.1, 123.8, 123.6, 123.3, 115.3, 115.1, 114.1, 78.4, 69.8; HRMS calcd for C_32_H_25_ClN_4_O_3_ [M+H]^+^: 549.1687, found 549.1691.

#### 3-(((((1E,3Z,4E)-1-(4-((3-methylbenzyl)oxy)phenyl)-5-(pyridin-3-yl)penta-1,4-dien-3-ylidene)amino)oxy)methyl)quinazolin-4(3H)-one (**8p**)

White solid, m.p. 154–156 °C; yield: 29%; IR (KBr, cm^−1^): 3065, 2947, 1741, 1732, 1600, 1506, 1456, 1240, 1172, 1053, 1022, 918, 825, 698; ^1^H NMR (500 MHz, CDCl_3_) δ 8.64–8.60 (m, 1H, Py-2-H), 8.41 (s, 1H, Py-6-H), 8.20 (dd, *J* = 7.9, 0.6 Hz, 1H, Qu-2-H), 7.68 (td, *J* = 7.7, 1.8 Hz, 1H, Qu-5-H), 7.62–7.58 (m, 1H, Py-4-H), 7.46 (d, *J* = 8.7 Hz, 2H, Ar(4-O)-2,6-2H), 7.43–7.34 (m, 3H, Qu-6,7,8-3H), 7.27 (t, *J* = 7.5 Hz, 1H, Ar(3-CH_3_)-5-H), 7.25–7.19 (m, 4H, Ar(3-CH_3_)-2,4,6-3H, Py-5-H), 7.19–7.17 (m, 1H, Ar–CH=), 7.17–7.12 (m, 2H, Ar(4-O)-3,5-2H), 7.08 (d, *J* = 16.7 Hz, 1H, Py–C=CH), 6.95 (d, *J* = 8.8 Hz, 2H, Py–CH=, Ar–C=CH), 6.02 (s, 2H, CH_2_), 5.04 (s, 2H, CH_2_), 2.37 (s, 3H, CH_3_); ^13^C NMR (125 MHz, CDCl_3_) δ 161.4, 160.0, 156.1, 154.5, 149.9, 146.8, 145.6, 138.5, 138.3, 136.8, 136.6, 136.1, 135.4, 134.7, 129.2, 129.0, 128.8, 128.6, 128.4, 127.0, 125.3, 124.9, 124.7, 123.2, 123.1, 122.3, 115.2, 114., 77.5, 70.2, 17.6; HRMS calcd for C_33_H_28_N_4_O_3_ [M+H]^+^: 529.2234, found 529.2235.

## Conclusions

In summary, aiming to develop novel, highly-efficient and environmentally benign agrochemicals, we introduced a quinazolin-4(3*H*)-one scaffold into penta-1,4-diene-3-one oxime ether to synthesis 16 curcumin derivatives, and their antiviral activity against TMV in vivo were evaluated. Antiviral bioassays showed that some compounds exhibited remarkable antiviral activity against TMV. In particular, compounds **8c**, **8j** and **8k** possessed appreciable curative activities against TMV in vivo, with the EC_50_ values of 138.5, 132.9 and 125.6 μg/mL, respectively, which are better than ningnanmycin (207.3 μg/mL). Further studies on the MST and molecular docking experiments of **8k** interaction with TMV CP showed that **8k** bound Arg90 in the TMV CP protein. Arg90 was an important residue to bind TMV RNA. Thus, we speculate that **8k** inhibited the virulence of TMV by binding Arg90 in TMV CP. Given the above results, this kind of penta-1,4-diene-3-one oxime ether derivatives containing a quinazolin-4(3*H*)-one scaffold could be further studied as potential alternative templates in the search for novel antiviral agents.

## Additional file

**Additional file 1.** The data of intermediates **1**, **2**, **3**, **4**, **6**, **7**; all the copies of ^1^H NMR, ^13^C NMR, ^31^P NMR and HRMS for the title compounds; activity confirmed of compounds **8k** were presented in additional information.

## Abbreviations

TMV: tobacco mosaic virus
MST: microscale thermophoresis experiments
TMV CP: TMV coat protein
IR: infrared spectrum
^1^H NMR: ^1^H nuclear magnetic resonance
^13^C NMR: ^13^C nuclear magnetic resonance
HRMS: high resolution mass spectrum
