# Supplementary material for: Design, synthesis, antiviral bioactivities and interaction mechanisms of penta-1,4-diene-3-one oxime ether derivatives containing a quinazolin-4(3H)-one scaffold
Source: BMC Chem. 2019 Mar 25;13(1):34. doi: 10.1186/s13065-019-0547-1 (PMC6661780; doi:10.1186/s13065-019-0547-1)
Supplement: Supplementary file 1 — Additional file 1. The data of intermediates 1, 2, 3, 4, 6, 7; all the copies of 1H NMR, 13C NMR, 31P NMR and HRMS for the title compounds; activity confirmed of compounds 8k were presented in additional information. [file 13065_2019_547_MOESM1_ESM.doc]

**Additional Information**

Design, synthesis, antiviral bioactivities and interaction mechanisms of penta-1,4-diene-3-one oxime ether derivatives containing a quinazolin-4(3*H*)-one scaffold

Lijuan Chen^1,‡^, Xiaobin Wang^1,2,‡^, Xu Tang ^1^, Rongjiao Xia ^1^, Tao Guo ^1^, Cheng Zhang^1^, Xiangyang Li ^1^, Wei Xue1,[[1]](#endnote-2)*

^a^ State Key Laboratory Breeding Base of Green Pesticide and Agricultural Bioengineering, Key Laboratory of Green Pesticide and Agricultural Bioengineering, Ministry of Education, Guizhou University, Huaxi District, Guiyang 550025, China

**^2^** College of Sciences, Nanjing Agricultural University, Nanjing 210095, China

*Corresponding author: Wei Xue; e-mail: wxue@gzu.edu.cn; Tel/Fax: 0086-0851-88292090

^‡^The first and the second authors contributed equally to this work

**Table of Contents**

1. The data of intermediates **1**, **2**, **3**, **4, 6, 7** S3-10

2. ^1^H NMR, ^13^C NMR, IR and HRMS spectrum of the title compounds S11-42

3. Activity confirmed of compound **8k** S43

**1. The data of intermediates 1, 2, 3, 4, 6, 7**

**(E)-4-(4-hydroxyphenyl)but-3-en-2-one (1a):** Yellow solid, m.p. 96-98 °C; yield: 75%; IR (KBr, cm^-1^): 3143, 1597, 1516, 1437, 1364, 1287, 1253, 1167, 972; ^1^H NMR (500MHz, CD_3_OD) *δ*: 7.55 (d, *J* = 16.2 Hz, 1H, Ar-2-H), 7.47 (d, *J* = 1.8 Hz, 1H, Ar-6-H), 7.46 (s, 1H, Ar-CH=), 6.80 (d, *J* = 1.5 Hz, 1H, Ar-3-H), 6.79 (s, 1H, Ar-5-H), 6.58 (d, *J* = 16.2 Hz, 1H, Ar-C=CH), 2.31 (d, *J* = 3.0 Hz, 3H, CH_3_); ^13^C NMR (125MHz, CD_3_OD) *δ*: 204.2, 164.2, 149.0, 134.2, 129.8, 127.4, 119.6, 29.7；HRMS(ESI) calcd for C_10_H_10_O_2_[M+H]^+^: 163.0754, found: 163.0754.

**(E)-4-(2-hydroxyphenyl)but-3-en-2-one (1b):** Yellow solid, m.p. 134-136 °C; yield: 77%; ^1^H NMR (500 MHz, DMSO-*d_6_*) δ: 10.20 (s, 1H, OH), 7.75 (d, *J* = 16.5 Hz, 1H, Ar-CH=), 7.57 (dd, *J* = 7.8, 1.6 Hz, 1H, Ar-6-H), 7.23-7.19 (m, 1H, Ar-4-H), 6.90-6.86 (m, 1H, Ar-5-H), 6.83-6.77 (m, 2H, Ar-3-H, Ar-C=CH), 2.27 (s, 3H, CH_3_). ^13^C NMR (125 MHz, DMSO-*d_6_*) δ: 198.7, 157.4, 139.0, 132.3, 129.1, 127.2, 121.5, 120.0, 116.7, 27.9.

**(1E,4E)-1-(4-hydroxyphenyl)-5-(pyridin-2-yl)penta-1,4-dien-3-one (2a):** Yellow solid；m.p. 153-155 °C; yield: 75%; IR (KBr, cm^-1^): 3036, 1625, 1571, 1514, 1424, 1340, 1172, 1091, 990; ^1^H NMR (500MHz, CD_3_OD) *δ* 8.60 (d, *J* = 4.2 Hz, 1H, Py-6-H), 7.86 (td, *J* = 7.7, 1.7 Hz, 1H, Py-CH), 7.76 (d, *J* = 15.9 Hz, 1H, Ar-CH=), 7.72 (d, *J* = 7.9 Hz, 1H, Ar-2-H), 7.68 (d, *J* = 15.8 Hz, 1H, Ar-6-H), 7.62-7.58 (m, 1H, Py-3-H), 7.57 (s, 1H, Py-4-H), 7.56 (s, 1H, Py-5-H), 7.41-7.37 (m, 1H, Py-C=CH), 7.02 (d, *J* = 15.9 Hz, 1H, Ar-3-H), 6.83 (s, 1H, Ar-5-H), 6.82 (d, *J* = 1.6 Hz, 1H, Ar-C=CH); ^13^C NMR (125MHz, CD_3_OD) *δ* 189.9, 160.7, 153.3, 149.6, 145.3, 140.7, 137.6, 130.6, 128.7, 126.1, 124.6, 124.6, 122.1, 115.7; HRMS(ESI) calcd for C_16_H_13_NO_2_[M+H]^+^: 252.1018, found: 252.1019.

**(1E,4E)-1-(2-hydroxyphenyl)-5-(pyridin-2-yl)penta-1,4-dien-3-one (2b):** Yellow solid；m.p. 158-160 °C; yield: 66%; ^1^H NMR (500 MHz, DMSO-*d_6_*) δ 10.91-9.83 (m, 1H, OH), 8.69-8.60 (m, 1H, Py-6-H), 7.99 (s, 1H, Ar-CH=), 7.96 (s, 1H, Py-CH=), 7.75 (d, J = 7.8 Hz, 1H, Ar-6-H), 7.73-7.71 (m, 1H, Py-5-H), 7.69 (s, 1H, Py-3-H), 7.57 (s, 1H, Py-4-H), 7.53 (s, 1H, Ar-4-H), 7.34 (d, J = 16.1 Hz, 1H, Ar-5-H), 7.27-7.20 (m, 1H, Ar-3-H), 6.91 (dd, J = 8.2, 0.9 Hz, 1H, Py-C=CH), 6.83 (dd, J = 11.4, 4.1 Hz, 1H, Ar-C=CH); ^13^C NMR (125 MHz, DMSO-*d_6_*) δ 189.3, 157.8, 153.4, 150.6, 141.8, 139.1, 137.8, 132.6, 129.4, 129.1, 125.6, 125.2, 121.8, 120.0, 116.8.

**(1E,4E)-1-(4-hydroxyphenyl)-5-(thiophen-2-yl)penta-1,4-dien-3-one (2c):** Yellow solid, yield: 68%; m.p. 207-209 °C; ^1^H NMR (500 MHz, CD_3_COCD_3_) *δ*: 8.97 (s, 1H, OH), 7.92-7.89 (d, *J*=15.4 Hz, 1H, Ar-CH=), 7.72-7.69 (d, *J*=16.0 Hz, 1H, thiophene-CH=), 7.65-7.63 (m, 3H, thiophene-5-H, Ar-2,6-H), 7.51 (s, 1H, thiophene-3-H), 7.17-7.16 (m, 1H, thiophene-4-H), 7.13-7.10 (d, *J*=15.4 Hz, 1H, Ar-C=CH), 7.01-6.97 (d, *J*=16.0 Hz, 1H, thiophene-C=CH), 6.93-6.91 (m, 2H, Ar-3,5-H); ^13^C NMR (125 MHz, CD_3_COCD_3_) *δ* 187.3, 159.9, 142.7, 140.5, 134.6, 131.8, 130.5, 128.9, 128.5, 126.8, 124.8, 122.8, 115.9.

**(1E,4E)-1-(4-hydroxyphenyl)-5-(pyridin-3-yl)penta-1,4-dien-3-one (2d):** Yellow solid, m.p. 173-175 °C; yield: 75%; ^1^H NMR (500 MHz, DMSO-*d_6_*) δ 10.10 (s, 1H, OH), 8.91 (d, *J* = 1.9 Hz, 1H, Py-2-H), 8.56 (dd, *J* = 4.7, 1.3 Hz, 1H, Py-6-H), 8.19 (dt, *J* = 8.0, 1.6 Hz, 1H, Py-4-H), 7.73 (dd, *J* = 18.5, 16.1 Hz, 2H, Py-CH=, Ar-CH=), 7.61 (d, *J* = 8.6 Hz, 2H, Ar-2,6-2H), 7.50-7.42 (m, 2H, Py-5-H, Ar-3-H), 7.06 (d, *J* = 16.0 Hz, 1H, Ar-5-H), 6.83 (t, *J* = 9.2 Hz, 2H, Ar-C=CH, Py-C=CH). ^13^C NMR (125 MHz, DMSO-*d_6_*) δ 188.6, 160.7, 151.3, 150.5, 144.4, 139.0, 135.2, 131.2, 127.8, 126.2, 124.5, 123.1, 116.5.

**(1E,4E)-1-(4-((3-chlorobenzyl)oxy)phenyl)-5-(pyridin-2-yl)penta-1,4-dien-3-one (3a):**

Yellow solid, m.p. 143-145 °C; yield: 58%; IR (KBr, cm^-1^): 1717, 1623, 1580, 1563, 1490, 1387, 1325, 1225, 1183, 1169, 1088, 978, 935, 878, 774; ^1^H NMR (500 MHz, DMSO-*d_6_*) δ 8.65 (s, 1H, Py-6-H), 8.04-7.64 (m, 6H, Ar(4-O)-2,6-2H, Py-CH=, Ar-CH=, Ar(3-Cl)-2,4-2H), 7.66-7.47 (m, 3H, Py-3,4,5-3H), 7.46-7.33 (m, 3H, Ar(3-Cl)-5,6-2H, Ar(4-O)-3-H), 7.26 (dd, *J* = 16.0, 8.1 Hz, 1H, Ar(4-O)-5-H), 7.09 (d, *J* = 8.4 Hz, 2H, Py-C=CH, Ar-C=CH), 5.18 (d, *J* = 8.0 Hz, 2H, CH_2_).^13^C NMR (125 MHz, DMSO-*d_6_*) δ 189.1, 160.7, 153.5, 150.6, 143.6, 141.9, 139.8, 137.8, 133.7, 131.2, 131.0, 129.3, 128.4, 128.2, 128.0, 126.8, 125.6, 125.2, 124.1, 115.8, 69.0. HRMS(ESI) calcd for C_23_H_18_ClNO_2_ [M+H]^+^: 376.1096, found: 376.1099.

**(1E,4E)-1-(4-((3-methylbenzyl)oxy)phenyl)-5-(pyridin-2-yl)penta-1,4-dien-3-one (3b):** Yellow solid, m.p. 115-117 °C; yield: 65%; IR (KBr, cm^-1^): 3037, 2876, 1653, 1591, 1575, 1511, 1326, 1240, 1172, 1092, 998, 821, 712; ^1^H NMR (500 MHz, CD_3_COCD_3_) δ 8.68-8.61 (m, 1H, Py-6-H), 7.83 (td, *J* = 7.7, 1.8 Hz, 1H, Py-CH), 7.76-7.72 (m, 2H, Ar(4-O)-2,6-2H), 7.72 (s, 1H, Ar(4-O)-CH), 7.69 (s, 1H, Ar(3-CH_3_)-5-H), 7.67 (dd, *J* = 7.8, 0.8 Hz, 1H, Py-3-H), 7.63-7.59 (m, 1H, Py-4-H), 7.36 (ddd, *J* = 7.5, 4.7, 1.1 Hz, 1H, Py-5-H), 7.28 (s, 1H, Ar(3-CH_3_)-6-H), 7.27-7.25 (m, 1H, Ar(4-O)-3-H), 7.25 (d, *J* = 1.0 Hz, 1H, Ar(4-O)-5-H), 7.18 (d, *J* = 16.0 Hz, 1H, Ar(3-CH_3_)-4-H), 7.15-7.11 (m, 1H, Py-C=CH), 7.09-7.07 (m, 1H, Ar(3-CH_3_)-2-H), 7.07-7.05 (m, 1H, Ar(4-O)-C=CH), 5.13 (s, 2H, CH_2_), 2.32 (s, 3H, CH_3_). ^13^C NMR (125 MHz, CD_3_COCD_3_) δ 188.2, 161.1, 153.6, 150.2, 142.9, 141.1, 138.1, 137.0, 136.9, 130.4, 129.0, 128.7, 128.5, 128.4, 127.9, 124.8, 124.7, 124.4, 123.6, 115.4, 69.89, 20.5. HRMS(ESI) calcd for C_24_H_21_NO_2_[M+H]^+^: 356.1642, found: 356.1648.

**(1E,4E)-1-(4-((2-chlorobenzyl)oxy)phenyl)-5-(pyridin-2-yl)penta-1,4-dien-3-one (3c):** Yellow solid, m.p. 146-148 °C; yield: 51%; ^1^H NMR (500 MHz, CDCl_3_) δ 7.85 (d, *J* = 15.5 Hz, 1H, Py-6-H), 7.69 (d, *J* = 15.9 Hz, 1H, Ar(4-O)-2-H), 7.58 (d, *J* = 1.9 Hz, 1H, Ar(4-O)-6-H), 7.56 (d, *J* = 2.8 Hz, 1H, Py-CH=), 7.54 (dd, *J* = 7.0, 2.2 Hz, 1H, Ar-CH=), 7.43 – 7.41 (m, 1H, Ar(2-Cl)-3-H), 7.40 (d, *J* = 4.1 Hz, 1H, Py-3-H), 7.33 (d, *J* = 3.5 Hz, 1H, Py-4-H), 7.30- 7.27 (m, 2H, Py-5-H, Ar(2-Cl)-6-H), 7.08 (dd, *J* = 5.0, 3.8 Hz, 1H, Ar(2-Cl)-4-H), 7.03- 7.01 (m, 1H, Ar(2-Cl)-5-H), 7.00 (d, *J* = 2.8 Hz, 1H, Ar(4-O)-3-H), 6.89 (dd, *J* = 17.0, 15.8 Hz, 3H, Py-C=CH, Ar-C=CH, Ar(4-O)-5-H), 5.21 (s, 2H, CH_2_). ^13^C NMR (125 MHz, CDCl_3_) δ 188.4, 160.6, 142.9, 140.5, 135.5, 134.2, 132.7, 131.8, 130.3, 129.6, 129.3, 128.9, 128.7, 128.4, 128.1, 127.1, 124.5, 123.8, 115.4, 67.3.

**(1E,4E)-1-(4-((4-chlorobenzyl)oxy)phenyl)-5-(pyridin-2-yl)penta-1,4-dien-3-one (3d):** Yellow solid, m.p. 177-179 °C; yield: 65%; ^1^H NMR (500 MHz, CDCl_3_-*d_6_*) δ 8.67 (d, *J* = 4.3 Hz, 1H, Py-6-H), 7.73 (m, 2H, Ar(4-O)-2,6-2H), 7.68 (s, 1H, Ar-CH=), 7.62 (d, *J* = 15.5 Hz, 1H, Py-CH=), 7.56 (t, *J* = 5.7 Hz, 2H, Py-3,4-2H), 7.47 (d, *J* = 7.8 Hz, 1H, Py-5-H), 7.36 (s, 4H, Ar(4-Cl)-2,3,5,6-4H), 7.30-7.26 (m, 1H, Py-C=CH), 6.97 (dd, *J* = 12.3, 11.0 Hz, 3H, Ar(4-O)-3,5-2H, Ar-C=CH), 5.07 (s, 2H, CH_2_). ^13^C NMR (125 MHz, CDCl_3_-*d_6_*) δ 189.2, 160.7, 153.5, 150.2, 143.7, 141.2, 137.0, 135.0, 134.1, 130.3, 129.0, 128.9, 128.5, 128.0, 125.1, 124.4, 124.2, 115.4, 69.4.

**(1E,4E)-1-(4-((4-chlorobenzyl)oxy)phenyl)-5-(thiophen-2-yl)penta-1,4-dien-3-one(3e):** Yellow solid, m.p. 94-96 °C; yield: 87%; ^1^H NMR (500 MHz, CDCl_3_) δ 7.84 (d, *J* = 15.6 Hz, 1H, Ar(4-O)-2-H), 7.68 (d, *J* = 15.8 Hz, 1H, Ar(4-O)-6-H), 7.56 (d, *J* = 8.7 Hz, 2H, Ar-CH=, Thiophene-5-H), 7.40 (d, *J* = 5.1 Hz, 1H, Thiophene-3-H), 7.36 (s, 4H, Thiophene-CH=, Thiophene-4-H, Ar(4-Cl)-3,5-2H), 7.32 (d, *J* = 3.6 Hz, 1H, Ar(4-O)-3-H), 7.08 (dd, *J* = 5.0, 3.6 Hz, 1H, Ar(4-O)-5-H), 6.97 (d, *J* = 8.7 Hz, 2H, Ar(4-Cl)-2,6-2H), 6.88 (t, *J* = 16.1 Hz, 2H, Ar-C=CH, Thiophene-C=CH), 5.07 (s, 2H, CH_2_). ^13^C NMR (125 MHz, CDCl_3_) δ 188.3, 160.6, 142.9, 140.5, 135.5, 135.0, 134.1, 131.8, 130.3, 129.0, 128.9, 128.7, 128.4, 128.1, 124.5, 123.9, 115.4, 69.4.

**(1E,4E)-1-(4-((2,4-dichlorobenzyl)oxy)phenyl)-5-(thiophen-2-yl)penta-1,4-dien-3-one (3f):** Yellow solid, m.p. 111-113 °C; yield: 76%; ^1^H NMR (500 MHz, CDCl_3_) δ 7.85 (d, *J* = 15.6 Hz, 1H, Ar(4-O)-2-H), 7.68 (d, *J* = 15.9 Hz, 1H, Ar(4-O)-6-H), 7.57 (d, *J* = 8.7 Hz, 2H, Ar-CH=, Thiophene-5-H), 7.48 (d, *J* = 8.3 Hz, 1H, Ar(2,4-di-Cl)-3-H), 7.43 (d, *J* = 2.1 Hz, 1H, Thiophene-3-H), 7.40 (d, *J* = 4.8 Hz, 1H, Thiophene-CH=), 7.33 (d, *J* = 3.6 Hz, 1H, Ar(2,4-di-Cl)-5-H), 7.28 (dd, *J* = 8.3, 2.0 Hz, 1H, Thiophene-4-H), 7.09-7.06 (m, 1H, Ar(2,4-di-Cl)-6-H), 6.99 (d, *J* = 8.7 Hz, 2H, Ar(4-O)-3,5-2H), 6.88 (dd, *J* = 18.4, 15.8 Hz, 2H, Thiophene-C=CH, Ar-C=CH), 5.16 (s, 2H, CH_2_). ^13^C NMR (125 MHz, CDCl_3_) δ 188.3, 160.3, 142.8, 140.5, 135.5, 134.5, 133.3, 132.9, 131.8, 130.3, 129.7, 129.4, 128.8, 128.4, 128.3, 127.5, 124.5, 124.0, 115.4, 66.8.

**(1E,3E,4E)-1-(4-((3-chlorobenzyl)oxy)phenyl)-5-(pyridin-2-yl)penta-1,4-dien-3-one oxime (4a):** White solid, m.p. 178-180 °C; yield: 75%; IR (KBr, cm^-1^): 3066, 2927, 2773, 1592, 1508, 1465, 1378, 1231, 1188, 1016, 848; ^1^H NMR (500 MHz, DMSO-*d_6_*) δ 11.64 (s, 1H, OH), 8.55 (d, *J* = 4.3 Hz, 1H, Py-6-H), 7.79-7.72 (m, 1H, Ar(4-O)-2-H), 7.58 (d, *J* = 2.5 Hz, 1H, Ar(4-O)-6-H), 7.56 (d, *J* = 2.5 Hz, 1H, Ar(3-Cl)-2-H), 7.50~7.42 (m, 2H, Py-3,4-2H), 7.40 (s, 1H, Py-5-H), 7.39 (s, 1H, Ar(3-Cl)-4-H), 7.38-7.36 (m, 1H, Ar(3-Cl)-5-H), 7.36 (d, *J* = 5.8 Hz, 1H, Ar(3-Cl)-6-H), 7.25 (dd, *J* = 7.4, 5.1 Hz, 1H, Ar-CH=), 7.18 (s, 2H Ar(4-O)-3,5-2H,), 7.12 (d, *J* = 16.0 Hz, 1H, Py-C=CH), 7.02 (d, *J* = 8.7 Hz, 2H, Ar-C=CH, Py-CH=), 5.14 (s, 2H, CH_2_). ^13^C NMR (125 MHz, DMSO-*d_6_*) δ 189.0, 160.7, 153.5, 150.6, 143.6, 141.9, 139.8, 137.8, 133.7, 131.2, 131.0, 129.3, 128.4, 128.2, 128.0, 126.8, 125.6, 125.2, 124.1, 115.8, 69.0. HRMS(ESI) calcd for C_23_H_19_ClN_2_O_2_[M+H]^+^: 391.1199, found: 391.1195.

**(1E,3E,4E)-1-(4-((3-methylbenzyl)oxy)phenyl)-5-(pyridin-2-yl)penta-1,4-dien-3-one oxime (4b):** White solid, m.p. 211-213 °C; yield: 35%; IR (KBr, cm^-1^): 3059, 2929, 2778, 1602, 1510, 1477, 1267, 1241, 1177, 1028, 1001, 979, 808; ^1^H NMR (500 MHz, DMSO-*d_6_*) δ 11.62 (s, 1H, OH), 8.55 (d, *J* = 4.2 Hz, 1H, Py-6-H), 7.76 (td, *J* = 7.7, 1.7 Hz, 1H, Py-CH), 7.58 (s, 1H, Ar(4-OCH_2_)-2-H), 7.57 (s, 1H, Ar(4-OCH_2_)-6-H), 7.55 (s, 1H, Ar(4-OCH_2_)-CH), 7.42 (d, *J* = 16.0 Hz, 1H, Ar(3-CH_3_)-5-H), 7.27–7.25 (m, 1H, Py-3-H), 7.24 (t, *J* = 1.7 Hz, 1H, Py-4-H), 7.23 (s, 1H, Py-5-H), 7.20 (d, *J* = 7.2 Hz, 1H, Ar(3-CH_3_)-6-H), 7.17 (s, 2H, Ar(4-OCH_2_)-3,5-2H), 7.14 (s, 1H, Ar(3-CH_3_)-4-H), 7.11 (t, *J* = 3.5 Hz, 1H, Py-C=CH), 7.01 (s, 1H, Ar(3-CH_3_)-2-H), 6.99 (s, 1H, Ar(4-OCH_2_)-C=CH), 5.07 (s, 2H, CH_2_), 2.28 (s, 3H, CH_3_); ^13^C NMR (125 MHz, DMSO-*d_6_*) δ 159.5, 155.0, 152.6, 150.1, 138.2, 137.4, 137.3, 135.8, 132.2, 129.6, 129.2, 129.1, 128.9, 128.8, 127.3, 125.4, 123.4, 115.7, 115.2, 69.8, 21.5. HRMS(ESI) calcd for C_24_H_22_N_2_O_2_[M+H]^+^: 371.1750, found: 371.1761.

**(1E,3E,4E)-1-(2-((2-chlorobenzyl)oxy)phenyl)-5-(pyridin-2-yl)penta-1,4-dien-3-one oxime (4c):** White solid, m.p. 164-166 ^o^C; yield: 31%; ^1^H NMR (500 MHz, DMSO-*d_6_*) δ 11.69 (d, *J* = 3.0 Hz, 1H, OH), 8.68-8.45 (m, 1H, Py-6-H), 7.78-7.73 (m, 1H, Ar(2-Cl)-3-H), 7.69 (dd, *J* = 7.7, 1.5 Hz, 1H, Ar(2-O)-6-H), 7.59 (m, 1H, Py-3-H), 7.49 (t, *J* = 13.5 Hz, 1H, Py-4-H), 7.46-7.41 (m, 2H, Ar(2-Cl)-4,6-2H), 7.41-7.36 (m, 2H, Py-5-H, Ar(2-Cl)-5-H), 7.36-7.29 (m, 3H, Py-C=CH, Ar(2-O)-4-H, Ar-CH=), 7.29-7.24 (m, 2H, Ar(2-O)-3,5-H), 7.21 (m, 1H, Ar-C=CH), 7.17 (d, *J* = 7.8 Hz, 1H, Py-CH=), 5.21 (s, 2H, CH_2_); ^13^C NMR (125 MHz, DMSO-*d_6_*) δ 156.4, 154.8, 153.1, 150.2, 137.5, 134.7, 133.1, 132.4, 130.8, 130.4, 130.3, 129.9, 127.8, 127.5, 127.4, 125.7, 123.5, 123.4, 121.9, 120.0, 117.7, 113.7, 68.0.

**(1E,3E,4E)-1-(2-((2,4-dichlorobenzyl)oxy)phenyl)-5-(pyridin-2-yl)penta-1,4-dien-3-one oxime (4d):** White solid, m.p. 161-163 ^o^C; yield: 37%; ^1^H NMR (500 MHz, DMSO-*d_6_*) δ 11.70 (s, 1H, OH), 8.54 (d, J = 4.1 Hz, 1H, Py-6-H), 7.72 (m, 2H, Ar(2,4-2Cl)-3-H, Ar(2-O)-6-H), 7.43 (m, 4H, Ar(2,4-2Cl)-5-H, Py-3,4,5-3H), 7.36-7.23 (m, 5H, Ar(2,4-2Cl)-6-H, Ar(2-O)-4,5-2H, Ar-CH=, Py-C=CH), 7.13-7.06 (m, 2H, Ar-C=CH, Ar(2-O)-3-H), 7.00 (t, J = 7.5 Hz, 1H, Py-CH=), 5.18 (s, 2H, CH_2_); ^13^C NMR (125 MHz, DMSO-*d_6_*) δ 156.3, 154.8, 153.1, 150.1, 137.5, 134.2, 134.0, 133.9, 132.4, 132.0, 131.8, 130.9, 129.5, 128.0, 127.5, 125.7, 124.1, 123.4, 122.1, 121.0, 117.8, 113.7, 67.4.

**(1E,3E,4E)-1-(4-((3-methylbenzyl)oxy)phenyl)-5-(pyridin-3-yl)penta-1,4-dien-3-one oxime (4e):** White solid, m.p. 161-163 ^o^C; yield: 43%; ^1^H NMR (500 MHz, DMSO-*d_6_*) δ 11.69 (d, *J* = 1.3 Hz, 1H, OH), 8.54 (dd, *J* = 4.7, 0.9 Hz, 1H, Py-3-H), 7.73 (td, *J* = 7.7, 1.8 Hz, 1H, Py-6-H), 7.67 (dd, *J* = 7.7, 1.5 Hz, 1H, Ar(4-O)-2-H), 7.50 (d, *J* = 17.0 Hz, 1H, Ar(4-O)-6-H), 7.45-7.34 (m, 3H, Ar(3-CH_3_)-2-H, Py-5,4-2H), 7.34-7.24 (m, 3H, Ar(3-CH_3_)-4,5,6-3H), 7.23-7.14 (m, 2H, Ar(4-O)-3,5-2H), 7.17-7.16 (m, 1H, Ar-CH=), 7.15-7.03 (m, 2H, Py-C=CH, Ar-C=CH), 6.98 (t, *J* = 7.4 Hz, 1H, Py-CH=), 5.11 (s, 2H,), 2.16 (s, 3H); ^13^C NMR (125 MHz, DMSO-*d_6_*) δ 156.7, 154.8, 153.2, 150.1, 138.2, 137.5, 137.4, 132.5, 131.1, 130.8, 128.9, 128.8, 128.4, 127.6, 127.5, 125.5, 125.5, 124.9, 123.4, 123.4, 121.6, 120.0, 117.8, 113.6, 70.2, 21.4.

**3-(hydroxymethyl)quinazolin-4(3H)-one (6a):** White solid; m.p. >250 ^o^C; yield: 86.3%; ^1^H NMR (500 MHz, DMSO-*d_6_*) δ 8.51 (s, 1H, Qu-2-H), 8.16 (dd, J = 8.0, 1.3 Hz, 1H, Qu-5-H), 7.80 (m, 1H, Qu-7-H), 7.61 (dd, J = 7.8 Hz, 1H, Qu-8-H), 7.59 (m, 1H, Qu-6-H), 5.14 (s, 2H, CH_2_).

**6-chloro-3-(hydroxymethyl)quinazolin-4(3H)-one (6b):** White solid; m.p. 220-222 ^o^C; yield: 90.9%; ^1^H NMR (500 MHz, DMSO-*d_6_*) δ 8.37 (s, 1H, Qu-2-H), 8.30 (d, *J* = 2.3 Hz, 1H, Qu-5-H), 7.70 (dd, 1H, Qu-7-H), 7.24 (d, 1H, Qu-8-H), 5.04 (s, 2H, CH_2_).

**3-(chloromethyl)quinazolin-4(3H)-one (7a):** White solid, m.p. 113-115 ^o^C; yield: 61.7%; ^1^H NMR (500 MHz, CDCl_3_) δ 8.54 (s, 1H, Qu-2-H), 7.98 (dd, *J*=8.00 HZ, 1H, Qu-5-H), 7.68 (td, *J=*8.40, 7.45 Hz, 1H, Qu-7-H), 7.69 (td, *J=*8.00, 7.45 Hz, 1H, Qu-6-H), 7.49 (dd, 1H, Qu-8-H), 5.91 (s, 2H, CH_2_).

**6-chloro-3-(chloromethyl) quinazolin-4(3H**)-**one(7b):** White solid, m.p. 115-117 ^o^C; yield: 60.5%; ^1^H NMR (500 MHz, CDCl_3_) δ 8.37 (s, 1H, Qu-2-H), 8.29 (d, J = 2.3 Hz, 1H, Qu-5-H), 7.65 (dd, J = 10.3, 5.2 Hz, 1H, Qu-7-H), 7.37 (d, 1H, Qu-8-H), 5.04 (s, 2H, CH_2_).

2. ^1^H NMR, ^13^C NMR , IR and HRMS spectrum of the title compounds

Figure S1. ^1^H NMR spectrum of compound **8a**

**
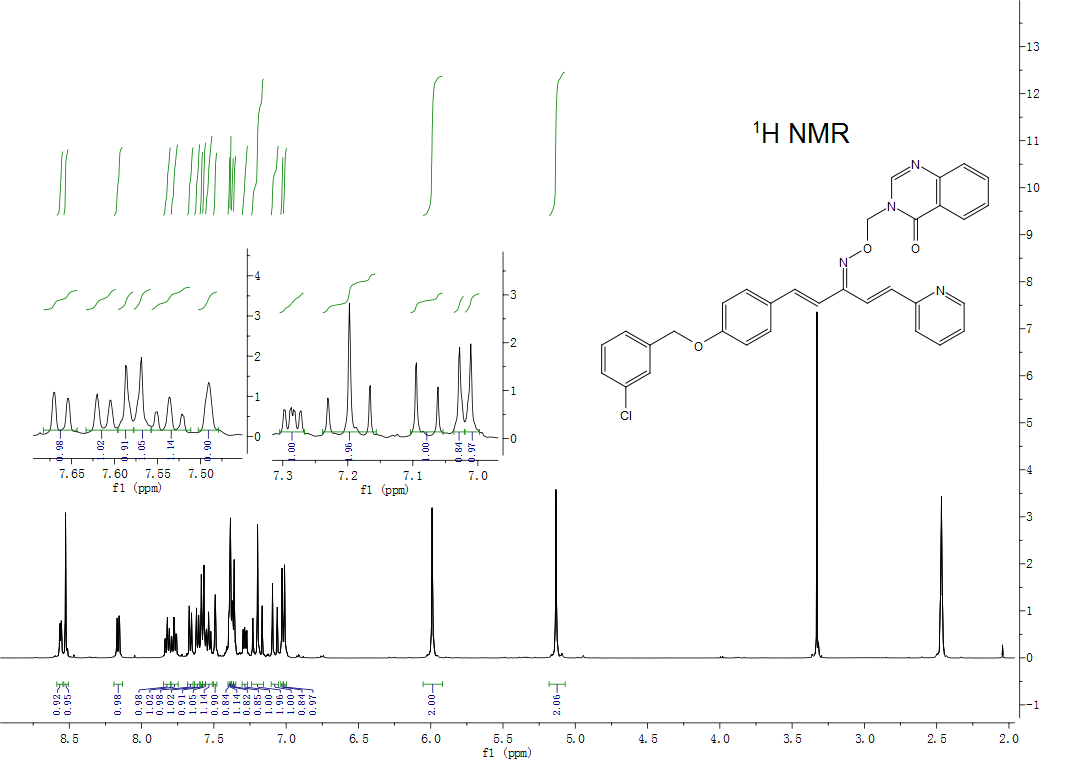
**

Figure S2. ^13^C NMR spectrum of compound **8a**

**
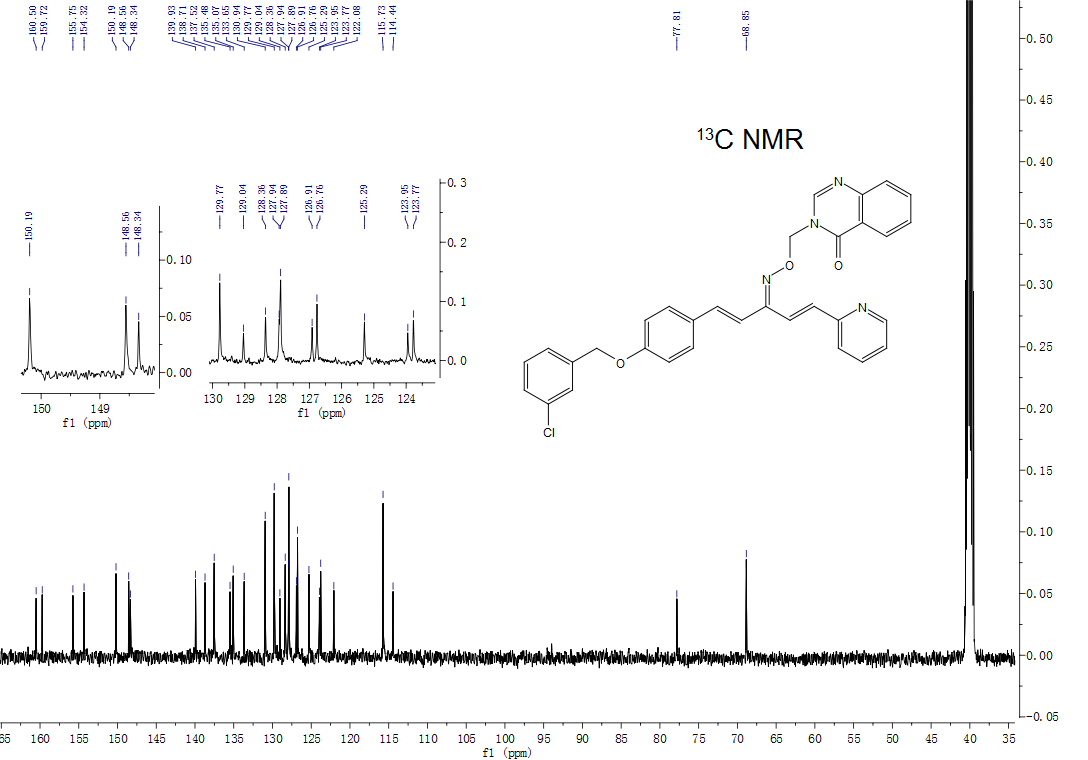
**

Figure S3. IR spectrum of compound **8a**


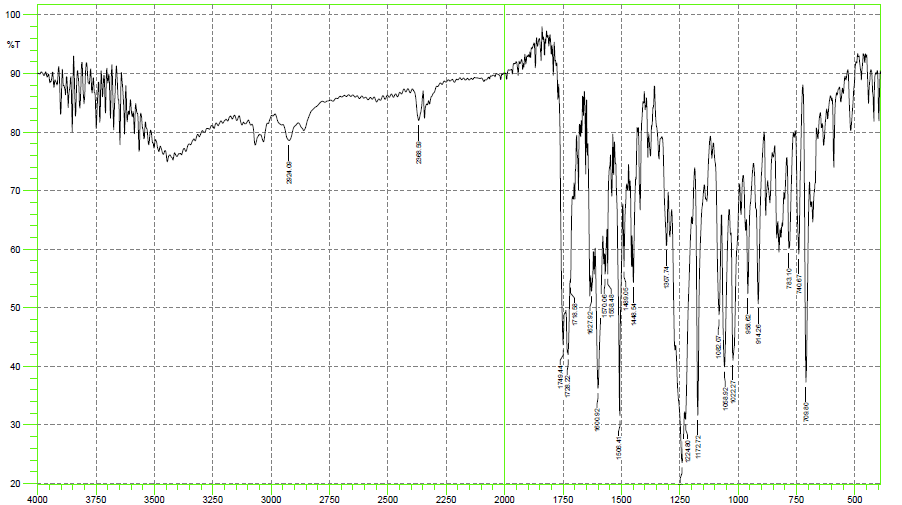


Figure S4. HRMS spectrum of compound **8a**

Figure S5. ^1^H NMR spectrum of compound **8b**

**
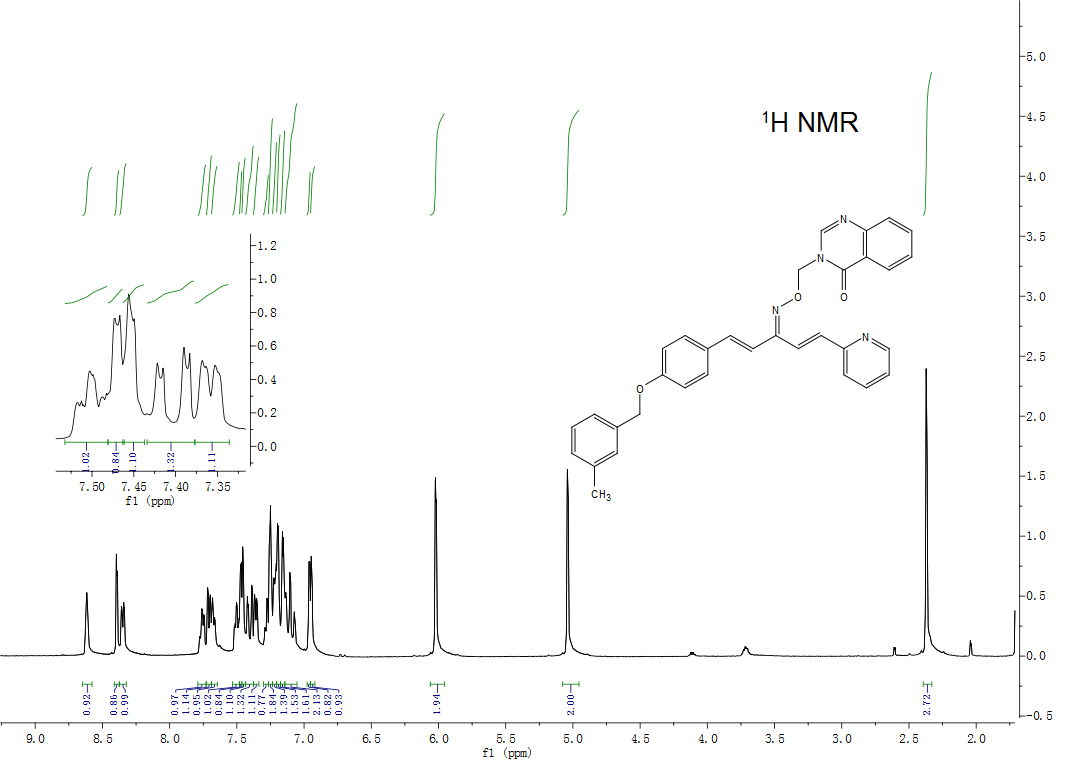
**

Figure S6. ^13^C NMR spectrum of compound **8b**

**
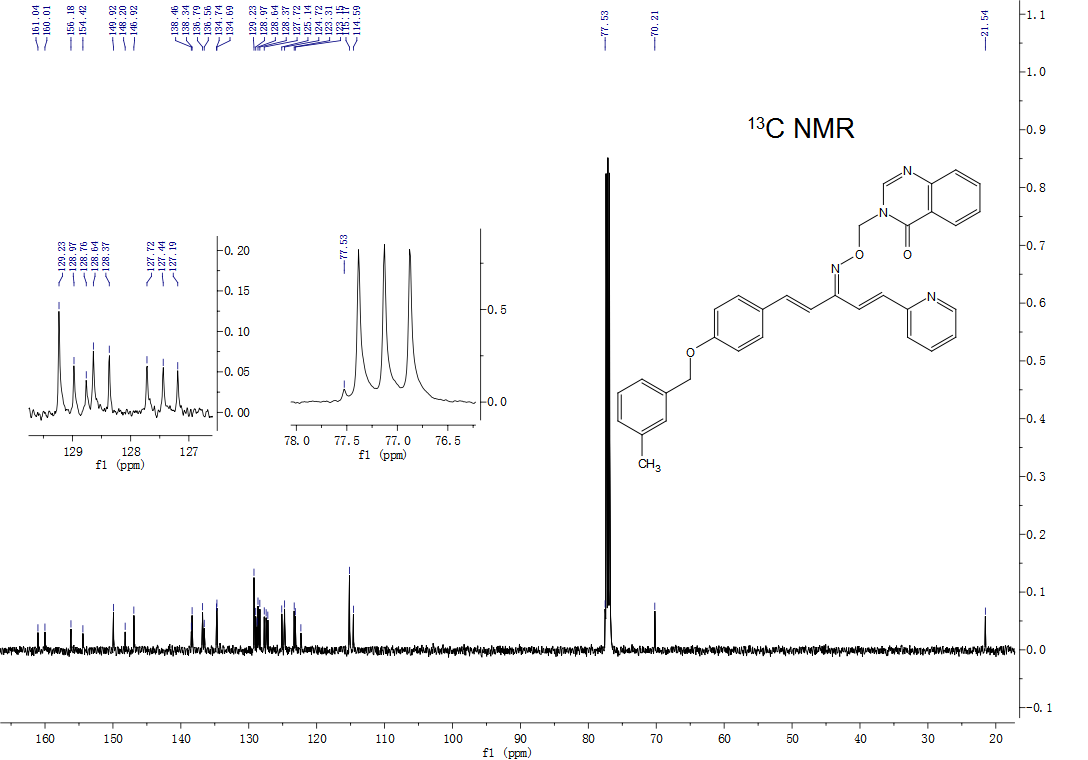
**

Figure S7. IR spectrum of compound **8b**


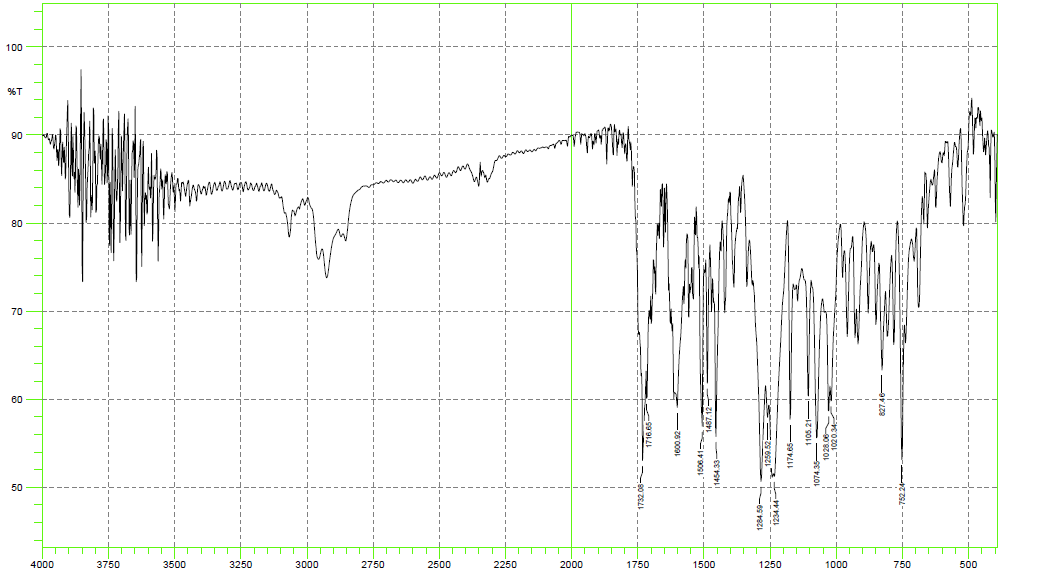


Figure S8. HRMS spectrum of compound **8b**

Figure S9. ^1^H NMR spectrum of compound **8c**


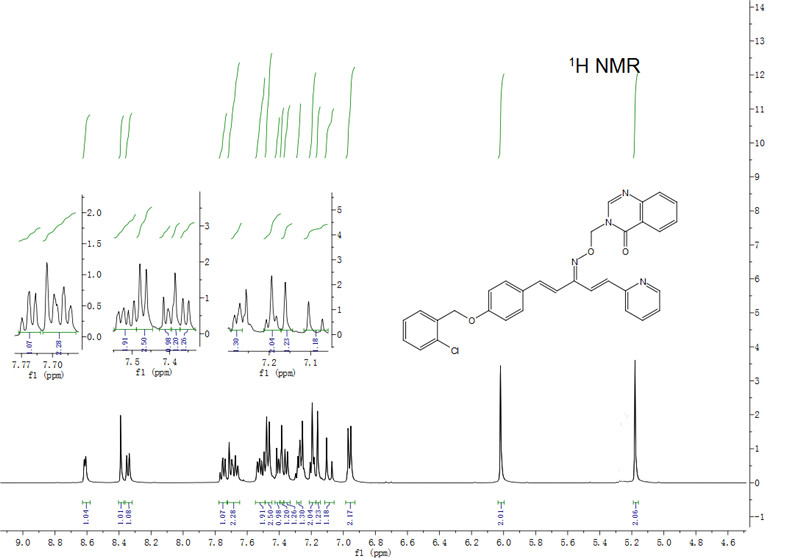


Figure S10. ^13^C NMR spectrum of compound **8c**


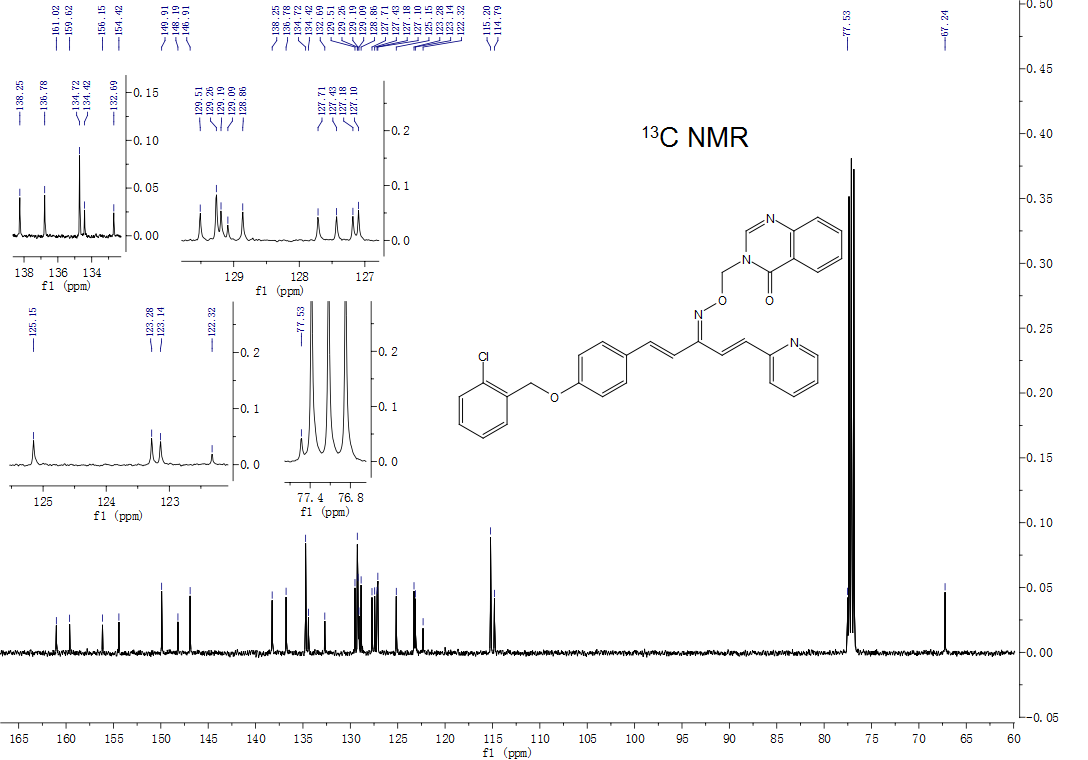


Figure S11. IR spectrum of compound 8c


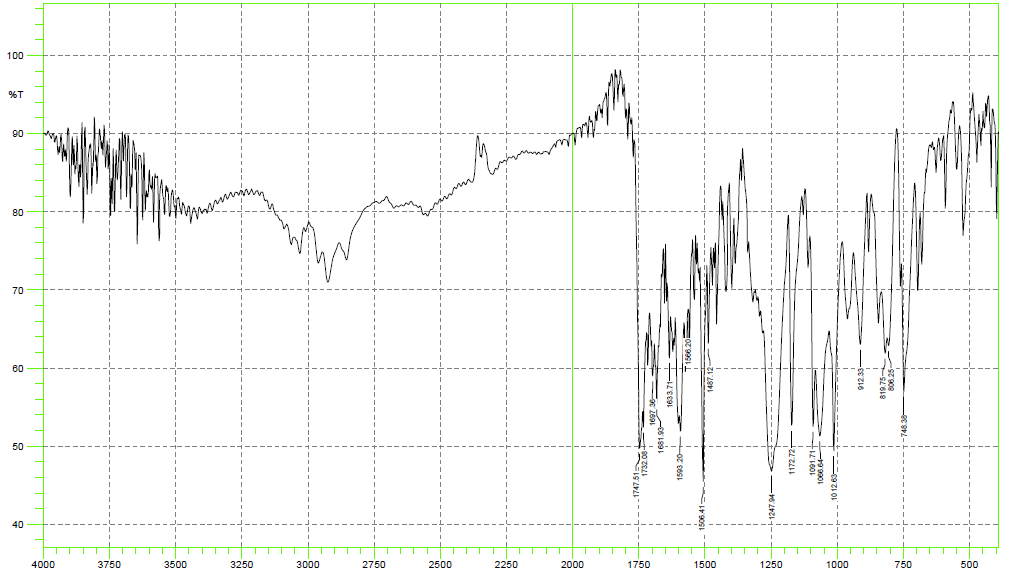


Figure S12. MS spectrum of compound 8c

Figure S13. ^1^H spectrum of compound 8d


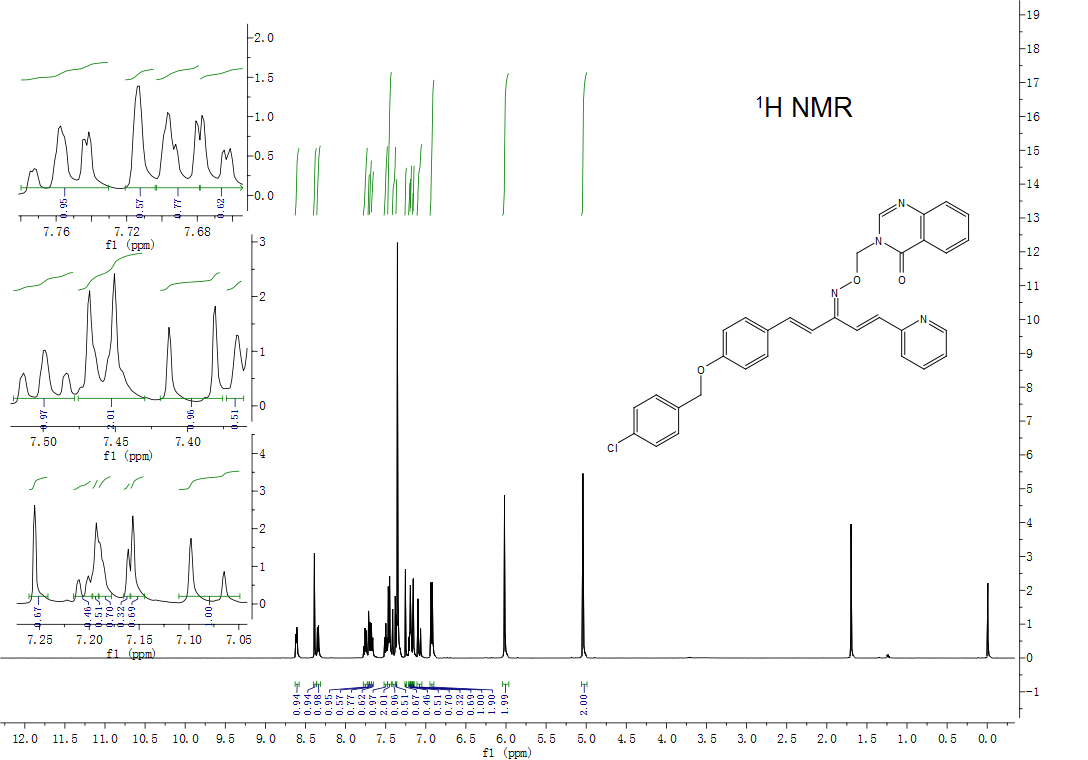


Figure S14. ^13^C NMR spectrum of compound 8d


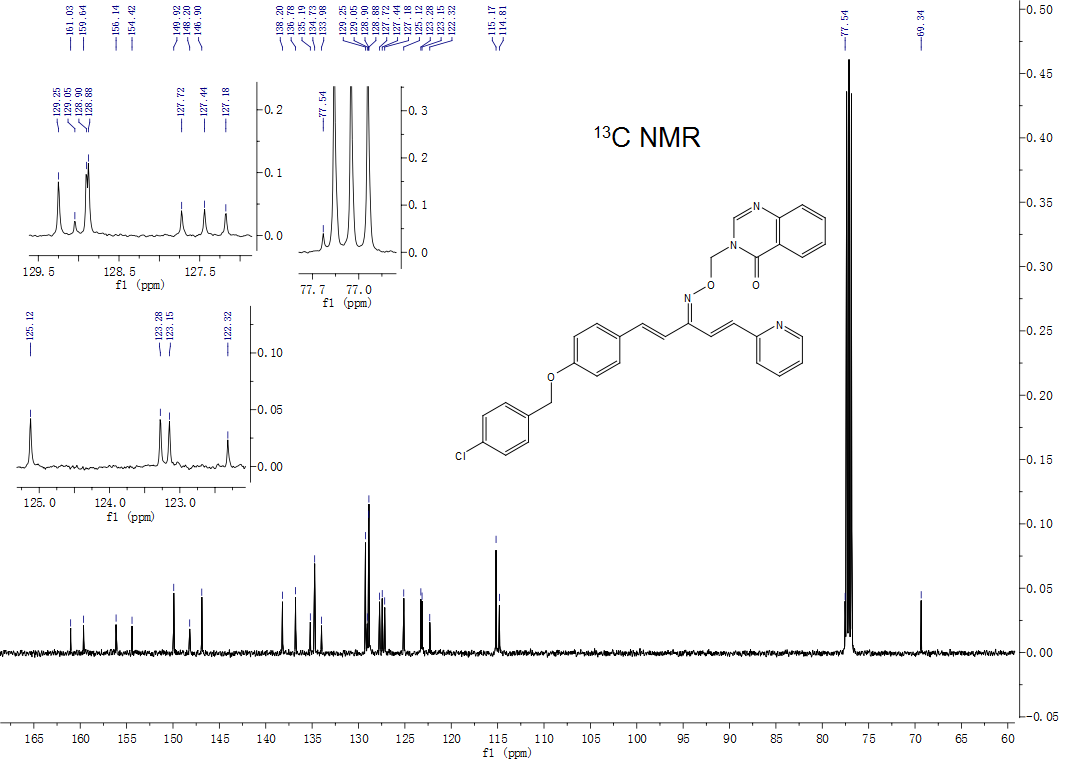


Figure S15. IR spectrum of compound 8d


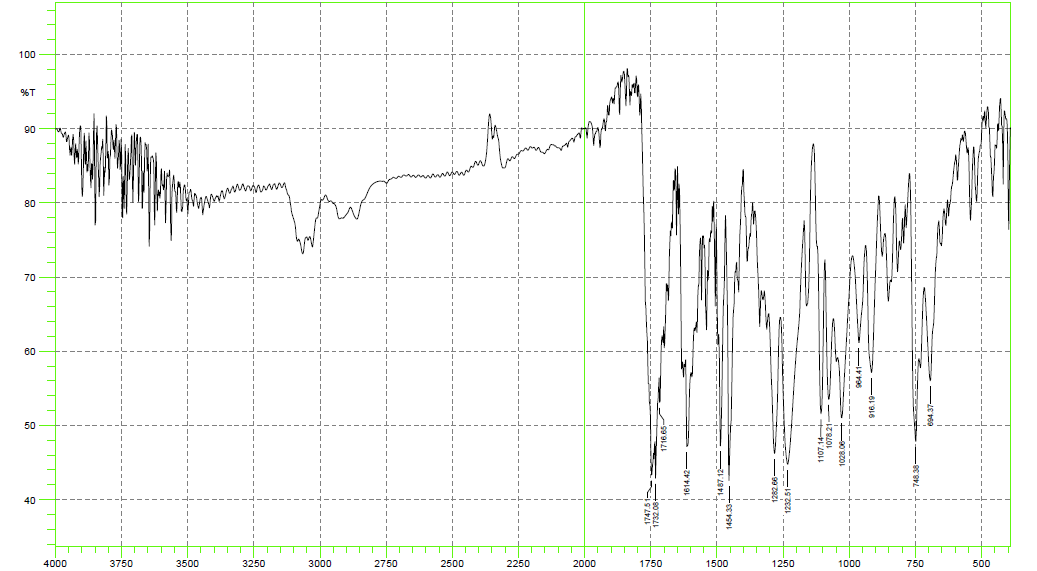


Figure S16. HRMS spectrum of compound 8d

Figure S17. ^1^HNMR spectrum of compound 8e


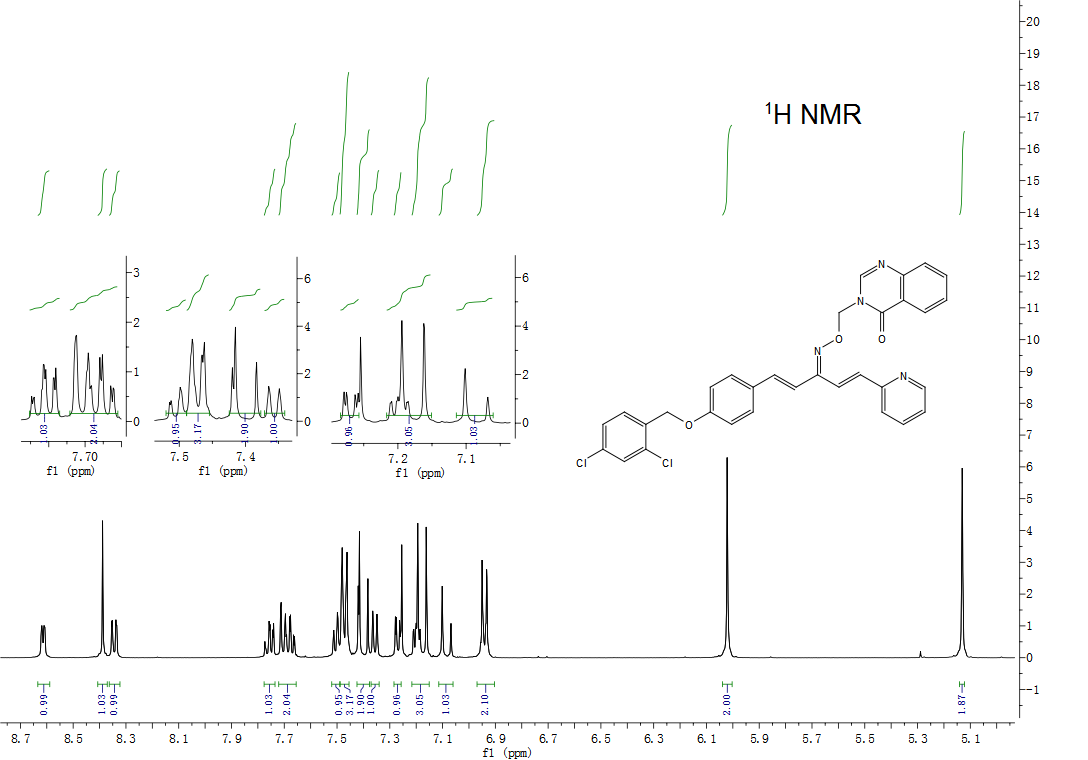


Figure S18.^13^C NMR spectrum of compound 8e


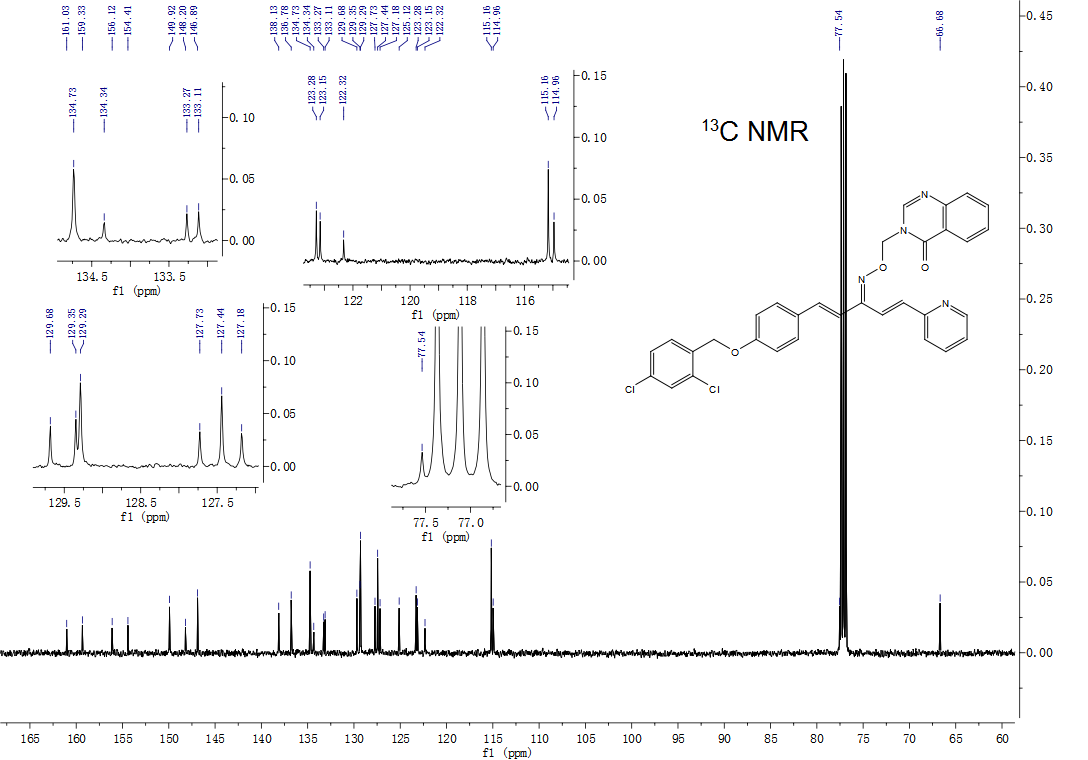


Figure S19. IR spectrum of compound 8e


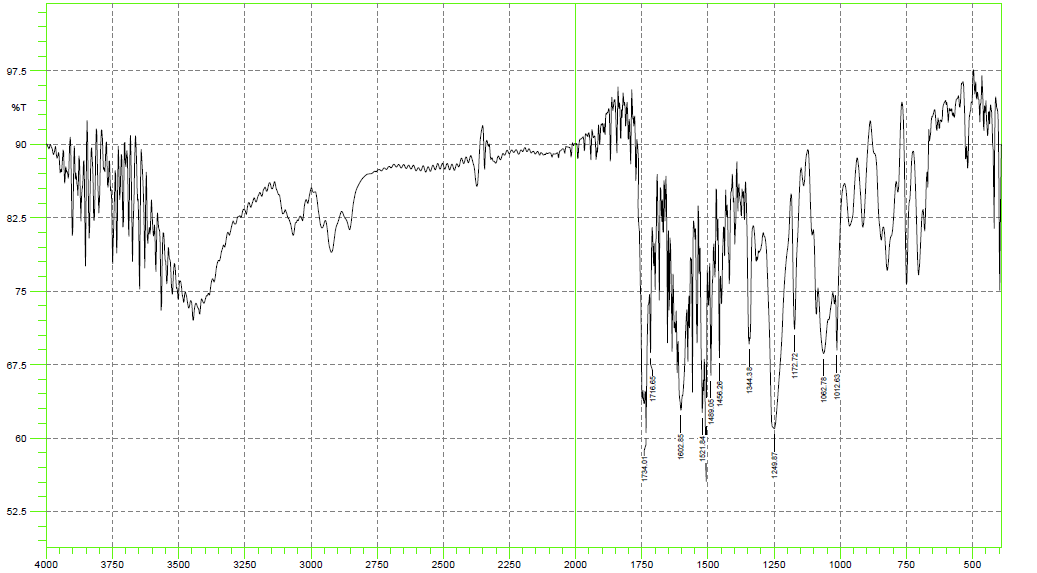


Figure S20. HRMS spectrum of compound 8e

Figure S21. ^1^H NMR spectrum of compound 8f


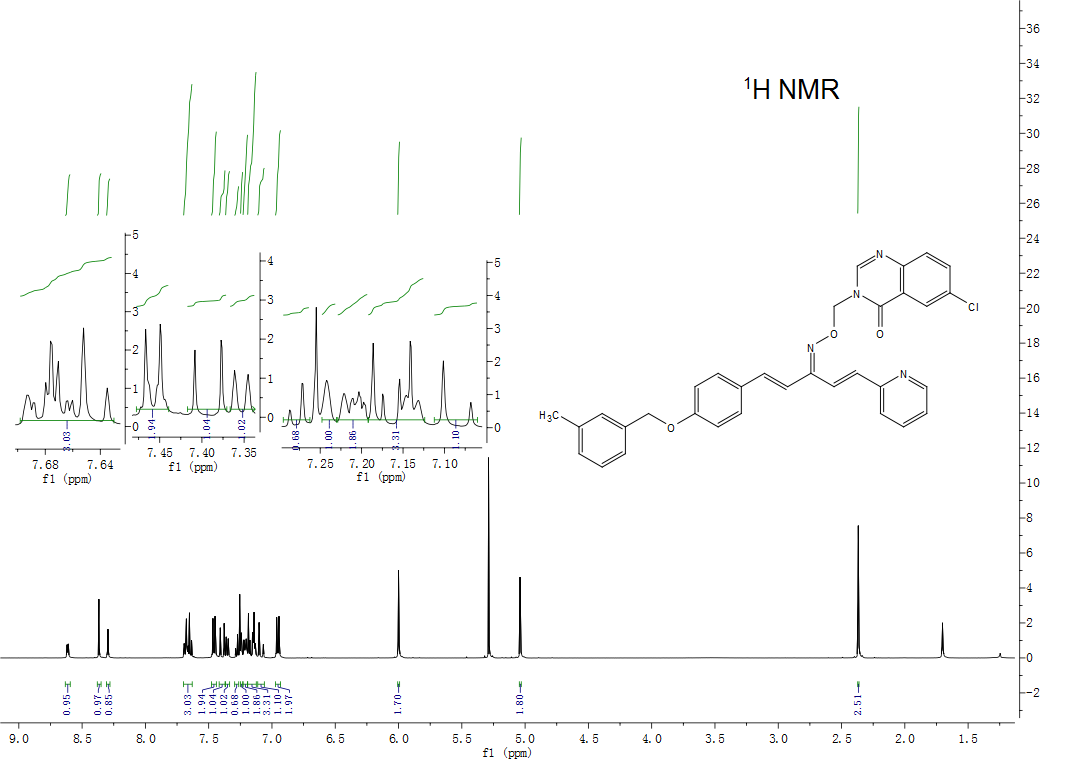


Figure S22. ^13^C NMR spectrum of compound 8f


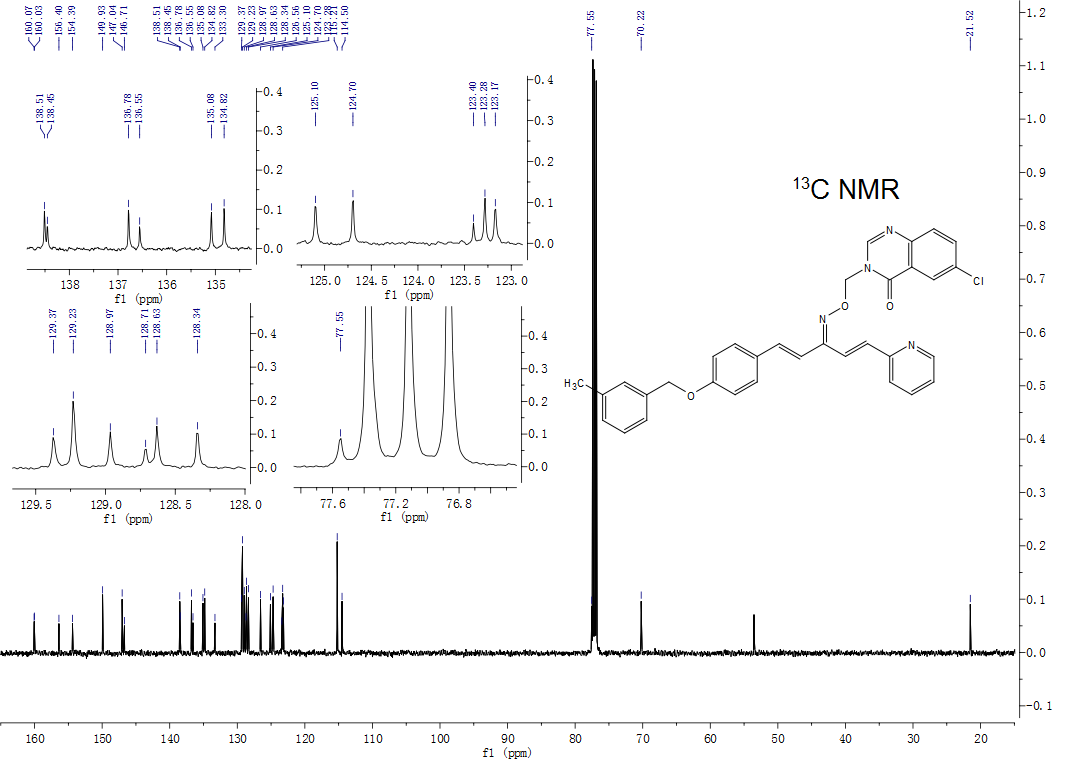


Figure S23. IR spectrum of compound 8f


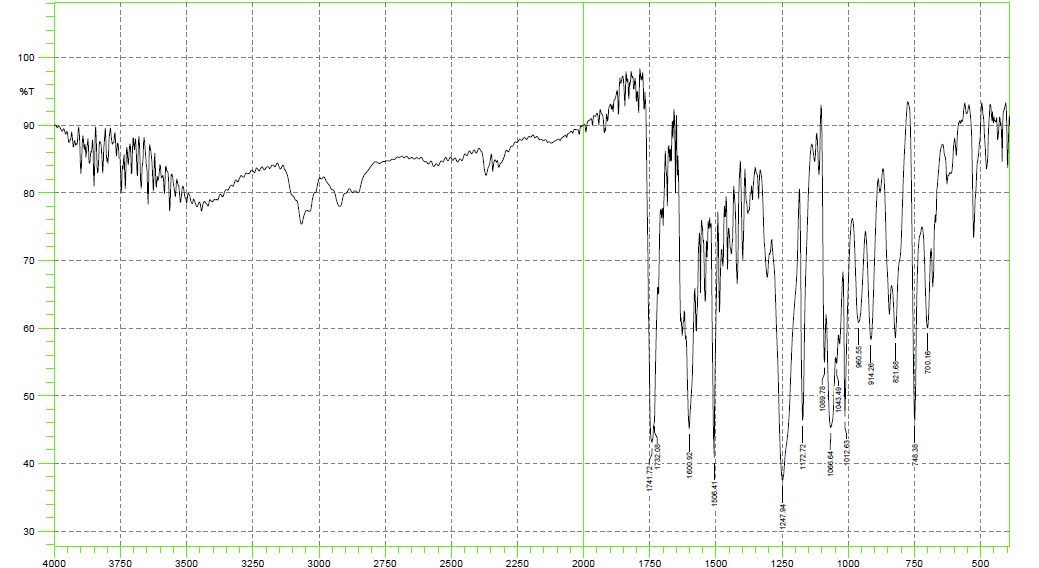


Figure S24.HRMS spectrum of compound 8f

Figure S25. ^1^H NMR spectrum of compound 8g


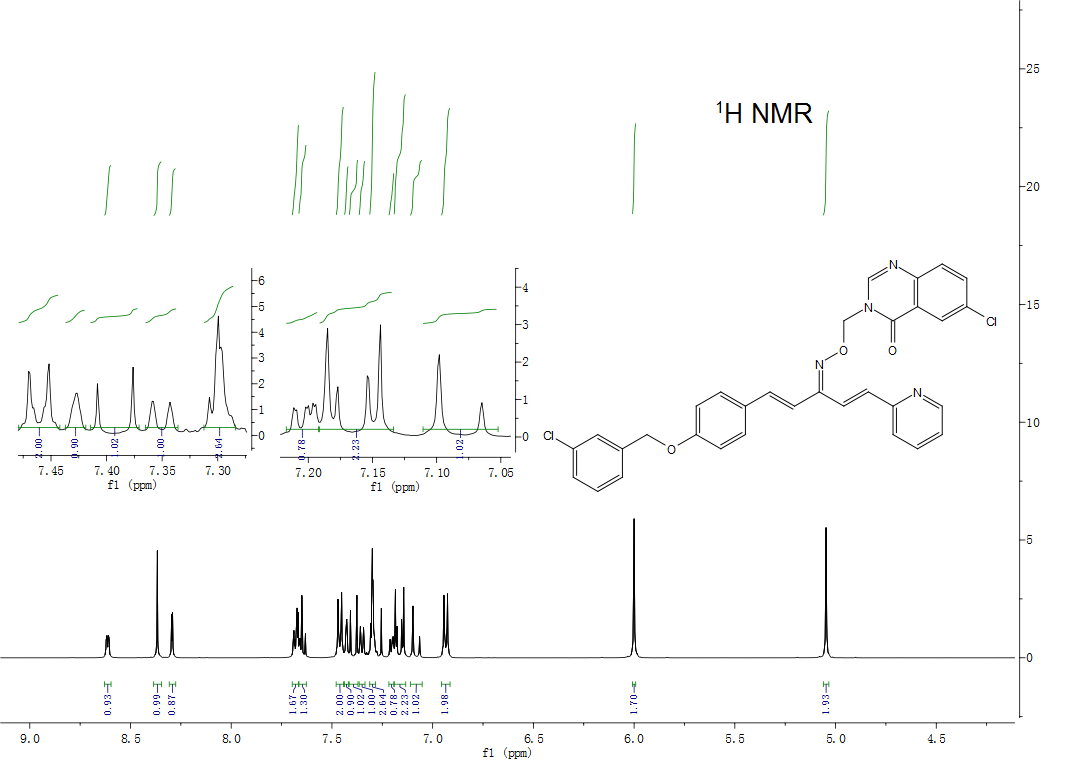


Figure S26. ^13^C NMR spectrum of compound 8g


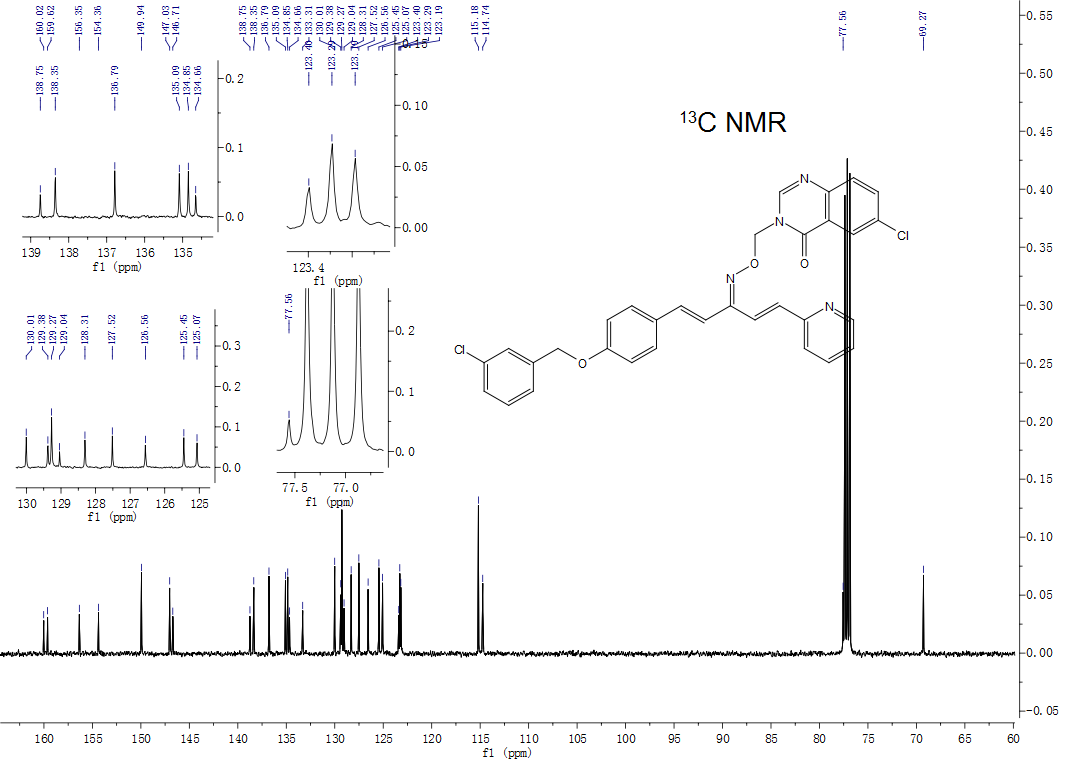


Figure S27. IR spectrum of compound 8g


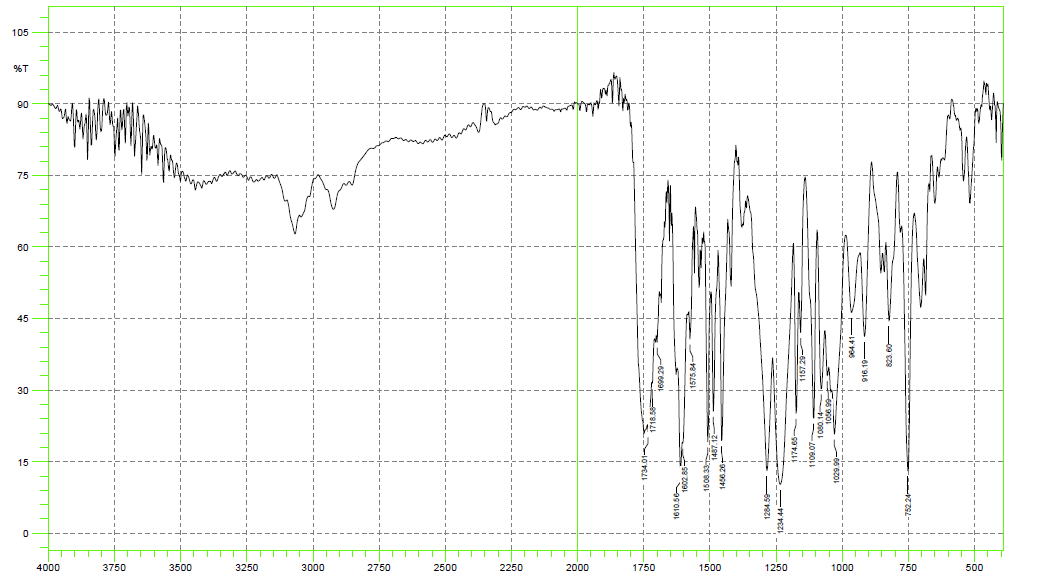


Figure S28. HRMS spectrum of compound 8g

Figure S29.^1^H NMR spectrum of compound 8h


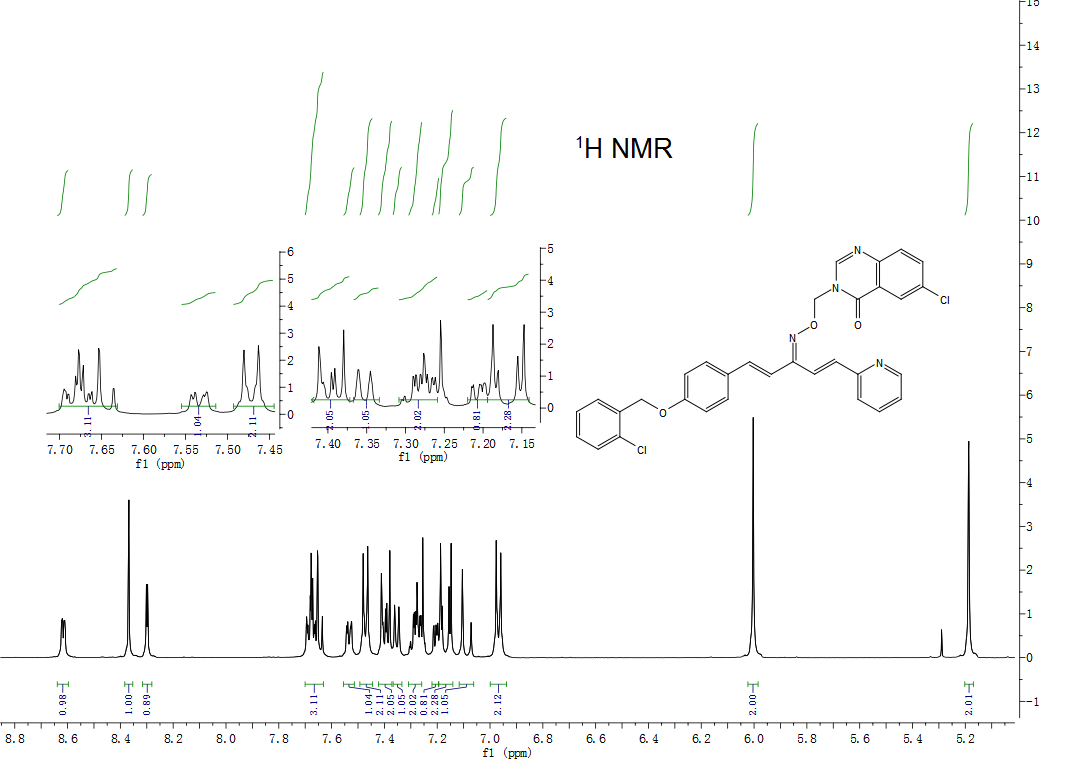


Figure S30.^13^C NMR spectrum of compound 8h


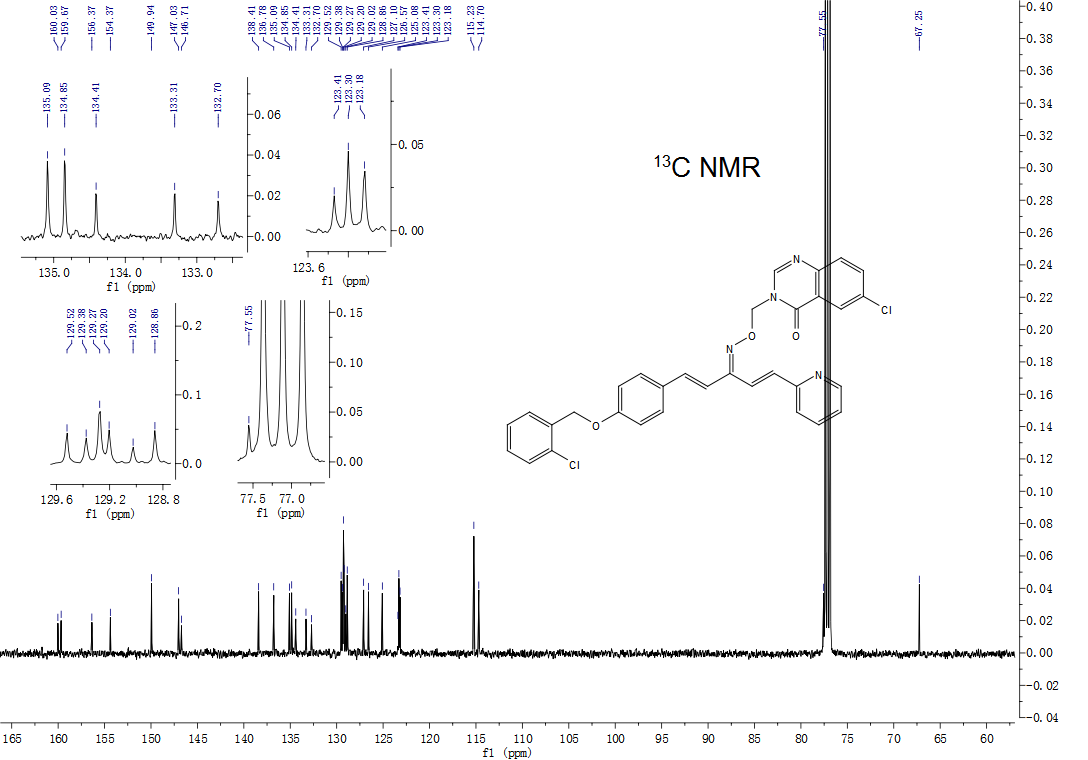


Figure S31.IR spectrum of compound 8h


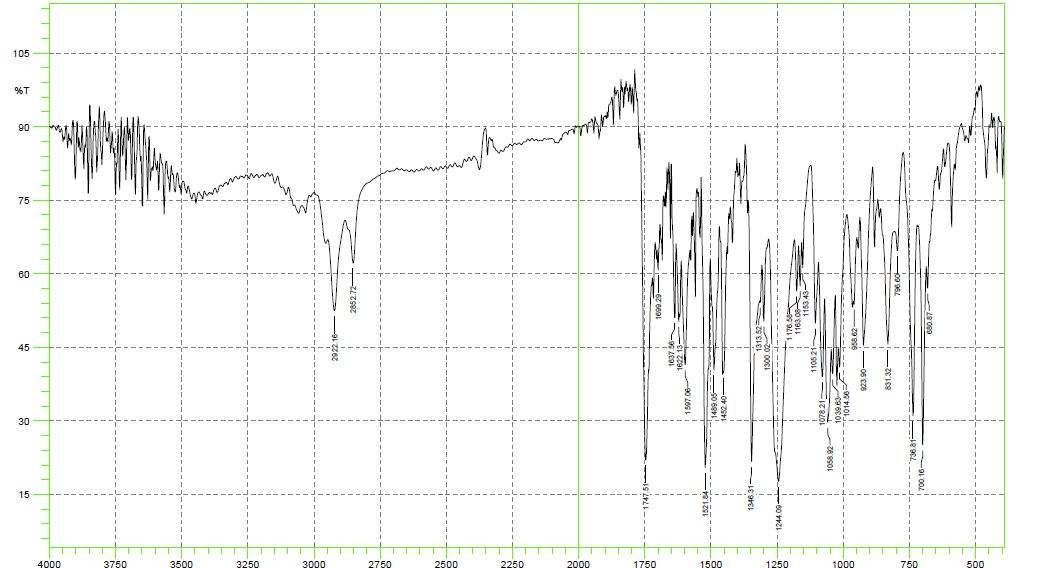


Figure S32.HRMS spectrum of compound 8h

Figure S33. ^1^H NMR spectrum of compound 8i


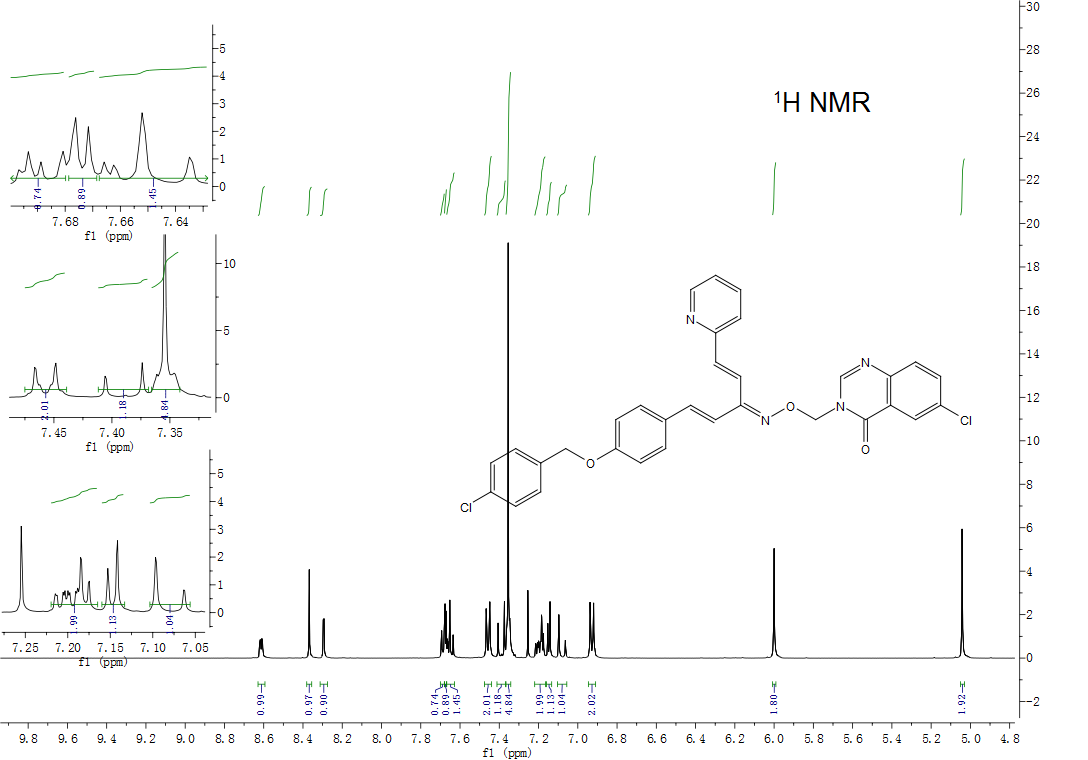


Figure S34. ^13^C NMR spectrum of compound 8i


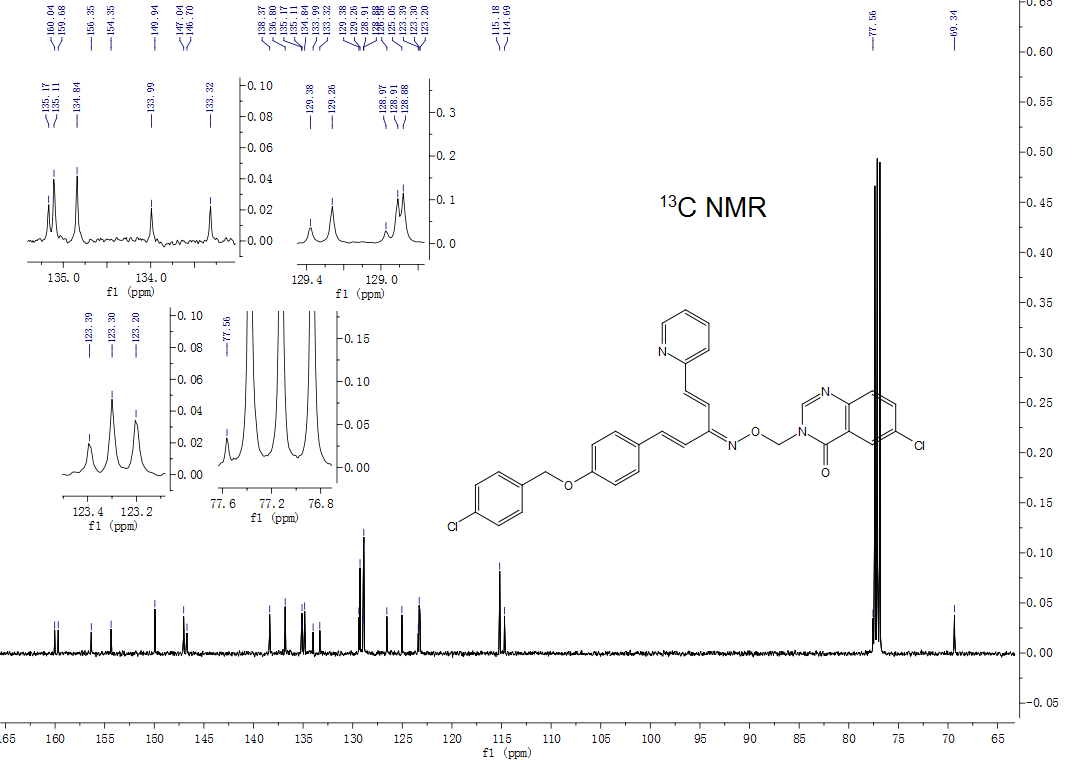


Figure S35.IR spectrum of compound 8i


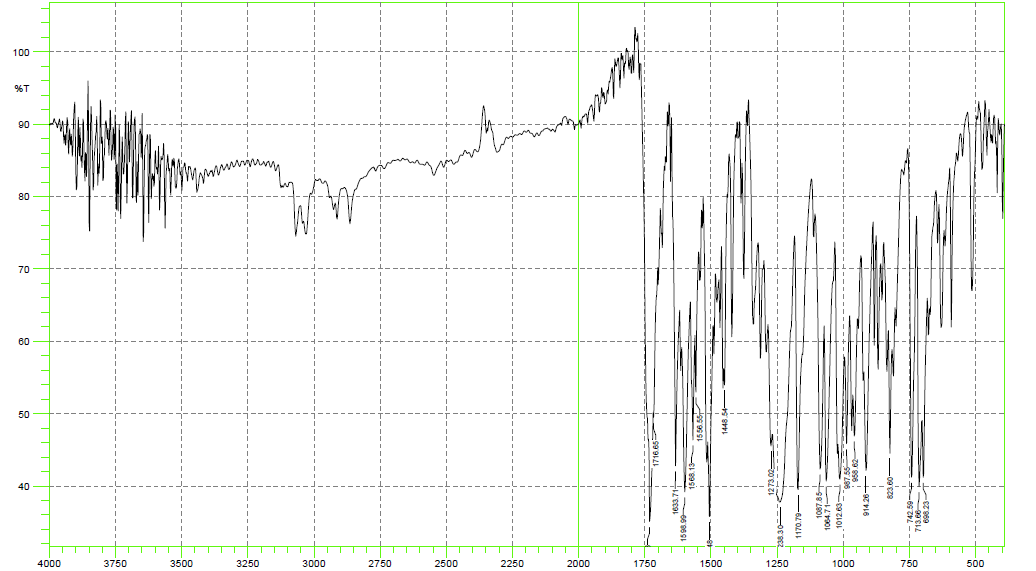


Figure S36. HRMS spectrum of compound 8i

Figure S37.^1^H NMR spectrum of compound 8j


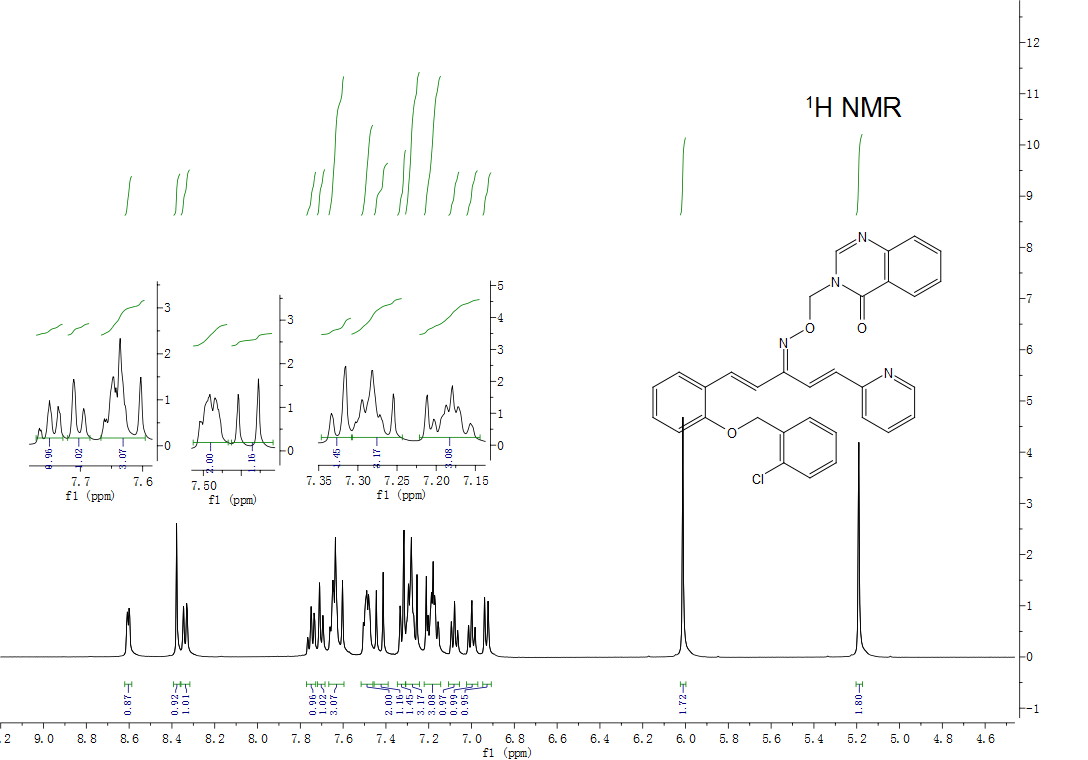


Figure S38.^13^C NMR spectrum of compound 8j


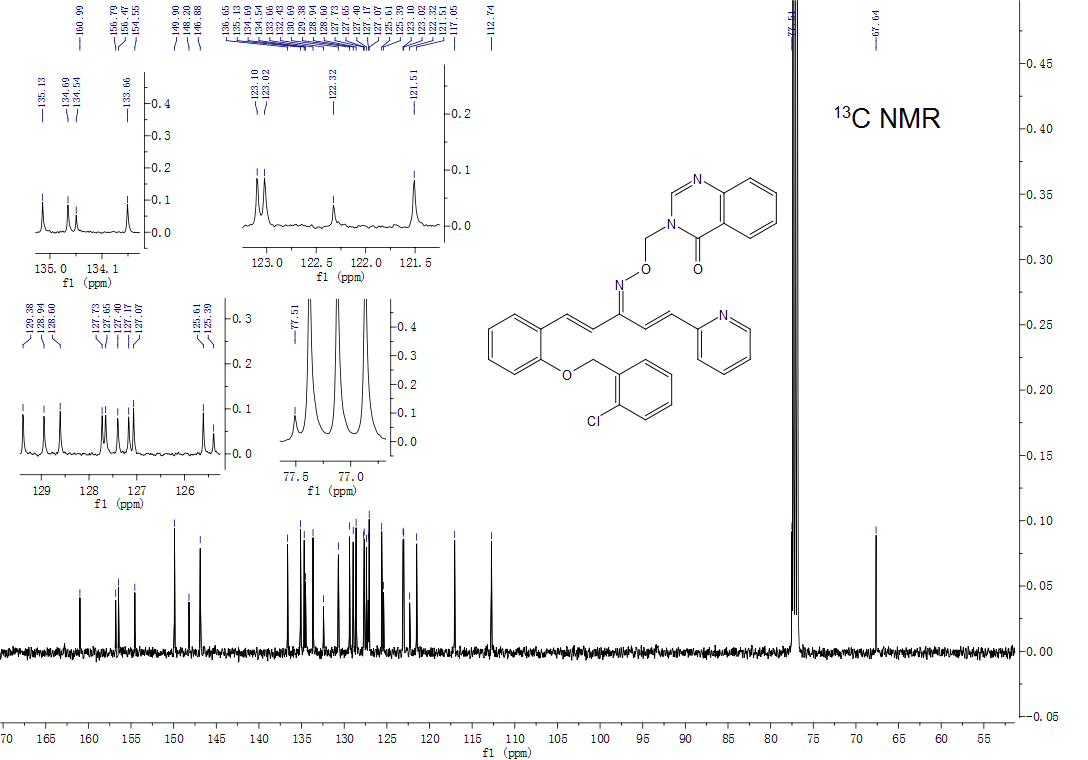


Figure S39.IR spectrum of compound 8j


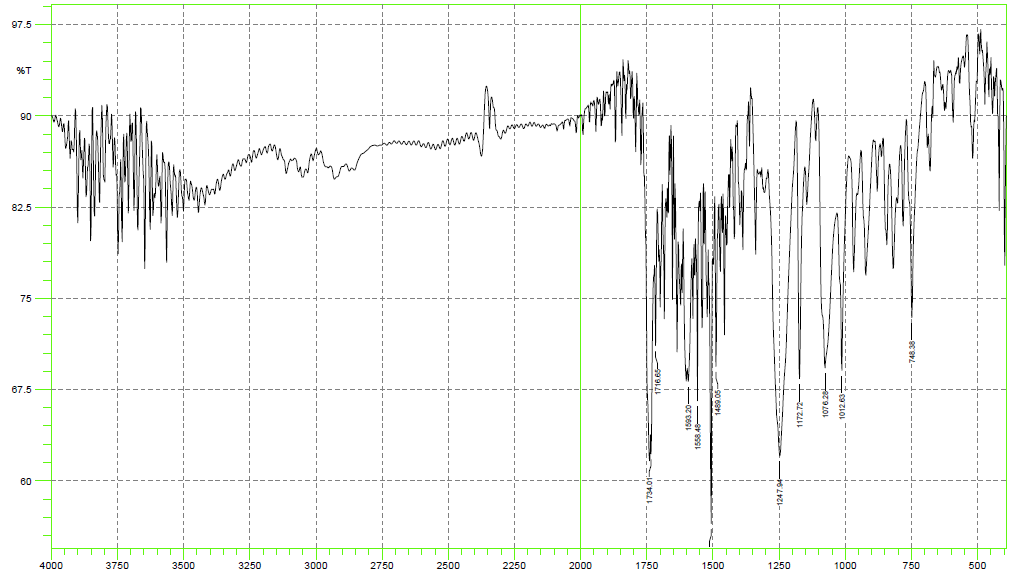


Figure S40. HRMS spectrum of compound 8j

Figure S41. ^1^H NMR spectrum of compound 8k


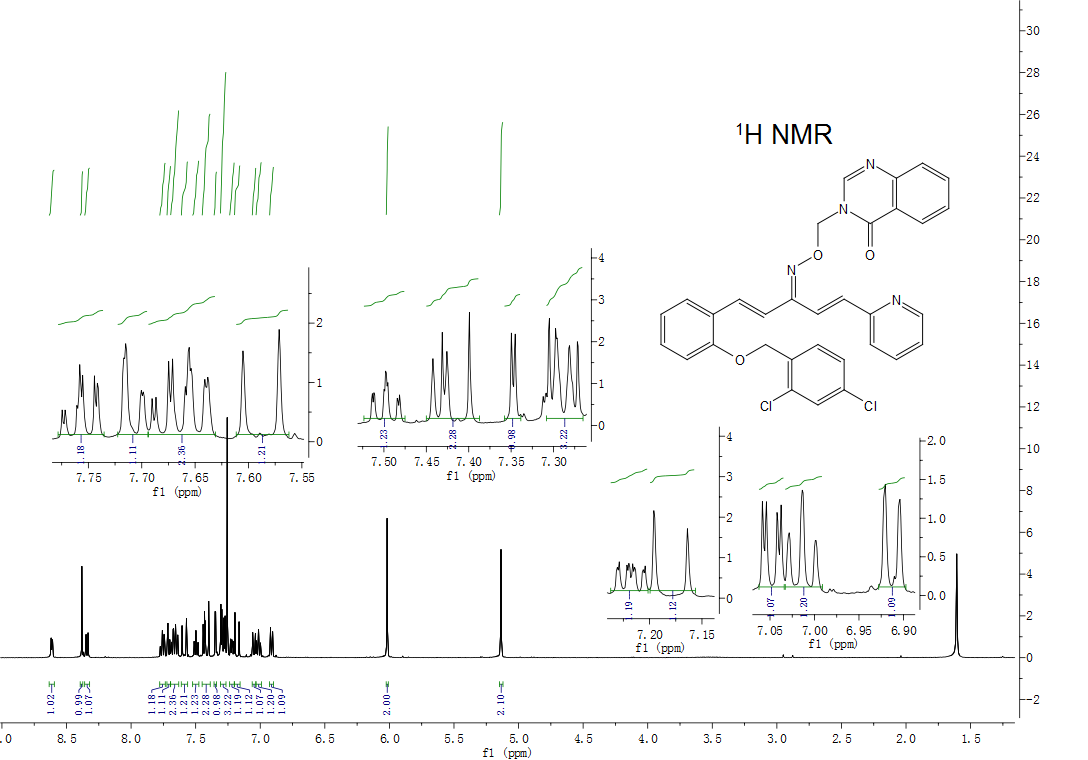


Figure S42. ^13^C NMR spectrum of compound 8k


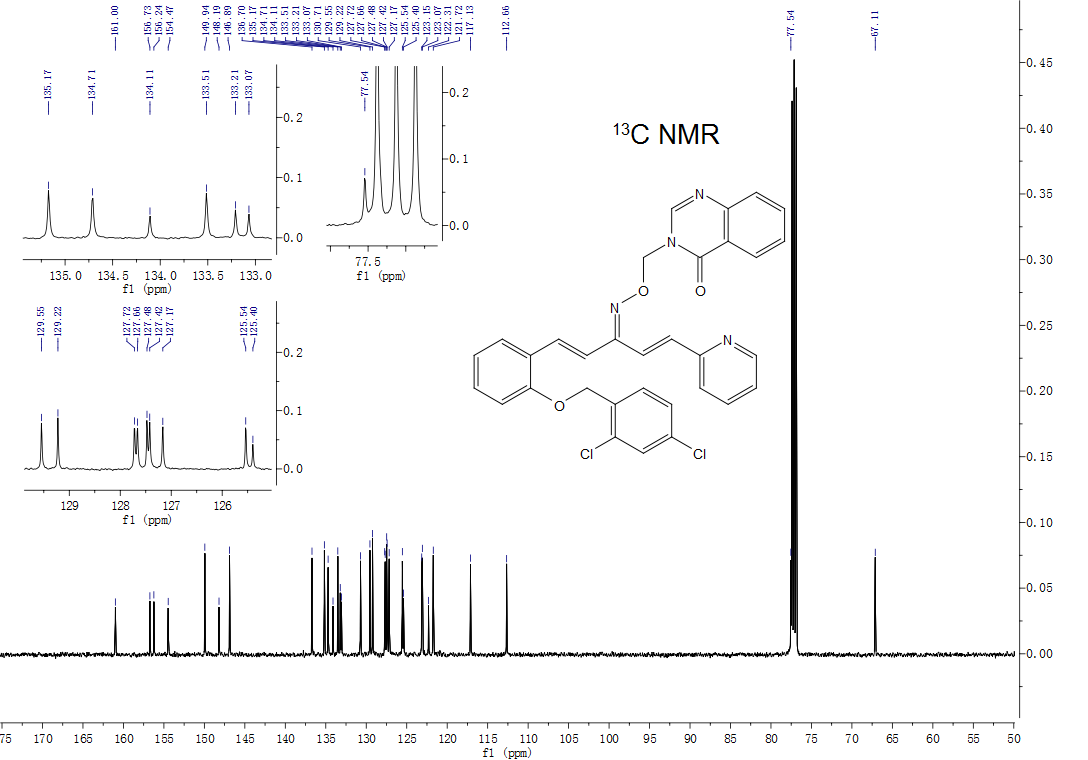


Figure S43.IRspectrum of compound 8k


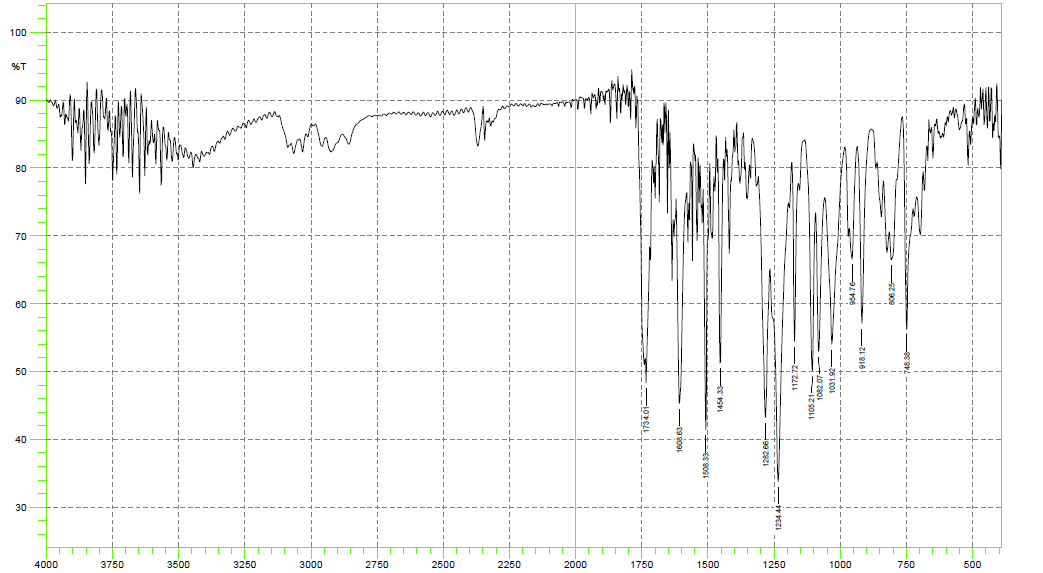


Figure S44. HRMS spectrum of compound 8k

Figure S45. ^1^H NMR spectrum of compound **8l**

**
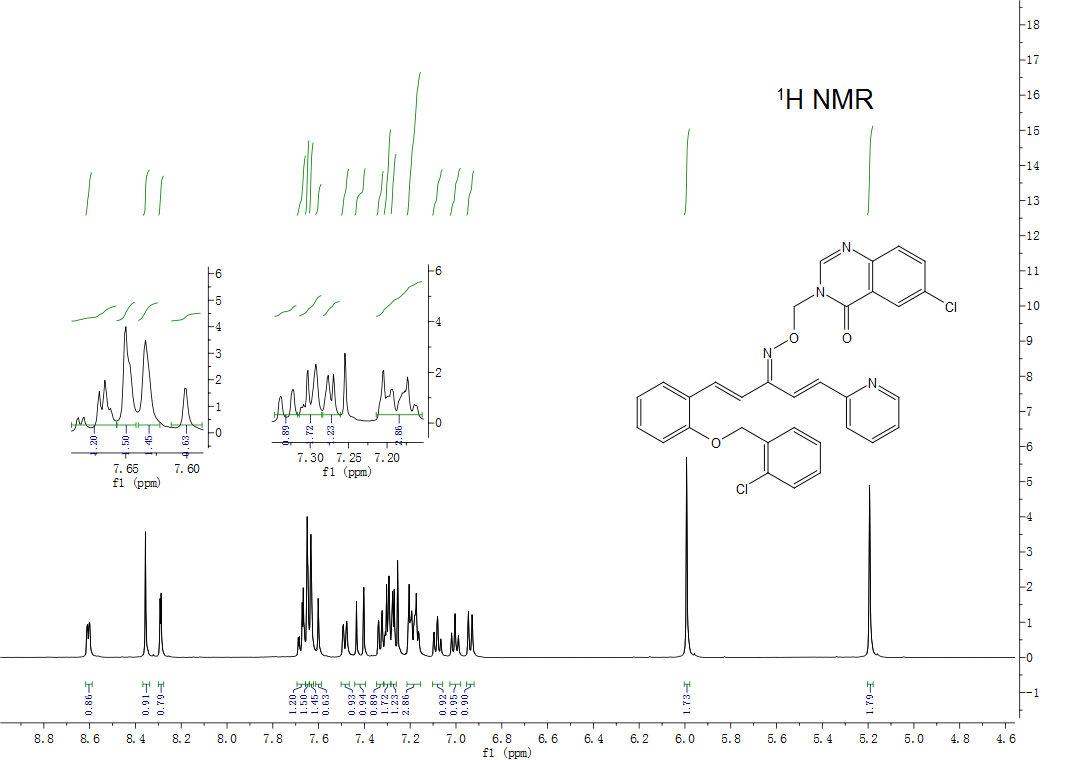
**

Figure S46. ^13^C NMR spectrum of compound **8l**

**
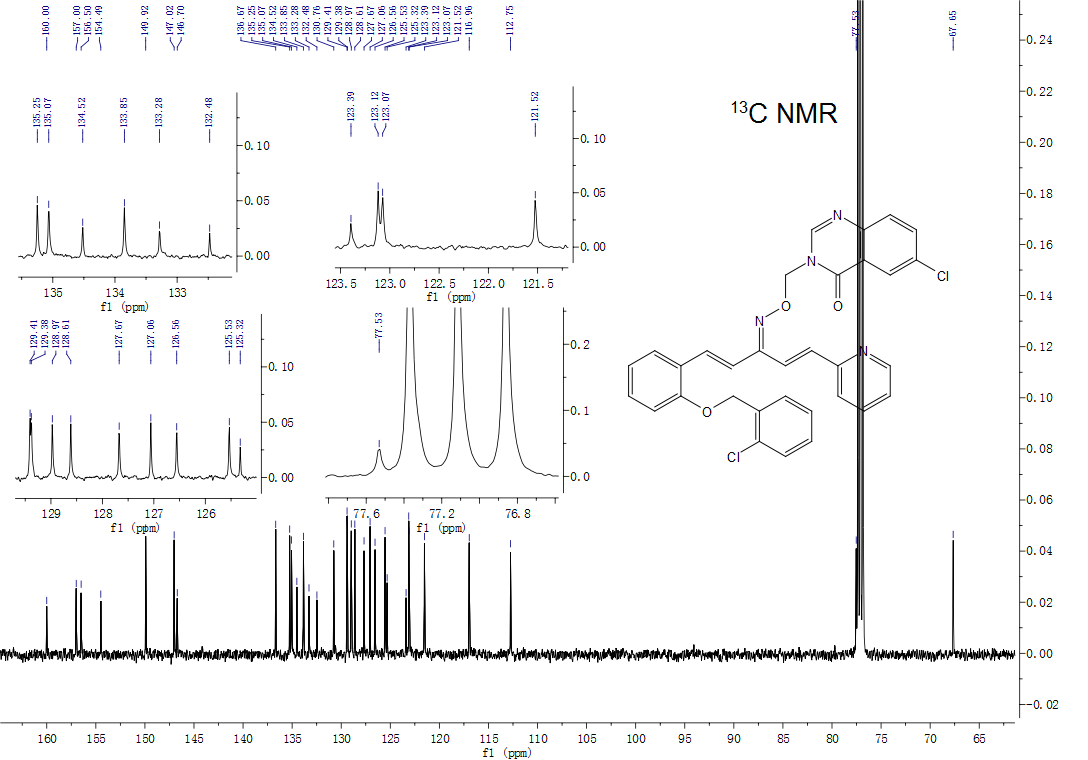
**

Figure S47. IR spectrum of compound **8l**

**
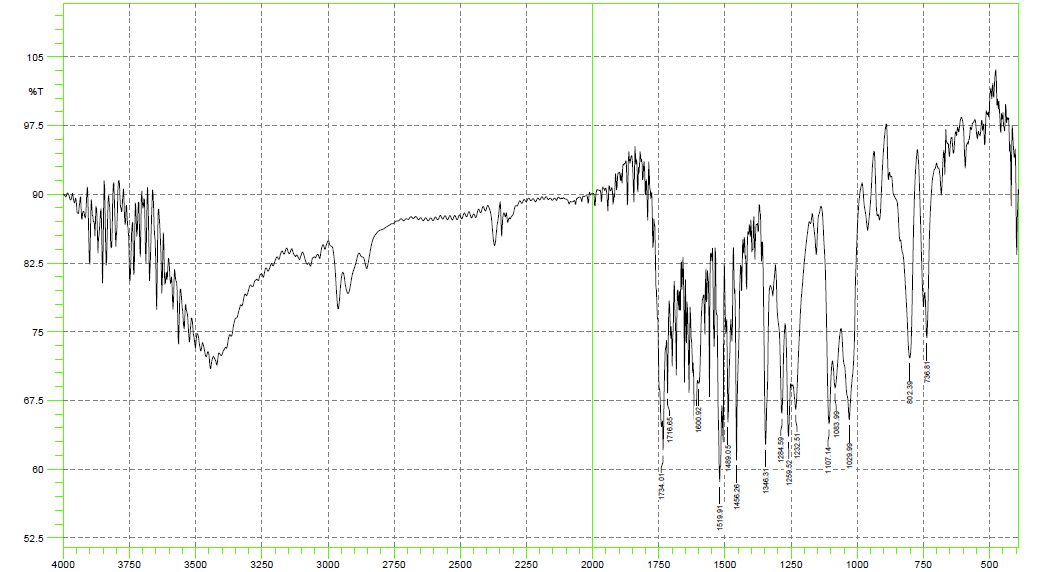
**

Figure S48. HRMS spectrum of compound **8l**

Figure S49. ^1^H NMR spectrum of compound **8m**

**
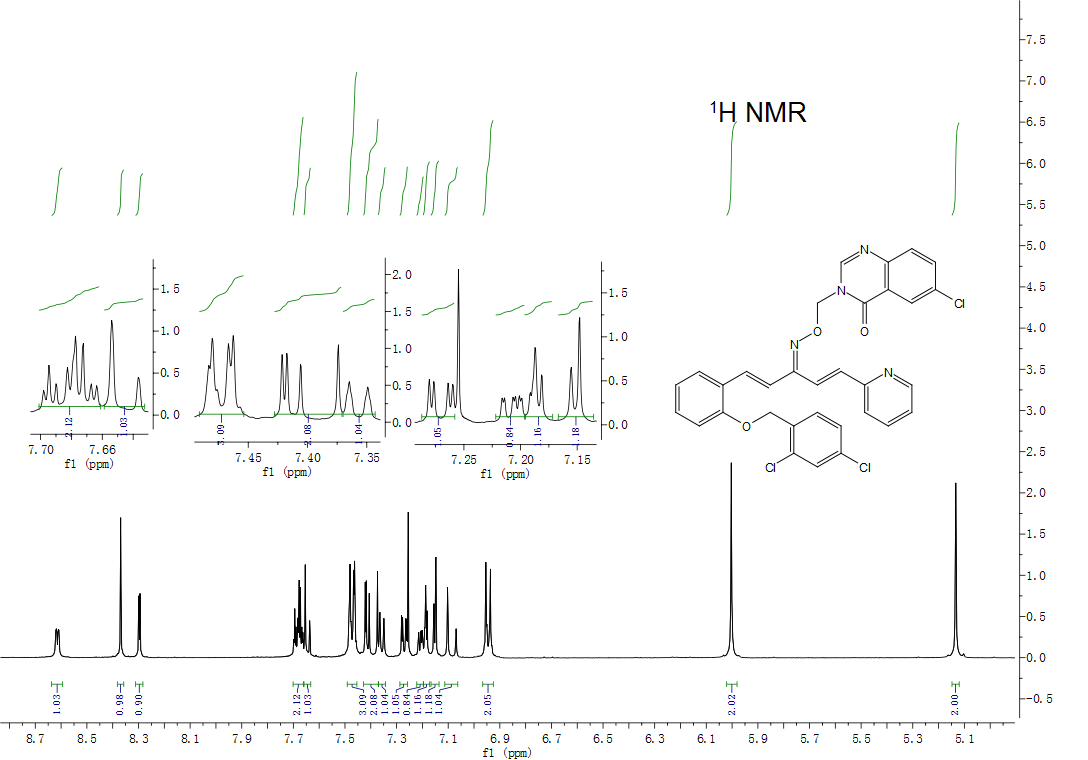
**

Figure S50. ^13^C NMR spectrum of compound **8m**

**
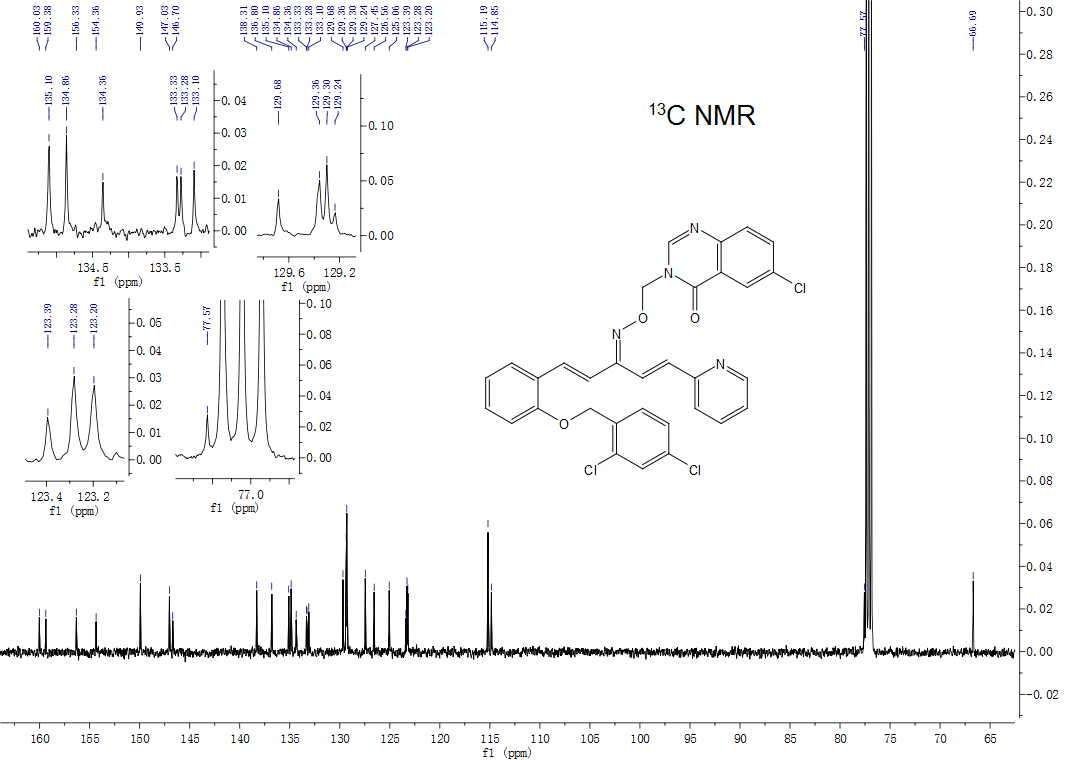
**

Figure S51. IR spectrum of compound **8m**

**
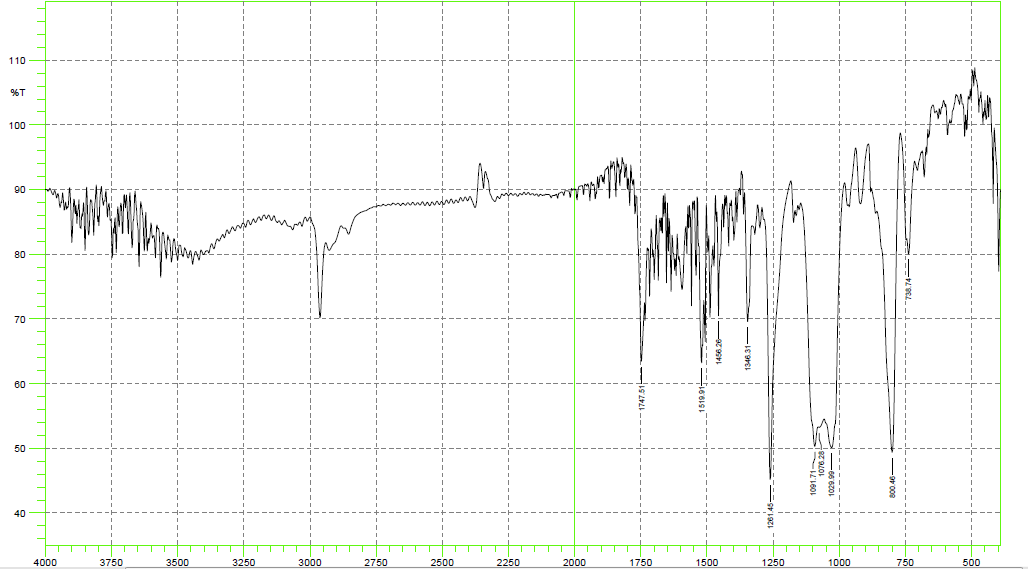
**

Figure S52. HRMS spectrum of compound **8m**

Figure S53. ^1^H NMR spectrum of compound **8n**

**
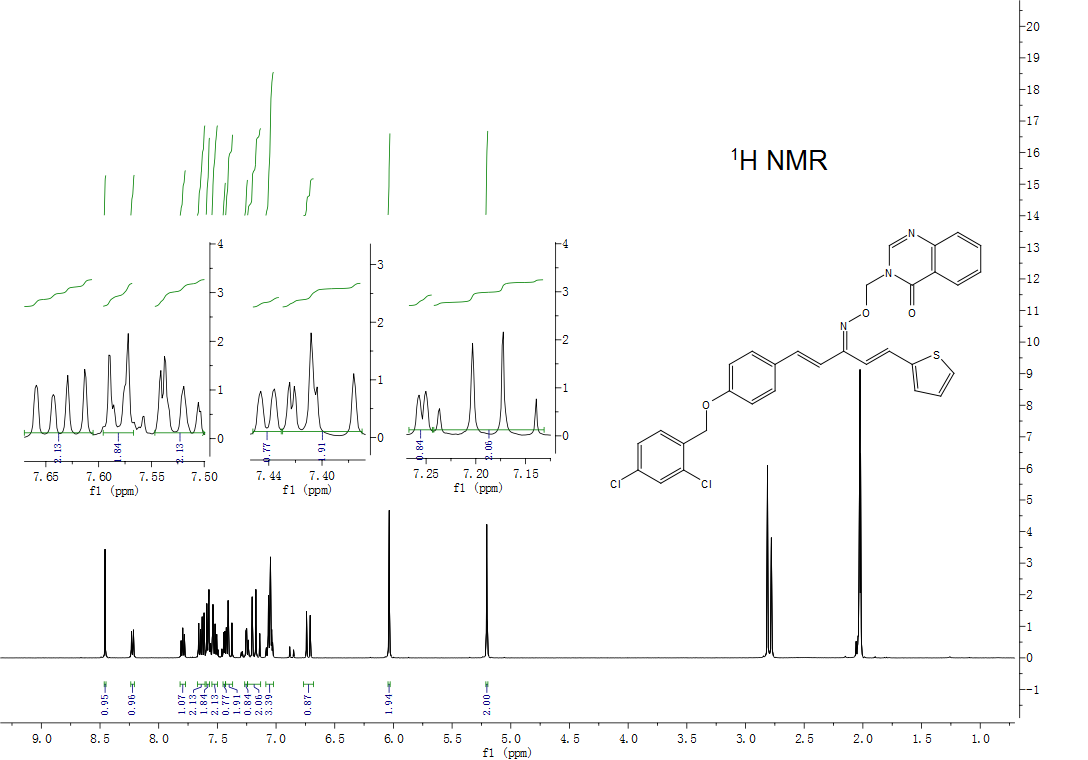
**

Figure S54. ^13^C NMR spectrum of compound **8n**

**
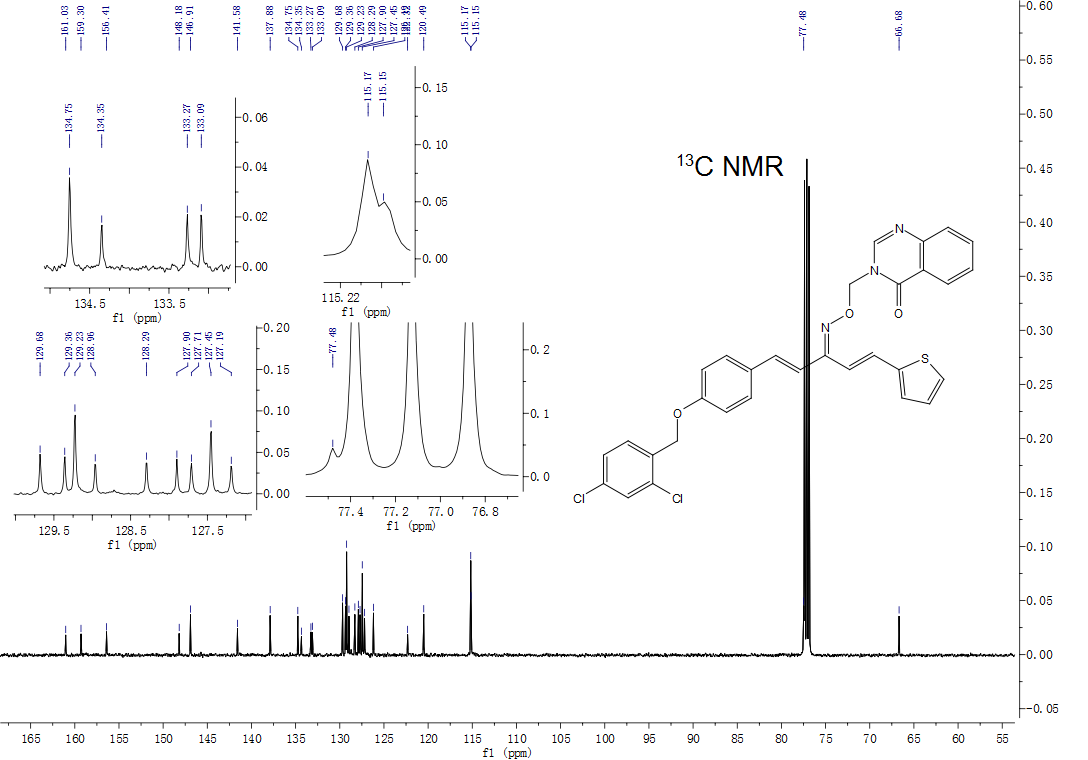
**

Figure S55. IR spectrum of compound **8n**

**
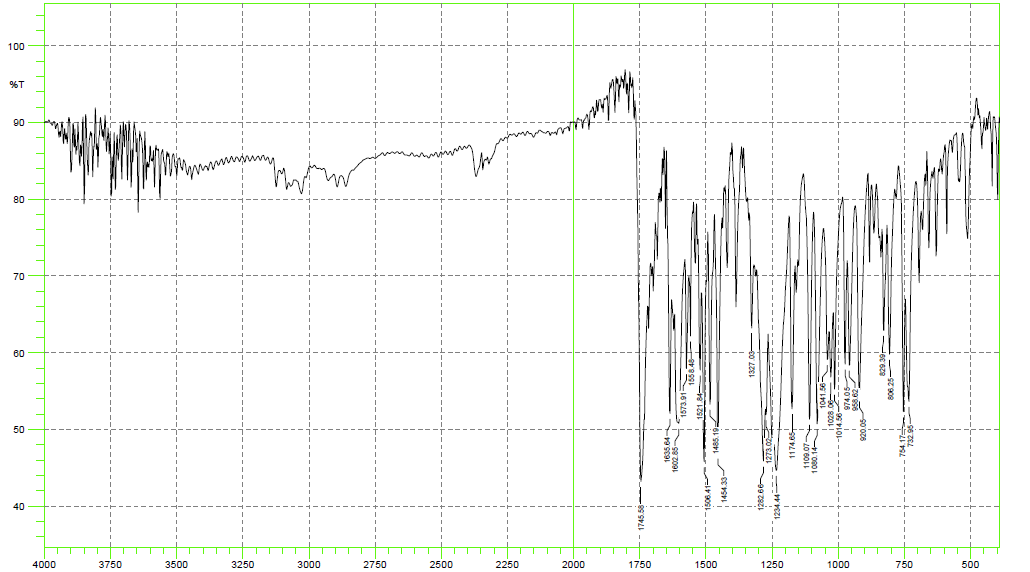
**

Figure S56. HRMS spectrum of compound **8n**

Figure S57. ^1^H NMR spectrum of compound **8o**

**
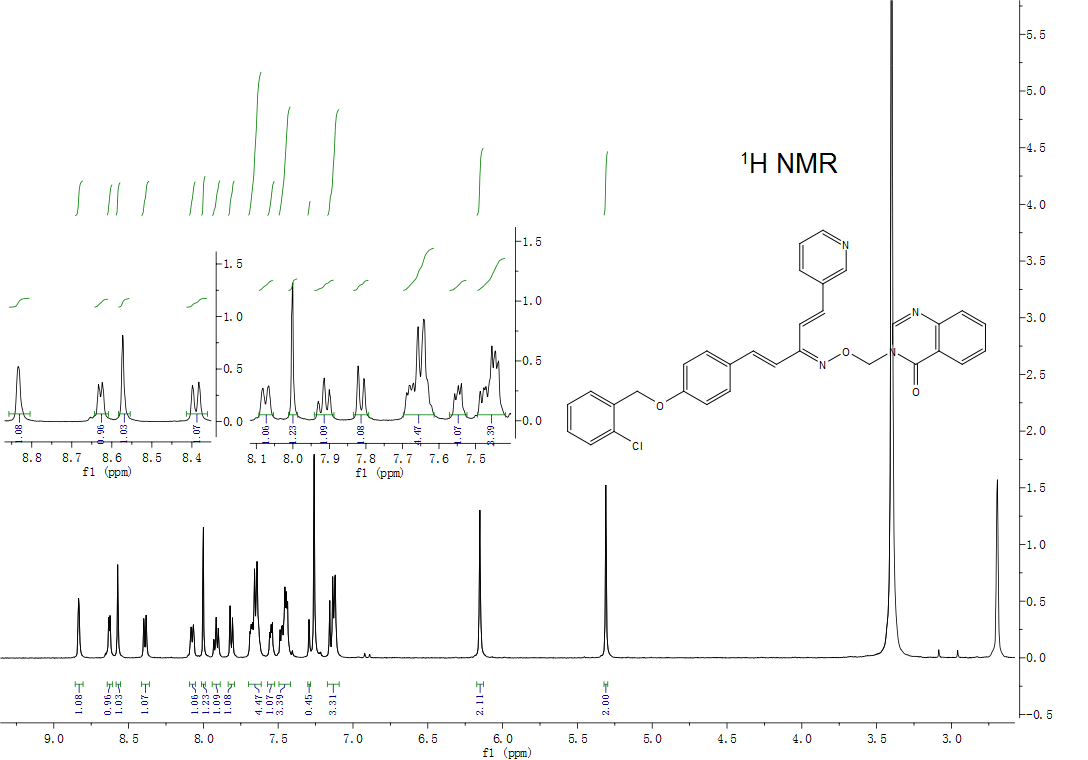
**

Figure S58. ^13^C NMR spectrum of compound **8o**

**
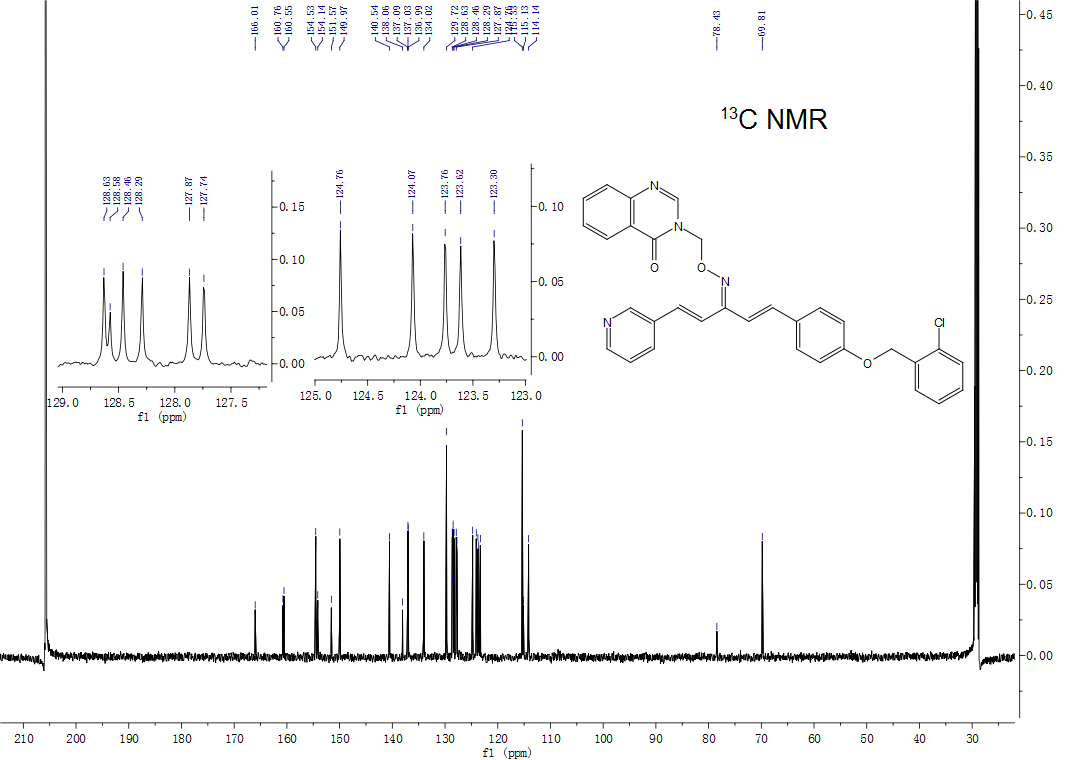
**

Figure S59. IR spectrum of compound **8o**

**
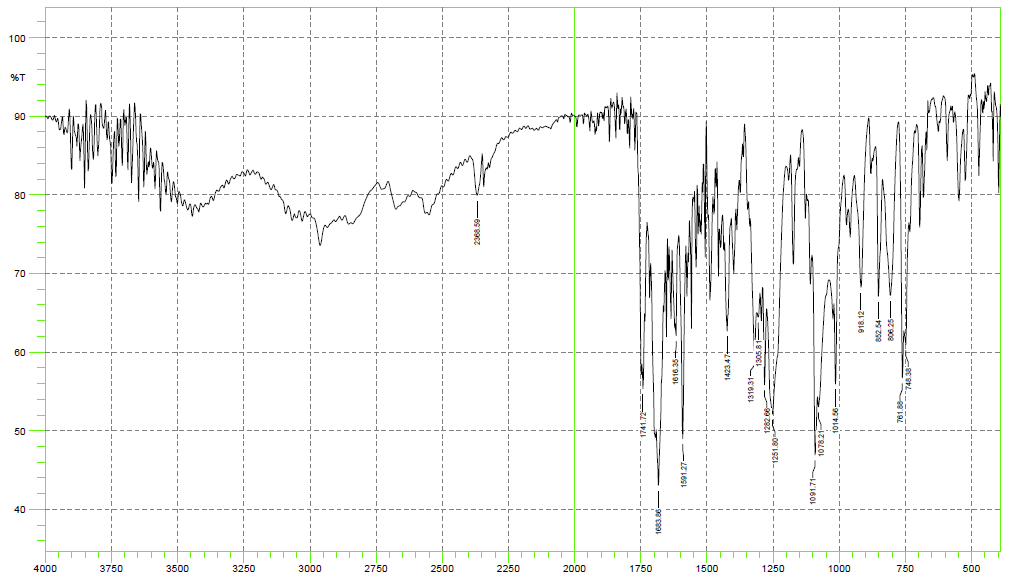
**

Figure S60. HRMS spectrum of compound **8o**

Figure S61. ^1^H NMR spectrum of compound **8p**

**
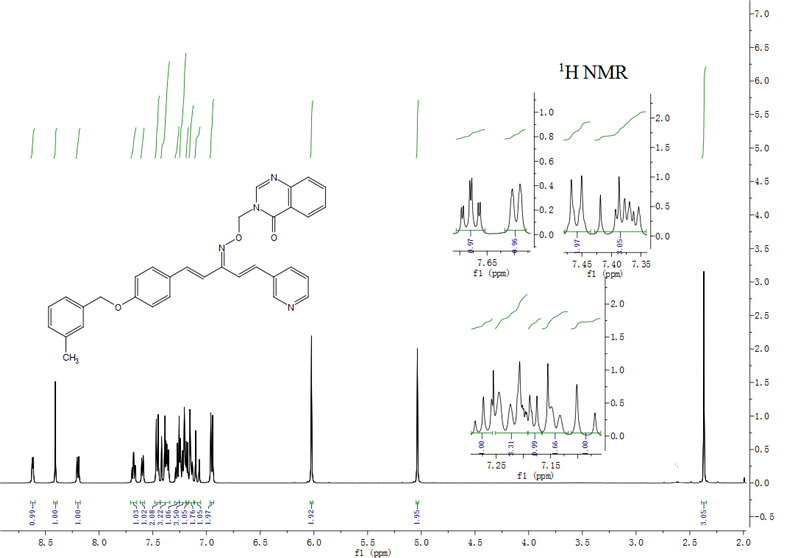
**

Figure S62. ^13^C NMR spectrum of compound **8p**

**
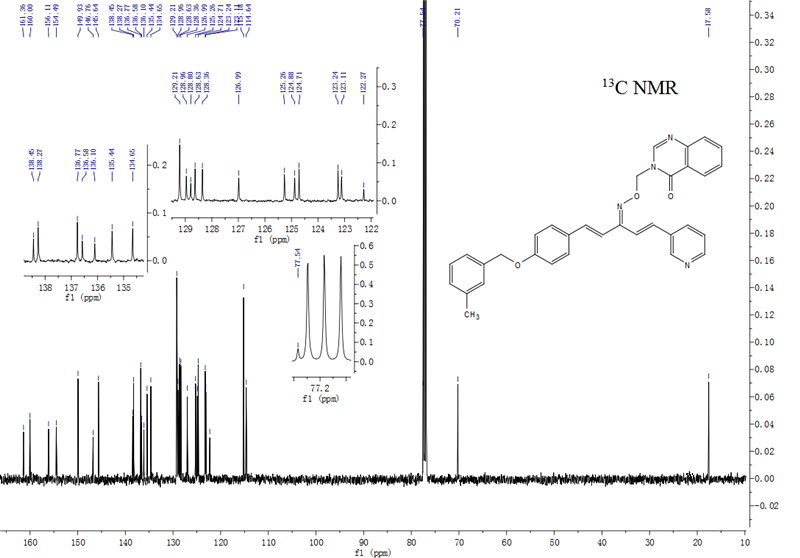
**

Figure S63.IR spectrum of compound **8p**

**
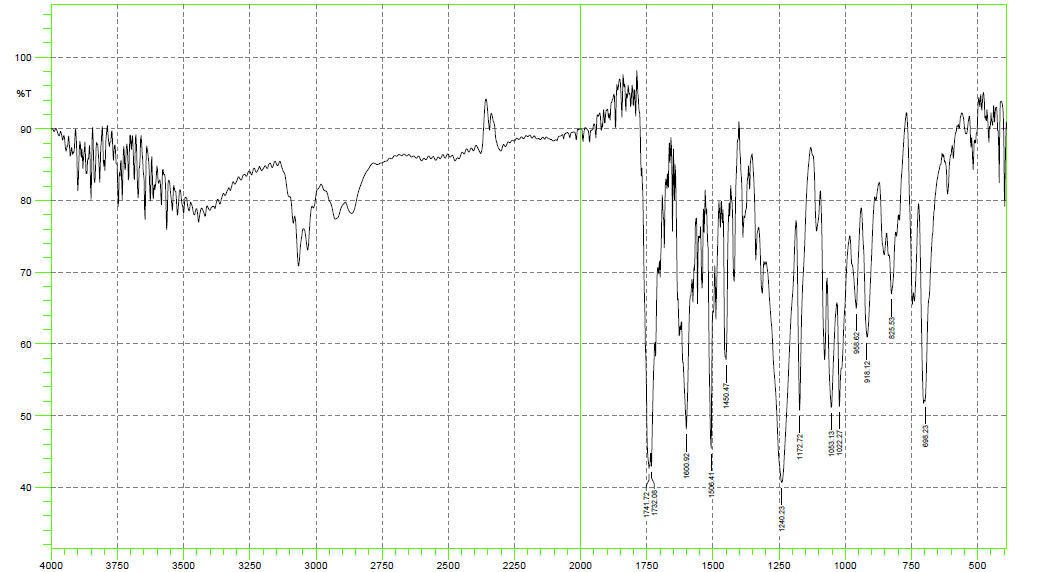
**

Figure S64. HRMS spectrum of compound **8p**

1. 

   **3. Activity confirmed of compound 8k**

   **
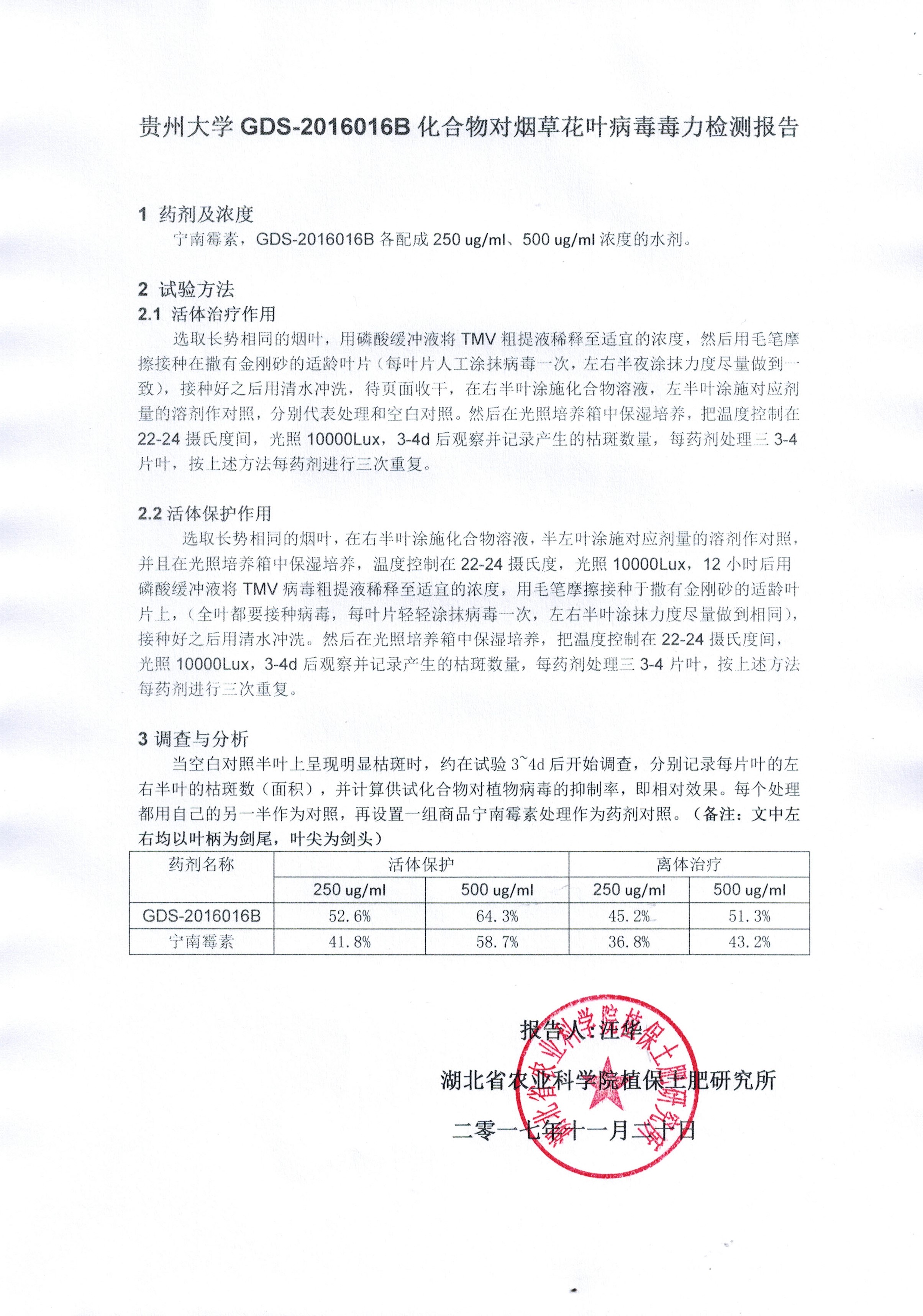
** [↑](#endnote-ref-2)
